# Automatic Sleep Spindle Detection and Genetic Influence Estimation Using Continuous Wavelet Transform

**DOI:** 10.3389/fnhum.2015.00624

**Published:** 2015-11-19

**Authors:** Marek Adamczyk, Lisa Genzel, Martin Dresler, Axel Steiger, Elisabeth Friess

**Affiliations:** ^1^Max Planck Institute of PsychiatryMunich, Germany; ^2^Centre for Cognitive and Neural Systems, University of EdinburghEdinburgh, UK; ^3^Donders Institute for Brain, Cognition and BehaviourNijmegen, Netherlands

**Keywords:** EEG, sleep spindle, automatic detection, twins, heritability

## Abstract

Mounting evidence for the role of sleep spindles in neuroplasticity has led to an increased interest in these non-rapid eye movement (NREM) sleep oscillations. It has been hypothesized that fast and slow spindles might play a different role in memory processing. Here, we present a new sleep spindle detection algorithm utilizing a continuous wavelet transform (CWT) and individual adjustment of slow and fast spindle frequency ranges. Eighteen nap recordings of ten subjects were used for algorithm validation. Our method was compared with both a human scorer and a commercially available SIESTA spindle detector. For the validation set, mean agreement between our detector and human scorer measured during sleep stage 2 using kappa coefficient was 0.45, whereas mean agreement between our detector and SIESTA algorithm was 0.62. Our algorithm was also applied to sleep-related memory consolidation data previously analyzed with a SIESTA detector and confirmed previous findings of significant correlation between spindle density and declarative memory consolidation. We then applied our method to a study in monozygotic (MZ) and dizygotic (DZ) twins, examining the genetic component of slow and fast sleep spindle parameters. Our analysis revealed strong genetic influence on variance of all slow spindle parameters, weaker genetic effect on fast spindles, and no effects on fast spindle density and number during stage 2 sleep.

## Introduction

Sleep spindles are one of the hallmarks in electroencephalographic (EEG) signal during non-rapid eye movement (NREM) sleep. They are characterized as bursts of rhythmical activity in the 10–16 Hz frequency range, with waxing and waning shapes lasting usually from 0.5–2.5 s. There are two types of sleep spindles. The so-called fast spindles are mainly present in parietal brain regions, whereas slow spindles predominate in frontal areas. Low-resolution electromagnetic tomography (LORETA) demonstrated a distributed slow spindle source in the prefrontal cortex and a fast spindle source in the precuneus (Anderer et al., 2001). However, both spindle types are generated via thalamic-cortical loops (Astori et al., 2013). The average slow spindle peak is 11.5 Hz and fast spindle peak is 13 Hz, with large inter-subject variation (Werth et al., 1997).

There is a mounting evidence for the role of sleep spindles in neuroplasticity. Increased spindle density and activity was observed after both declarative and procedural learning (Gais et al., 2002; Morin et al., 2008). Increases in spindle activity were also reported to positively correlate with memory retention (Clemens et al., 2005; Nishida and Walker, 2007; Genzel et al., 2009; Cox et al., 2012). These oscillations provide excellent conditions for long-term synaptic changes (Buzsáki, 1989; Fogel and Smith, 2011), and the interplay of spindles and hippocampal ripples plays an important role in neuroplasticity (Clemens et al., 2007; Genzel et al., 2014). Specifically, spindles deafferent the cortex from the hippocampus, enabling local processing of increased firing rates in the cortex in response to hippocampal firing during ripples (Peyrache et al., 2009; Wierzynski et al., 2009; Genzel et al., 2014) and may additionally serve a role in cortical plasticity processes that are independent of hippocampal-led replay (Andrillon et al., 2011; Genzel et al., 2014). Sleep spindles have also been proposed to represent a biomarker of learning trait and intelligence (Fogel and Smith, 2011), however the strength of this association has recently been doubted (Ujma et al., 2014). Furthermore, impaired sleep spindle activity was shown in various psychiatric disorders (Astori et al., 2013). Reduced spindle activity was reported in patients with schizophrenia (Ferrarelli et al., 2007, 2010; Wamsley et al., 2012), affective disorders (de Maertelaer et al., 1987; Lopez et al., 2010) and Alzheimer’s disease (Montplaisir et al., 1995), and these diseases also showed impaired sleep related memory consolidation (Dresler et al., 2010, 2011; Genzel et al., 2011, 2015).

In view of the putative potential of sleep spindles as biomarkers, their heritability is of interest. Previous studies showed that the NREM sleep power spectrum in the sleep spindles frequency range has finger-print characteristics (De Gennaro et al., 2005; Buckelmüller et al., 2006) and is heritable (Ambrosius et al., 2008; De Gennaro et al., 2008), suggesting that sleep spindle activity is also heritable. However, this “spindle-print” on the power spectrum is influenced by a number of mixed slow and fast spindle characteristics: their frequency, amplitude and amount. Therefore, we decided to investigate the heritability of sleep spindle basic characteristics in detail. For this purpose we developed, validated and applied a new spindle detection algorithm to our twin data.

A number of spindle detection algorithms are already published. One of the first was presented by Schimicek et al. (1994). This method uses a band-pass filter (pass-band: 11.5–16 Hz) and detects spindles with a fixed amplitude threshold (peak-to-peak amplitude of 25 μV). Later algorithms proposed a diversity of solutions to better “extract” sleep spindles from the signal as well as to handle high inter-subject variability in sleep spindle frequency and EEG signal amplitude. One of the approaches to improve the extraction of spindle shapes from the signal is the application of a wavelet transform (WT) instead of a band-pass filter (Zygierewicz et al., 1999; Latka et al., 2005; Wamsley et al., 2012). The outcome of a WT depends not only on the power in a given frequency, but also on the shape of graphoelements in the signal, and therefore may be more specific than band-pass filtering (Addison, 2002). The other approach that considers waxing and waning shape of sleep spindles is the application of two thresholds, from which the higher one is used to localize activity bursts in sigma frequency and the lower one to estimate the duration of sleep spindles (Ferrarelli et al., 2007). Another challenge in sleep spindle detection is the variation in EEG signal amplitude between subjects, but also channels. Reasons for this phenomenon can be of a technical nature (movements during the measurement period influencing electrode placement, differences in electrode impedance) as well as physiological. EEG signal decreases with age (Dijk et al., 1989b), and is higher in females compared to males (Dijk et al., 1989a). For this reason, spindle detection threshold in many algorithms is set individually according to various characteristics of analyzed EEG signal: for example through the average amplitude in individually localized spindle frequency range (Bódizs et al., 2009; Ujma et al., 2015) or the amplitude of pre-localized spindle candidates (Huupponen et al., 2007). Furthermore, inter-subject variation in slow and fast spindle frequency reported by Werth et al. (1997) suggests that these frequency ranges should be adjusted individually in order to discriminate between fast and slow spindles. Bódizs et al. (2009, 2012) proposed to estimate spindle frequency ranges using pre-computed average frequency spectra in the 9–16 Hz range. Slower and faster sigma peaks are usually dominant over the frontal and parietal derivations, respectively. For this reason, normalized frequency spectra for frontal and parietal EEG channels were compared and a peak higher in the frontal EEG spectrum was considered a slow spindle peak whereas a peak higher in the parietal EEG spectrum was considered a fast spindle peak.

Due to inter-subject variation in slow and fast spindle frequency, as well as in signal amplitude, spindle detection is a challenging task. It was shown recently that agreement between algorithms and humans is surprisingly low (Warby et al., 2014). Proper separation between slow and fast spindles seems to be very important, since these two types of spindles may play different roles in sleep-dependent memory processing (Mölle et al., 2011). For this reason, our aim was to develop a spindle detector which acknowledges considerable inter-subject variability in sleep spindle activity. In our algorithm we combined previously published methodological solutions with our proposal of detection thresholds adjustment and estimation of spindle frequency ranges. We compared spindle detection of our new algorithm with both a human scorer and a commercially available SIESTA spindle detector (Anderer et al., 2005). Considerable detection differences between the algorithms raises the question on how different methods could influence the interpretation of previous findings. In order to investigate this further, we applied our algorithm to sleep-related memory consolidation data, which were already analyzed with the SIESTA algorithm and revealed a positive correlation between spindle activity and declarative memory consolidation (Genzel et al., 2009). Finally, we analyzed a twin study comparing slow and fast sleep spindle parameters: total count, density, amplitude, duration and frequency between healthy monozygotic (MZ) and dizygotic (DZ) twins.

## Materials and Methods

Almost all computations were performed using MATLAB 2014a. Only MANCOVA analysis was performed using SPSS v17. The source code is available from the corresponding author.

### Validation Sample—Nap Recordings

Our algorithm was validated with data from an earlier study (Genzel et al., 2014). In brief, 20 participants (10 male, age 20–30 years) had two nap sessions in the sleep laboratory separated by at least 4 weeks, one with and one without previous learning experience. For more details regarding study design and participants please see Genzel et al. (2014). Eighteen naps from *n* = 10 subjects were randomly selected and our algorithm was compared with the SIESTA algorithm of Anderer et al. (2005) and with a human scorer. Sleep spindle scoring was performed by a trained research assistant and double-checked by an experienced sleep expert. The experimental protocol was approved by the Ethics Committee of the Ludwigs Maximilian University, Faculty of Medicine, Munich and written informed consent was obtained from the participants.

### Sleep-Related Memory Consolidation Sample

The data of the memory consolidation study were described by Genzel et al. (2009). Recruited subjects were *n* = 12 healthy volunteers, six males and six females. Age ranged between 20–30 years. Prerequisites for inclusion and exclusion criteria as well as study protocol are described in detail elsewhere (Genzel et al., 2009). Briefly, the subjects spent six nights in our sleep laboratory, where three nights served as adaptation nights which were followed by study nights. Each experimental session consisted of adaptation night, learning before the study night (declarative memory: finger tapping task, procedural memory: verbal paired associates task), study recording with various experimental sleep conditions [REM sleep deprivation, slow wave sleep (SWS) deprivation and undisturbed night] and a retest after two nights of recovery sleep. EEG recordings from the undisturbed study night were used for sleep spindle analysis. The experimental protocol was approved by the Ethics Committee of the Ludwigs Maximilian University, Faculty of Medicine, Munich and written informed consent was obtained from the participants.

### Twin Sample

We analyzed the data of the twin study described by Ambrosius et al. (2008). We recruited *n* = 35 pairs of MZ and *n* = 14 pairs of DZ twins. All twin pairs had been raised together. The twins underwent physical, psychiatric, and laboratory examinations to exclude acute and chronic diseases. Prerequisites for inclusion and determination of zygosity are described in detail elsewhere (Ambrosius et al., 2008). Due to technical reasons (high EEG amplitude differences in consecutive nights) 3 MZ pairs were excluded. All presented results have been obtained from the remaining 32 pairs of MZ twins (mean (SD): 23.8 (4.8) years; range: 17–43 years, 16 male pairs, 16 female pairs) and 14 pairs of DZ twins (22.1 (2.7) years; range: 18–26 years, 7 male pairs, 7 female pairs). Fifteen of thirty-two monozygotic and ten of fourteen dizygotic twin pairs were living together at the time of the examination. The experimental protocol was approved by the Ethics Committee for Human Experiments of the Bayerische Landesärztekammer (Munich, Germany) and written informed consent was obtained from the participants. The subjects spent three consecutive nights in our sleep laboratory, where the first night served for adaptation and exclusion of sleep disturbances. Almost all twin partners were recorded at the same time. EEG data of the second and third recording night were used for spindle analysis.

### EEG Recording

All polysomnographic recordings (Comlab 32 Digital Sleep Lab, Brainlab V 3.3 Software, Schwarzer GmbH, Munich, Germany) were performed according to the international 10–20 electrode system (high-pass filter at 0.53 Hz, low-pass filter at 70 Hz, sampling rate of 250 Hz). Electrooculograpic (EOG) montage was done according to Rechtschaffen and Kales (1968). We recorded nap validation samples and memory samples with C3A2 and C4A1 EEG electrodes, whereas twin samples were recorded using 10 EEG electrodes: Fp1A2, Fp2A1, F3A2, F4A1, C3A2, C4A1, P3A2, P4A1, O1A2 and O2A1. Professional scorers scored sleep stages in 30 s epochs according to the standard guidelines (Rechtschaffen and Kales, 1968). Recordings of the twin partners were scored by the same rater.

### SIESTA Algorithm

The SIESTA algorithm was described in detail by Anderer et al. (2005). This solution was created using a large database of visually detected sleep spindles (SIESTA database). Briefly, spindle criteria were based on sleep spindle characteristics from the database: length from 0.3–2 s, minimal peak-to-peak amplitude at least 12 μV and frequency from 11–16 Hz. Authors introduced these criteria to a spindle detector described by Schimicek et al. (1994; briefly described in the introduction). Localized spindle candidates fulfilling minimal criteria were further evaluated with a classifier trained on the SIESTA database. Spindle classification was based on linear discriminant analysis and as an input used spindle duration and mean amplitudes in four frequency bands: spindle, theta, alpha and fast beta. The outcome of each spindle evaluation was a discriminant score, and the SIESTA detector offers three detection thresholds for discriminant scores. If a user chooses the lowest threshold, the algorithm accepts all “*possible*” spindles. This threshold resulted in 90% detection sensitivity in the SIESTA database. The middle threshold accepts all “*probable*” spindles. This threshold maximized the agreement with human scorers in the SIESTA database by maximizing the sum of sensitivity and specificity. The highest threshold accepts only “*certain*” spindles. This threshold resulted in detection specificity above 97% in the SIESTA database.

For both data sets, validation and sleep-related memory consolidation sample, we report results of SIESTA analysis performed with middle detection threshold (“*probable*” patterns), which seems to balance detection sensitivity and specificity.

### Statistical Analysis

#### Algorithm Validation

Our validation data set consisted of detailed information about the exact placement of each detected sleep spindle for both SIESTA analysis and visual scoring. We compared spindles marked in time using 0.1 s windows to obtain the number of true positives (TP), true negatives (TN), false positives (FP) and false negatives (FN). The problem related to statistical analysis of spindle detection agreement is the fact that the majority of EEG signal usually does not contain spindles, which inflates strongly TN and mildly FP. Due to class imbalance, we report results of multiple agreement measures. First, we calculated sensitivity (TP/[TP + FN]), specificity (TN/[TN + FP]) and precision (TP/[TP + FP]). These measures are commonly used, so we report them for the sake of comparison with other published spindle detectors. However, due to the aforementioned bias, specificity outcomes tend to be strongly overestimated, and precision mildly underestimated. We also calculated the general scoring agreement using measures which should correct for the bias towards long fragments of signal, where there are no sleep spindles: adjusted geometric-mean (Batuwita and Palade, 2012), Matthews correlation coefficient and Cohen’s kappa coefficient (equations can be found in Supplementary Material). Adjusted geometric-mean was developed to measure the agreement in imbalanced datasets, where the positive data examples are largely outnumbered by the negative data examples. It adjusts the impact of sensitivity and specificity according to the observed size differences between classes. Matthews correlation coefficient is a geometric mean corrected for chance agreement. It actually returns the same values as Pearson correlation of spindles marked in time between two scorers. Kappa takes the observed agreement and corrects it for a putative chance agreement. There are several benchmarks characterizing agreement based on Cohen’s kappa values. According to Landis and Koch (1977) kappa values from 0–0.2 have been termed *slight*, between 0.21 and 0.40 *fair*, between 0.41 and 0.60 *moderate*, between 0.61 and 0.80 *substantial*, and between 0.81–1 as *almost perfect* agreement. In addition, we used Pearson’s correlation to obtain subjects-wise spindle density agreement.

Human scorers marked sleep spindles only in stage 2 sleep, since in SWS it is much more difficult to visually detect spindles intermingled into delta waves. For this reason the agreement comparison for stage 2 sleep included visual scoring and automatic algorithms, whereas for SWS we compared only our detector and SIESTA algorithm. We analyzed the agreement of sleep spindles scored in the C3A2 EEG channel.

#### Sleep-Related Memory Consolidation

For the sleep-related memory consolidation data, we had only a general outcome from SIESTA spindle analysis about each subject, including average spindle density, amplitude and duration in sleep stage 2 and SWS. We used Pearson’s correlation to obtain subjects-wise spindle density agreement between algorithms as well as between spindle density and declarative memory performance. We analyzed sleep spindle activity in the C4A1 EEG channel.

#### Twin Study—Genetic Variance Analysis

We investigated MZ and DZ twins in order to separate the variance of sleep variables into environmental and genetic components according to Christian et al. (1974, 1987). Briefly, there are two independent estimates of genetic variance: the within-twin pair estimate (GWT), and the combined within- plus among-twin pair component estimate (GCT). GWT depends only on mean squares (MS) for within-pair variation, whereas GCT depends on MS of both within- and among-twin pair variation. A test of equality of variances (*F*’ test) for MZ and DZ twins determines the selection of genetic variance estimate. We used the GCT test when MZ and DZ variances were not equal (the null hypothesis of equal variances was tested using alpha = 0.2, as suggested by the authors). In the other case the GWT test was used. As a prerequisite for the analysis, each studied variable had to fulfill the assumptions of normal distribution (measured by a non-significant goodness-of-fit by the Kolmogorov-Smirnov test) in both twin samples and equal means between twin samples (*t*-test). The significantly unequal means between MZ and DZ twin samples indicate that the investigated variable could be associated with the type of twins being studied. In this case the estimation of genetic variance would be biased. Therefore, if there was an evidence for significantly unequal means between MZ and DZ twin samples, the GVA was not performed. The influence of covariates (age, sex and cohabitation) was analyzed by MANCOVA. Prerequisites were considered to be violated, if the appropriate test showed a significant result at the 5% level. GVA was performed on the mean results of two recording nights. We include a more detailed description of GVA in the Supplementary Material.

We estimated the genetic influence on the most basic parameters describing sleep spindle activity during the whole night: the absolute number of spindles, spindle density (average number of spindles per 30 s epoch), length, amplitude and mean frequency. In order to minimize the effects of possible covariates, we selected a subgroup of MZ twins closely matched for age, gender and cohabitation to DZ twins. GVA for matched MZ and DZ samples can be found in the Supplementary Material. We analyzed sleep spindle activity in left hemisphere. In the results section we present GVA from F3A2 and P3A2 EEG derivations, analysis from Fp1A2 and C3A2 channels can be found in the Supplementary Material.

#### Twin Study—ICC Analysis

We illustrate differences between within-twin pair resemblance and night-to-night stability with intraclass correlation coefficients (ICCs). In order to reveal the strength of observed ICC results, we applied bootstrapping analysis as well as providing the interpretation of computed correlations proposed by Landis and Koch (1977). To obtain levels of statistical significance for ICC results we applied bootstrapping analysis similarly to Tarokh et al. (2011). Each sample was recreated by choosing subject values randomly with repetitions up to the same number as in the original set. For each bootstrapped sample ICC was computed. Only positive ICC values of bootstrapped samples were accepted. Bootstrapping was continued until 1000 positive ICC values were reached. For each investigated parameter we present ICC results of original sample together with the 1/100th top percentile (congruent with significance level *P* = 0.01) and median (congruent to positive ICC values obtained by chance) value of bootstrapped data. Bootstrapping was performed separately for each investigated sample. The sample for within-pair similarity estimation consisted of 64 values in MZ twins (32 twin pairs, 2 values for each pair) and 28 values in DZ twins (32 twin pairs, 2 values for each pair). The sample for stability estimation between consecutive nights consisted of 128 values in the MZ set (32 twin pairs, 2 subjects in each pair, 2 values for each subject) and 56 values in the DZ set (14 twin pairs, 2 subjects in each pair, 2 values for each subject). The smaller the sample size, the easier it is to obtain high ICC by chance. For this reason, bootstrapped ICC values are higher for samples with smaller sizes. According to Landis and Koch (1977), ranges of ICC values were designated as being in *slight* agreement (from 0–0.2), *fair* agreement (from 0.21–0.40), *moderate* agreement (from 0.41–0.60), *substantial* agreement (from 0.61–0.80), and *almost perfect* agreement (from 0.81–1). ICCs estimating within-pair resemblance were performed on mean results of two recording nights.

#### Automatic Sleep Spindle Detection: Description of the Algorithm

**Figure 1:**
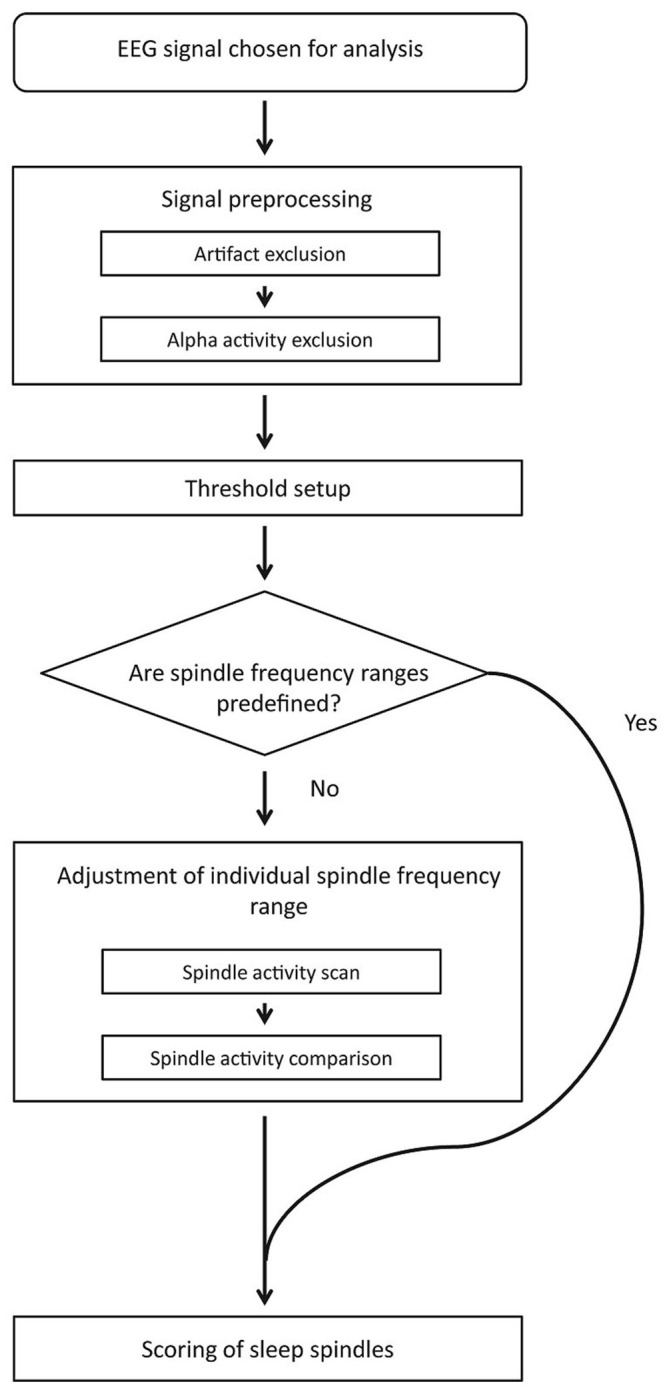
**Algorithm detection scheme**.

Figure 1 depicts the block diagram of spindle detection procedure. First, our method rejects artifacts and strong alpha activities. The signal chosen for spindle detection without excluded fragments is used in further analysis. The detection threshold is then set separately for each channel. If slow and fast spindle frequency boundaries are not predefined, an automatic adjustment procedure sets them individually for each subject using frontal and parietal EEG channels. When spindle frequency boundaries and detection threshold are set, the algorithm scores sleep spindles.

#### Preprocessing Before Spindle Detection

To decrease the computation load, algorithm re-samples the signal to 100 Hz. Therefore, the algorithm resolution is 0.01 s. The first part of the algorithm checks the properties of the signal and rejects periods of signal with high muscle contamination as well as segments dominated by alpha activity.

##### Artifact exclusion

In order to identify fragments with high frequency muscle artifacts, the EEG signal was band-pass filtered (FIR filter; −3 dB at 19.8 and 45.5 Hz). The standard deviation of the signal was computed over a 1 s sliding window (step: 0.5 s) and if it exceeded 5.75 μV, a window of 7 s (fragment in which the threshold was exceeded ± 3 s) was excluded from further analysis.

##### Exclusion of segments with strong alpha activity

Alpha activity is present in the EEG signal mostly during wake when the eyes are closed, but can also be present in EEG during shallow sleep, after arousals and during REM sleep. The shape and frequency of alpha waves (long waxing and waning bursts of activity in the range of 8–12 Hz) is similar to sleep spindles and therefore may lead to false spindle detection. To exclude EEG fragments with probable strings of alpha waves, alpha activity was compared with delta activity on long signal fragments. First, the signal was high-pass filtered (FIR filter; −3 dB at 1.4 Hz). Then, we computed the amplitude spectrum [Fast Fourier Transform (FFT) performed on a 4 s Hanning window; step: 1 s] and for each second mean amplitude was stored for 2–4 (delta) Hz and 8–12 (alpha) Hz frequency ranges. Alpha and delta activity were compared in a 15 s sliding window (step: 1 s). Fifteen values for both alpha and delta activity were weighted using a Hanning window and then averaged, resulting in alpha_activity_ and delta_activity_. Due to the Hanning window, central values in an analyzed fragment had the strongest influence on the outcome. A 15 s fragment was excluded from further analysis if alpha_activity_ was higher than 1.1×delta_activity_.

The reasoning behind our preprocessing methods is described more in detail in the Supplementary Material.

#### Threshold Setup

The threshold was computed using exactly the signal chosen for spindle detection, without fragments excluded due to artifacts or strong alpha activity. Our aim was to obtain a basic threshold (BT) value close to signal background activity. We therefore firstly focused on the 6–18 Hz frequency range, since frequencies below 6 and above 18 Hz are strongly influenced by sleep quality (amount and strength of delta waves), and could be strongly influenced by artifacts (for example muscle contamination). The signal was band-pass filtered (FIR filter; −3 dB at 5.5 and 18.2 Hz) and amplitude spectra were computed (FFT; 2 s sliding window; step: 2 s). Second, amplitude spectra were logarithm transformed (base 10). Due to this transformation, all peaks in activity had a lower influence on the final outcome. Third, the median over all amplitude spectra was computed in order to obtain the background activity for each frequency bin, since the median should be less influenced by temporary events than a mean. BT was set as a mean background activity in the 6–18 Hz range. Two thresholds were defined for spindle detection: minimum spindle activity threshold (SA) and minimum spindle peak threshold (SP). SA was set as 55 times BT, while SP was set as 80 times BT.

#### Detection of Spindle Events

**Figure 2:**
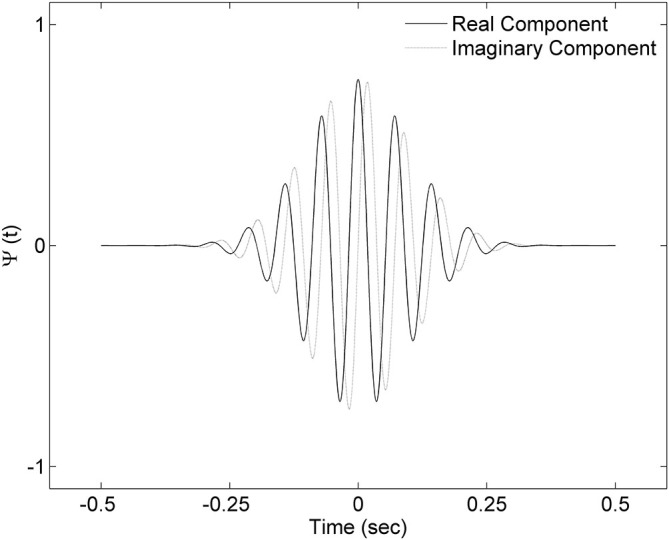
**Complex Morlet wavelet with central frequency *f*_0_ = 2 used in the analysis.** Presented wavelet corresponds to 14 Hz frequency.

In order to detect spindle events, we applied the continuous wavelet transform (CWT) to the signal. As a mother wavelet, we used the complex Morlet wavelet which follows the equation:

$$\psi \left(\frac{t-b}{a}\right)=\frac{1}{\pi^{1/4}}e^{i2\pi f_{0}[(t-b)/a]}e^{-\;{\left[(t-b)/a\right]}^{2}}$$

where *t* is time, *a* is scale parameter so the mother wavelet can be dilated according to the frequency of interest and shifted across the signal using the location parameter *b*. Central frequency *f*_0_ influences the frequency of a complex sinusoid inside the wavelet envelope. For our mother wavelet we chose central frequency *f*_0_ = 2, since it closely resembles a spindle shape. The example of the mother wavelet is shown on Figure 2. A spindle was identified, if the outcome of CWT exceeded SA by a period of at least half a second, and SP at least once. The spindle was marked over the signal fragment, where CWT exceeded SA.

#### Adjustment of Individual Spindle Frequency Range

**Figure 3:**
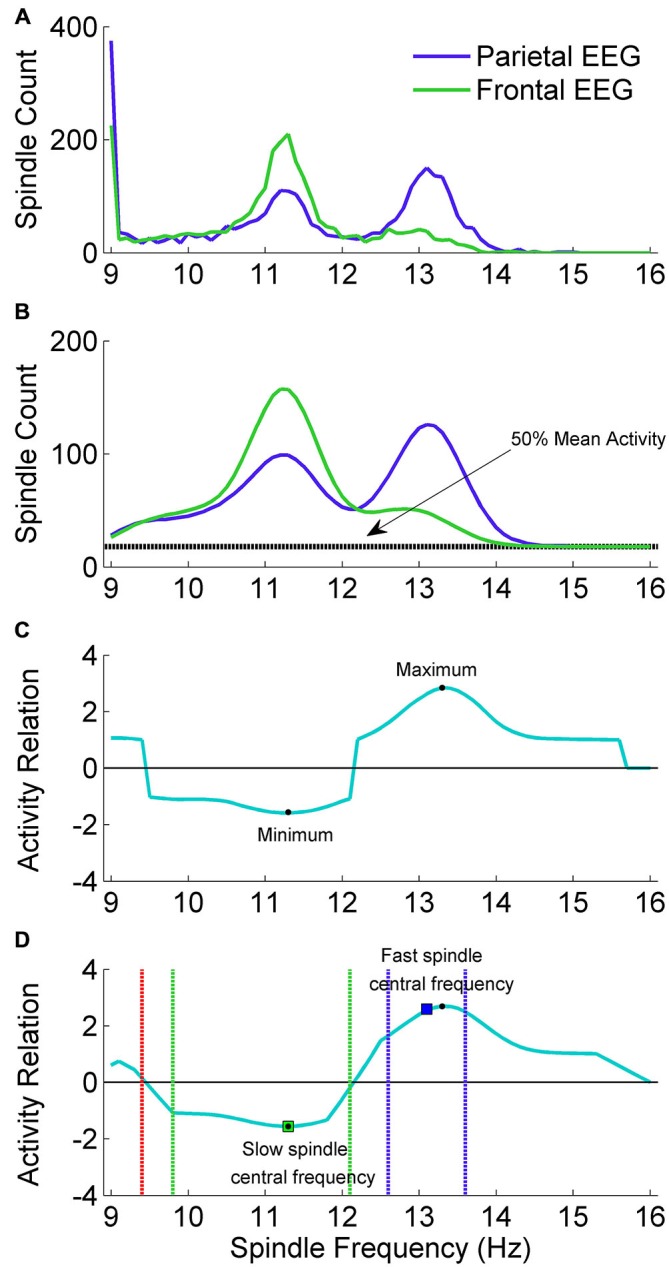
**The adjustment scheme of individual spindle frequency range. (A)** The outcome of spindle activity scan which resulted in two vectors of spindle activity over frequency range separately for frontal channel F3A2 (vecslow: green color) and parietal channel P3A2 (vecfast: blue color). **(B)** In both activity vectors the value in 9 Hz was set to zero, vectors were smoothed and 50% of mean spindle activity (dashed black line) was added to both of them. **(C)** Vector (vec_rel_) showing a relation of spindle activity between frontal EEG and parietal EEG, computed according to “Spindle Activity Comparison” Section. **(D)** Smoothed vec_rel_. First, algorithm localized minimum and maximum (black dots). Localized minimum in vec_rel_ was set as slow spindle central frequency (green square). Localized maximum in vec_rel_ was a starting point to estimate fast spindle frequency ranges using vec_fast_. Local maximum in vec_fast_ was set as fast spindle central frequency (blue square). Ranges of fast (dashed blue lines) and slow (dashed green lines) spindle frequency were estimated according to “Spindle Activity Comparison” Section. First frequency bin below slow spindle range in which spindle activity was higher in the parietal channel was set as frequency in which slow spindles are unlikely (stop_detect_: red dashed line).

Slow spindle activity is more prominent in frontal EEG channels and fast spindle activity is more prominent in parietal channels. In order to localize individual ranges of fast and slow spindle frequency, our algorithm scanned spindle events activity in the 9–16 Hz frequency range and compared the frequency distribution of spindles detected in frontal and parietal EEG channels. Individual spindle frequency range was computed using exactly the signal chosen for spindle detection, without fragments excluded due to artifacts or strong alpha activity. The example of spindle frequency estimation is illustrated in Figure 3.

##### Spindle activity scan

We performed a spindle activity scan using frontal EEG channel F3A2 and parietal channel P3A2. For each channel, CWT was computed with wavelets corresponding to the 9–16 Hz frequency range (step: 0.1 Hz). For the CWT outcome in each frequency bin (CWT_bin_), fragments fulfilling spindle criteria were marked (outcome of CWT_bin_ exceeded SA by a period of at least half a second, and SP at least once). For each frequency bin, every marked fragment overlapping exactly with the signal section where CWT_bin_ exceeded threshold SA was then investigated. Mean CWT_bin_ over this fragment was computed for each 0.1 Hz frequency bin in the 9–16 Hz range (71 bins). A localized fragment was accepted as a spindle belonging to the currently analyzed frequency bin only if currently analyzed frequency was dominant. That is, only if mean CWT_bin_ over this fragment in this frequency was higher than every other mean CWT_bin_ over this fragment for each other 0.1 Hz frequency bin in the 9–16 Hz range. If other frequency than currently analyzed was identified as dominant, this fragment was rejected. For each 0.1 Hz frequency bin all accepted spindles were summarized and these sums were combined into a vector of spindle activity over frequency range separately for frontal channel F3A2 and parietal channel P3A2. The example outcome of spindle activity scan is illustrated in Figure 3A.

##### Spindle activity comparison

Spindle activities estimated for frontal and parietal EEG signals were compared to find frequency ranges of slow and fast spindles. Slow spindle activity is more prominent in frontal EEG channels and fast spindle activity is more prominent in parietal channels. For this reason, vector with spindle activity data from frontal EEG channel is called vec_slow_ and vector with spindle activity from parietal EEG is called vec_fast_. Since 9 Hz was the lowest frequency bin for which spindle activity scan was performed, frequency of spindles detected using wavelet in 9 Hz frequency was compared only to higher frequencies. Therefore the 9 Hz frequency bin in both spindle activity vectors (vec_slow_ and vec_fast_) included spindle bursts in 9 Hz and possibly below. Sleep spindles in such a low frequencies are unlikely. For this reason, the value in both spindle activity vectors responding to 9 Hz was set to zero. Then a moving average (0.7 Hz window) was applied twice for each vector to smooth the data. The example of preprocessed spindle activity vectors is illustrated in Figure 3B.

The next step was to compute a vector (vec_rel_) showing a relation of spindle activity between vec_slow_ and vec_fast_. First, we calculated a grand mean (mean_act_) over both activity vectors of average spindle activity for all frequency bins. Vec_rel_ was computed according to the following rule:


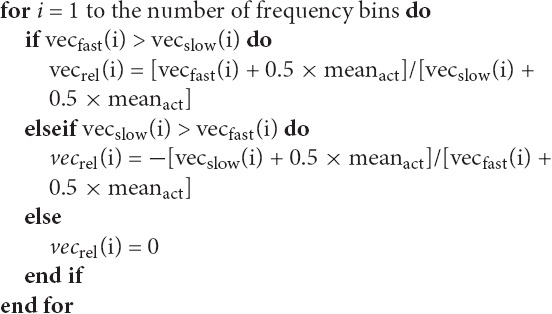


Vec_rel_ is positive when there are more spindles in vec_fast_ and negative when there are more spindles in vec_slow_. 50% of mean_act_ was included to avoid cases when small spindle numbers in vec_slow_ and vec_fast_ produce very high results in vec_rel_. The example of obtained vec_rel_ is illustrated in Figure 3D.

Vec_rel_ was smoothed (moving average, 0.7 Hz window) before localizing slow and fast spindle frequency range. The minimum value in vec_rel_ shows the strongest relative spindle activity in frontal EEG when compared to spindle activity in parietal EEG. Frequency responding to this minimum value was taken as a putative central frequency of slow spindle activity (slow_cntr_). To find a putative central frequency of fast spindle activity (fast_cntr_) algorithm analyzed vec_rel_ in the frequency range between slow_cntr_ and 16 Hz. Frequency responding to the maximum value in vec_rel_ within the slow_cntr_–16 Hz range was taken as a candidate for fast_cntr_.

Fast spindle activity is usually clearly visible in vec_fast_. Therefore, fast_cntr_ was shifted from the maximum in vec_rel_ towards the local maximum in vec_fast_. The range of fast spindle frequency was estimated similarly to method presented by Bódizs et al. (2009): second derivative of vec_fast_ was computed and zero-crossing points encompassing fast_cntr_ were taken as fast spindle frequency ranges.

Frequency ranges of slow spindle activity in vec_slow_ are often difficult to distinguish, so they were estimated using vec_rel_. The higher boundary was extended from slow_cntr_ to the highest frequency below fast spindle frequency range, in which spindle activity was higher in the frontal channel. The lower boundary of slow spindle activity was more difficult to establish, since it is important to avoid classification of alpha waves as sleep spindles. Therefore the lower boundary of slow spindle frequency range was extended cautiously from slow_cntr_ to the first frequency bin in which vec_rel_ value was 40% higher than minimum in slow_cntr_. In addition, the algorithm set a frequency stop_detect_ in which slow spindles are unlikely and should not be detected. Stop_detect_ was set as the highest frequency below slow spindle frequency range, in which spindle activity was higher in the parietal channel. If such a frequency was not present above 9 Hz, stop_detect_ was set at 9 Hz. The example outcome of spindle frequency estimation is illustrated in Figure 3D.

**Figure 4:**
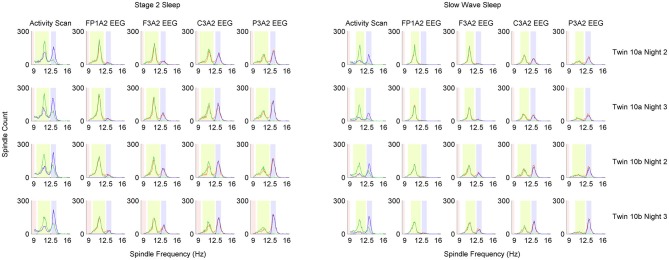
**Distribution of detected sleep spindles in 0.1 Hz frequency bins in monozygotic (MZ) twin pair number 10.** Analysis was performed separately for stage 2 and slow wave sleep (SWS). Each row of plots represents one recording night. Column *Activity Scan* shows the result of pre-analysis performed to localize slow and fast spindle frequency ranges. During *activity scan* spindles were detected in two EEG derivations: parietal channel P3A2 (blue color) and frontal channel F3A2 (green color). Information from *activity scan* was used to set frequency range of fast spindles (light blue color), slow spindles (light green color) and range in which spindles should not be detected anymore (light red color). Localized frequency ranges were used to detect sleep spindles in four EEG derivations, which are presented in distinct columns: *FP1A2*, *F3A2*, *C3A2* and *P3A2*. Blue color depicts sleep spindles detected with wavelets in fast spindle frequency range, green color depicts sleep spindles detected with wavelets in slow spindle frequency range whereas orange color depicts sleep spindles detected with combined slow and fast spindle frequency ranges.

The minimum frequency range was set as at least 0.5 Hz around estimated central frequencies of fast and slow spindles. Spindle activity comparison between vec_slow_ and vec_fast_ was performed only if each vector included at least 30 spindles. Otherwise estimation of spindle detection ranges would have low reliability. If the amount of detected spindles was too low, frequency range was set at 13.1–15 Hz for fast, 11–12.9 Hz for slow spindles and stop_detect_ at 9 Hz. The result of spindle frequency estimation as well as spindle detection with applied individual frequency ranges for twin pair number 10 is illustrated in Figure 4.

We applied automatic individual adjustment of spindle frequency range in the twin sample, since in this data set recordings include multiple EEG derivations along the antero-posterior axis. However, experiments in which sleep spindle analysis is of interest often include recordings with few EEG channels. Our validation sample and sleep-related memory consolidation sample included only central EEG derivations C3A2 and C4A1. Therefore, in our algorithm the user has the option to set the frequency range for slow and fast spindles. For all recordings in the validation sample and sleep memory consolidation sample, we set 11–12.9 Hz as slow and 13.1–16 Hz as fast spindle frequency range.

#### Scoring of Sleep Spindles

In order to score sleep spindles, the algorithm analyzed results of CWT computed with wavelets corresponding to stop_detect_ frequency, slow spindle frequency range (CWT_slow_) and fast spindle frequency range (CWT_fast_). We computed CWT_slow_ in each time point as a maximum CWT value in this time point over slow spindle frequency range. CWT_fast_ was computed the same way. In addition to slow and fast spindles, sleep spindles without distinction between slow and fast ones were scored (all sleep spindles).

##### All sleep spindles

All sleep spindles were detected using the maximum of both CWT_slow_ and CWT_fast_. (CWT_all_). Places fulfilling spindle criteria (according to “Detection of Spindle Events” Section) for CWT_all_ were localized. A marked place was accepted as a sleep spindle, if over this place the mean CWT_all_ was higher than the mean CWT of stop_detect_.

##### Fast sleep spindles only

Fast sleep spindles were detected using CWT_fast_. Places in which CWT_fast_ was continuously higher than CWT_slow_ and spindle criteria for CWT_fast_ were fulfilled (according to “Detection of Spindle Events” Section), were classified as fast sleep spindles.

##### Slow sleep spindles only

Slow sleep spindles were detected using CWT_slow_. The algorithm localized fragments in which CWT_slow_ was continuously higher than CWT_fast_ and spindle criteria for CWT_slow_ were fulfilled (according to “Detection of Spindle Events” Section). A marked fragment was classified as a slow sleep spindle, if over this place the mean CWT_slow_ was higher than the mean CWT of stop_detect_.

**Figure 5:**
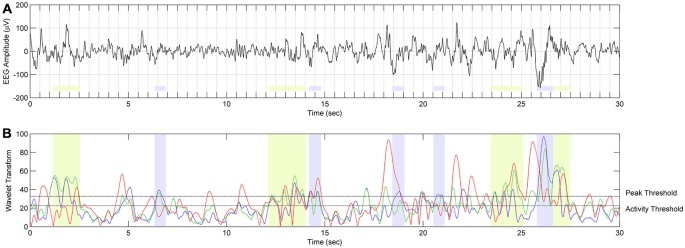
**The scheme of spindle detection. (A)** EEG signal from C3A2 derivation during SWS in twin 10a during night 2 (localization of spindle frequency ranges and overall results of spindle detection in twin 10a are presented in Figure 4). **(B)** The result of continuous wavelet transform (CWT) in time and frequency domain. Red color depicts WT result using wavelet corresponding to 9 Hz frequency. Events with this frequency are not classified as spindles. Green color depicts WT using wavelets corresponding to 10.4–12 Hz frequency range. Events detected in this frequency range are classified as slow spindles (light green color). Blue color depicts WT using wavelets corresponding to 12.5–13.5 Hz frequency range. Events detected in this frequency range are classified as fast spindles (light blue color).

Results of spindle detection in four EEG channels along the antero-posterior axis for twin pair number 10 are illustrated in Figure 4. We included such figures for all analyzed twin pairs in the Supplementary Material. An example of sleep spindle detection on EEG fragment is presented in Figure 5.

Twin data included frontal and parietal EEG channels, therefore we could apply our automatic individual spindle frequency adjustment and report results from fast and slow spindle detection. In contrast, the validation set as well as memory consolidation data included only central electrodes. For this reason, in these two datasets we used fixed spindle frequency ranges and analyzed results of all sleep spindles detected, without distinction between slow and fast ones.

#### Computation of Sleep Spindle Amplitude and Frequency

In order to estimate sleep spindle amplitude and dominant frequency, the signal was first band-pass filtered (FIR filter; −3 dB at 8.7 and 18.5 Hz). Then, a Hanning window was applied to exact a fragment with a marked spindle, and an amplitude spectrum was computed similarly to Huupponen et al. (2006): the fragment was zero-padded to 10 s window and FFT was computed resulting in frequency resolution of 0.1 Hz. The maximum peak in the amplitude spectrum was taken as spindle amplitude and frequency.

#### Average Spindle Detection Time

The time required to perform the spindle detection for the whole night EEG recording (around 8 h of sleep) in four EEG channels, with spindle detection ranges individually adjusted using one frontal and one parietal channel, was around 4 min 15 s. When spindle detection ranges were fixed, the CWT algorithm required around 2 min to perform spindle detection in four EEG channels. We performed the analysis using an Intel i5-4310M processor (2.7 GHz, 3 MB).

## Results

### Algorithm Validation

**Figure 6:**
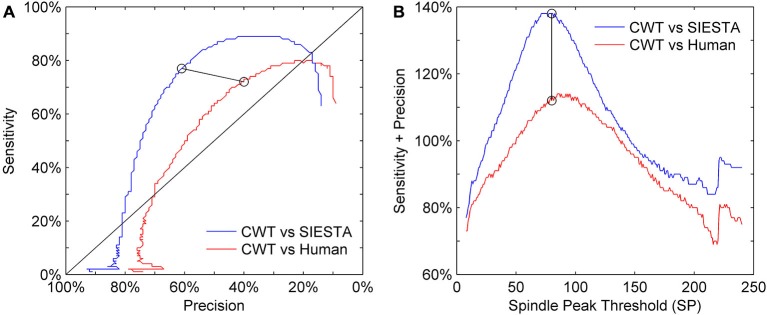
**Sensitivity-precision plot showing how these two measures depend on spindle detection thresholds.** Sleep spindles were scored in C3A2 EEG channel. **(A)** ROC-like plot of sensitivity vs. precision, **(B)** the sum of sensitivity and precision according to detection thresholds variety. We had two detection thresholds in our algorithm: spindle peak threshold (SP) set as 80 times basic threshold (BT) and spindle activity threshold (SA) set as 55 times BT (calculation of BT is described in “Threshold Setup” Section). We illustrate how performance changes according to SP, where *y* axis shows multiplication rate of BT used to obtain SP, but for each iteration values of both thresholds were changed together to always keep the same ratio between them (SP = 1.45 × SA). Black circles connected with black line mark sensitivity and precision obtained for thresholds chosen for our algorithm.

Our choice of the mother wavelet as well as detection thresholds ratio (spindle activity threshold SA and minimum spindle peak threshold SP) was based on visual observation of sleep spindles and their CWT transforms. We set the actual values of detection thresholds on a level which matched detection sensitivity presented by the SIESTA algorithm. Figure 6 shows the precision and sensitivity results from a validation dataset of the CWT detector vs. human and vs. SIESTA algorithm using a range of detection threshold levels. We always changed both thresholds percentage-wise, to keep their ratio intact (SP = 1.45 × SA). Results show that a similar amount of detected spindles between our algorithm and SIESTA detector resulted in the highest possible combination of sensitivity and precision. Also, in order to maximize the agreement with a human scorer, we would need to raise the thresholds by 10%. However, the agreement of our detector with a human would be still much lower than the agreement between two machines.

**Figure 7:**
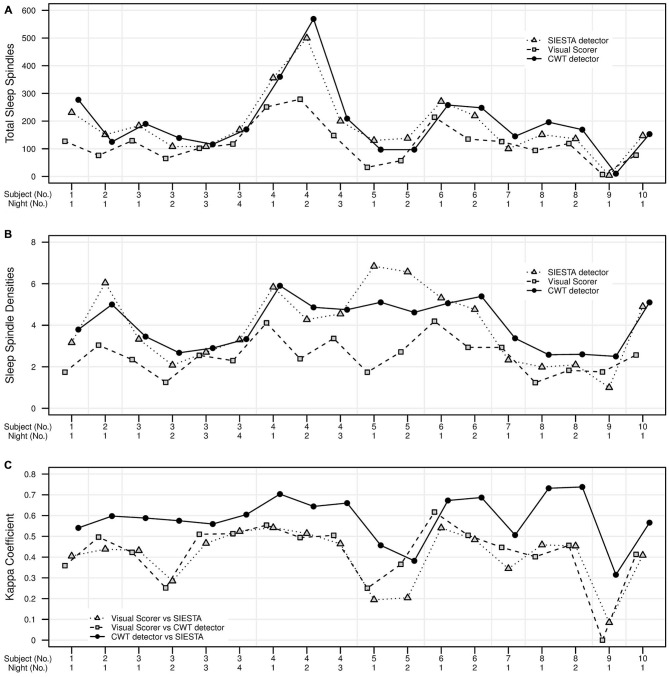
**Validation set of 18 nap EEG recordings.** Agreement in sleep spindle detection during stage 2 in C3A2 EEG derivation between our algorithm, human visual scorer and SIESTA automatic spindle detector. On *y* axis there are presented: **(A)** total number of detected sleep spindles for each recording, **(B)** spindle density for each recording, **(C)** the kappa coefficient of scorers agreement for each recording. Subject id and number of nap recording are presented on *x* axis.

The agreement between our algorithm, human scorer and SIESTA algorithm on the validation data set is illustrated in Figure 7 and summarized in Table 1. During stage 2 sleep, mean spindle density was 4.0 for our algorithm, 3.95 for SIESTA detector and 2.5 for human scorer. The subject-wise correlation of spindle density between our detector and SIESTA was *r* = 0.86. The correlation of spindle density between our detector and human scoring was *r* = 0.73, whereas the correlation between the SIESTA detector and human scorer was *r* = 0.55. Due to the fact that amounts of NREM sleep stages differed significantly between recordings, we computed our agreement measures using weighted averages, where weight for each recording was its number of investigated sleep epochs divided by the total number of investigated sleep epochs from all recordings. The agreement between our detector and SIESTA algorithm measured with kappa ranged from 0.31–0.74, with weighted average kappa of 0.62 (sensitivity: 0.77; specificity: 0.93; precision: 0.61). The kappa between our detector and human scorer ranged from 0–0.62 with weighted average of 0.45 (sensitivity: 0.72; specificity: 0.90; precision: 0.40) and kappa between the SIESTA detector and human scorer ranged from 0.08–0.54 with weighted average of 0.44 (sensitivity: 0.62, specificity: 0.92, precision: 0.43). We observed very similar results when using Matthews correlation, with high agreement between automatic detectors when compared to the agreement between algorithms and human scorer. Discrepancies between machines and human were smaller when the agreement was measured using adjusted geometric mean. The reason for that is the human scorer marked the smallest amount of spindles in the signal, resulting in the strongest imbalance between classes. As a result, specificity in this case had the strongest influence on the outcome of the adjusted geometric mean (equations can be found in the Supplementary Material).

**Table 1:** **Spindle detection agreement between our CWT detector, SIESTA algorithm and human scorer**.

Agreement measure	CWT vs. SIESTA		CWT vs. Human	SIESTA vs. Human
	Stage 2	SWS	Stage 2	Stage 2
Sensitivity	0.77	0.64	0.72	0.62
Specificity	0.93	0.94	0.90	0.92
Precision	0.61	0.66	0.40	0.43
Kappa	0.62	0.56	0.45	0.44
Adjusted geometric mean	0.88	0.85	0.85	0.83
Matthews correlation	0.63	0.57	0.48	0.46
Spindle density correlation	0.86	0.80	0.73	0.55

*Sleep spindles were scored in the C3A2 EEG channel. CWT, continuous wavelet transform; SWS, slow wave sleep*.

According to published benchmarks for kappa coefficient (Landis and Koch, 1977) the agreement between our algorithm and SIESTA detector was *fair* for two naps, *moderate* for nine naps and *substantial* for seven naps. The agreement between our algorithm and human scorer was *fair* for five naps, *moderate* for 11 naps, *substantial* for one nap and in the one case, there was no agreement between our algorithm and human scorer. The agreement between human scorer and SIESTA detector was *sligh*t for three naps *fair* for four naps and *moderate* for 11 naps.

SWS was not present in two nap recordings, so the validation set consisted of 16 naps from 9 subjects. During SWS, mean spindle density was 4.03 for our algorithm and 4.56 for SIESTA detector. The subject-wise correlation of spindle density between our detector and SIESTA was *r* = 0.80. According to kappa coefficient, the agreement between our detector and SIESTA algorithm ranged from 0.35–0.86, with weighted average kappa of 0.56 (sensitivity: 0.64; specificity: 0.94; precision: 0.66). The agreement between our algorithm and SIESTA detector was *fair* for three naps, *moderate* for four naps, *substantial* for eight naps and *almost perfect* for one nap.

**Figure 8:**
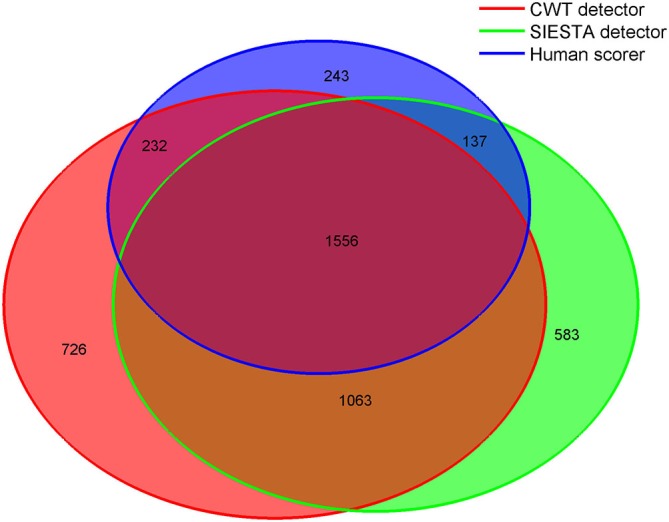
**Venn diagram showing in numbers of detected spindles, how spindles detected by each scorer overlapped with spindles detected by other scorers.** Sleep spindles were detected during stage 2 in C3A2 EEG derivation.

Since the agreement between scorers was mostly *moderate*, we tried to reveal the reasons for disagreement between scorers by investigating in detail the group of consensus spindles, which were marked by all scorers, as well as distinct groups of spindles marked by only one scorer. To assume that scorers agreed on a spindle, at least 0.3 s consecutive marked fragment had to overlap. We chose this length since 0.3 s was the shortest spindle length marked by scorers. We analyzed spindles detected during stage 2 sleep. Figure 8 shows overlap between scorers in marked spindles. All spindles were measured as described in “Computation of Sleep Spindle Amplitude and Frequency” Section . Amplitudes in other frequency ranges were computed using similar technique, however without pre-filtering of the signal. Results are presented in Table 2.

**Table 2:** **Characteristics of sleep spindles detected by all scorers (consensus) and of spindles detected only by a single scorer in stage 2 sleep**.

	Consensus (*N* = 1556) Mean *(SD)*	Only CWT (*N* = 726) Mean *(SD)*	Only SIESTA (*N* = 583) Mean *(SD)*	Only Human (*N* = 243) Mean *(SD)*
Spindle	12.51 (3.46)	8.54 (2.30)	9.24 (3.54)	7.92 (4.00)
amplitude (μV)^a^
Spindle	13.75 (1.15)	11.94 (1.74)	13.10 (1.23)	12.05 (2.70)
frequency (Hz)
Spindle	0.88 (0.33)	0.81 (0.42)	0.74 (0.41)	0.99 (0.47)
length (s)
Delta (μV)^b^	6.74 (4.16)	10.22 (7.44)	7.26 (4.74)	7.45 (6.38)
Theta (μV)^b^	4.61 (2.30)	6.56 (4.22)	4.82 (2.95)	5.95 (4.93)
Alpha (μV)^b^	3.80 (1.94)	5.10 (2.33)	4.34 (3.18)	6.27 (3.98)
Spindle to	2.56 (1.15)	1.22 (0.58)	1.73 (0.72)	1.22 (0.64)
background ratio^c^

*Sleep spindles were scored in the C3A2 EEG channel. ^a^Computed from amplitude spectrum as described in “Computation of Sleep Spindle Amplitude and Frequency” Section. ^b^Mean amplitude in chosen frequency from amplitude spectrum (delta: 2–4.5 Hz, theta: 4.6–7.5 Hz, alpha: 7.6–11 Hz). ^c^Spindle amplitude divided by mean amplitude in 2–11 Hz background frequency computed from amplitude spectrum*.

Consensus spindles could be characterized as the ones with high amplitude (12.51 μV in amplitude spectrum), high frequency (clearly above 13 Hz) and strong activity when compared to the background. Spindles marked only by a single scorer, conversely, had significantly lower amplitudes, frequencies and spindle to background activity ratio. Our CWT detector marked the highest number of spindles not scored by the others (*N* = 726). It was 20% of all spindles marked by our algorithm. Spindles detected only by our detector had the lowest average frequency (11.94 Hz) and the highest activity in delta and theta frequency ranges. Only 11% of spindles marked by the human scorer were not detected by any automatic algorithm. The average frequency of these spindles was close to the ones marked only by the CWT detector (12.05 Hz). Furthermore, spindles marked only by the human scorer had the lowest amplitude in the amplitude spectrum when compared to automatic detectors (7.92 μV), and were the longest. It means that they often in those cases marked longer fragments than the actual spindle activity. The SIESTA algorithm marked 17% of spindles which were not detected by others. Spindles marked only by the SIESTA algorithm had high average frequency (above 13 Hz) as well as a relatively high amplitude and high activity when compared to the background. These spindles were the ones that on average resembled consensus spindles the most, so the question was: why they were not marked by both the human scorer and the CWT detector? The most important reason was that these spindles were on average the shortest (0.74 s). Spindles with this length should be detected, but 24.7% of spindles detected only by the SIESTA algorithm were shorter than half a second. According to our rules, spindles shorter than 0.5 s were not detected. Furthermore, the shortest spindles marked by the SIESTA algorithm also had the highest amplitudes. There was a moderately strong negative correlation, in spindles detected only by the SIESTA detector, between spindle length and amplitude (*r* = −0.53 compared to *r* = −0.24 in spindles detected only by CWT detector). Due to the fact that many spindles marked by SIESTA were short and therefore could be missed, we analyzed just spindles whose length was at least 0.7 s and which were detected just by our algorithm or by the SIESTA detector. Results are presented in Table 3.

**Table 3:** **Characteristics of sleep spindles detected only by the CWT detector and the SIESTA detector, whose length was at least 0.7 s in stage 2 sleep**.

	Only CWT (*N* = 398) Mean *(SD)*	Only SIESTA (*N* = 250) Mean *(SD)*
Spindle amplitude (μV)^a^	8.21 (2.44)	7.80 (3.41)
Spindle frequency (Hz)	11.90 (1.77)	13.02 (1.41)
Spindle length (s)	1.01 (0.48)	1.10 (0.38)
Delta (μV)^b^	9.91 (7.20)	6.28 (3.83)
Theta (μV)^b^	6.13 (3.84)	4.02 (2.12)
Alpha (μV)^b^	4.74 (2.21)	3.63 (2.45)
Spindle to background ratio^c^	1.23 (0.60)	1.73 (0.72)

*Sleep spindles were scored in the C3A2 EEG channel. ^a^Computed from amplitude spectrum as described in “Computation of Sleep Spindle Amplitude and Frequency” Section. ^b^Mean amplitude in chosen frequency from amplitude spectrum (delta: 2–4.5 Hz, theta: 4.6–7.5 Hz, alpha: 7.6–11 Hz). ^c^Spindle amplitude divided by mean amplitude in 2–11 Hz background frequency computed from amplitude spectrum*.

Characteristics of “*long*” sleep spindles detected only by our CWT detector (average spindle amplitude, frequency and background activity) were very similar when compared to all spindles marked only by our algorithm. In “*long*” sleep spindles detected only by the SIESTA algorithm we observed a 15% drop in spindle amplitude, whereas their average frequency remained high and the ratio of their activity to the background remained the same, when compared to all spindles marked only by the SIESTA detector. We conclude that spindles marked only by our algorithm were slower and/or intermingled into other frequencies while spindles marked by the SIESTA detector were either short or had too low an amplitude for other scorers. Spindles marked only by the human scorer were few, characterized by slower frequency and a length longer than the actual spindle activity.

We investigated the performance of our CWT detector using the validation set, which consisted of recordings with only central derivations available. For this reason, we used fixed spindle detection frequency ranges and we did not distinguish between slow and fast spindles, but we analyzed all sleep spindles only. Unfortunately, we could not directly evaluate the performance of the CWT detector with individually adjusted spindle frequency ranges vs. other scorers. To get the impression how adjusted frequency ranges would affect the detection, we compared the agreement of the CWT detector with itself when using fixed spindle frequency ranges vs. individually adjusted spindle frequency ranges. We analyzed the second recording night of our twin data. Our results include pooled detection agreement from Fp1A2, F3A2, C3A2, and P3A2 channels. Results are presented in Table 4.

**Table 4:** **Twin set, night 2**.

Agreement measure	Slow spindles		Fast spindles		All spindles	
	Stage 2	SWS	Stage 2	SWS	Stage 2	SWS
Sensitivity	0.82	0.66	0.64	0.52	0.90	0.74
Specificity	0.97	0.98	0.99	0.99	0.99	0.99
Precision	0.67	0.76	0.80	0.78	0.92	0.95
Kappa	0.69	0.61	0.64	0.53	0.89	0.77
Adjusted geometric mean	0.93	0.87	0.87	0.82	0.96	0.91
Matthews correlation	0.70	0.64	0.67	0.58	0.89	0.80
Spindle density correlation	0.74	0.73	0.72	0.71	0.93	0.74

*Spindle detection agreement between our CWT detector with fixed frequency ranges (slow spindle: 11–12.9 Hz, fast spindle: 13.1–16 Hz) compared to the same detector with individually adjusted spindle frequency ranges. Agreement was calculated from pooled channels Fp1A2, F3A2, C3A2, and P3A2. CWT, continuous wavelet transform; SWS, slow wave sleep*.

The agreement was higher for stage 2 sleep when compared to SWS. The reason was that the algorithm with adjustable frequency ranges detected significantly more spindles during SWS when compared to fixed frequency ranges. The agreement was also high when we considered all sleep spindles together. Mean all spindle density was 4.02 during stage 2 and 4.39 during SWS for algorithm with adjustable frequency ranges compared to 3.96 during stage 2 and 3.47 during SWS for algorithm with fixed frequency ranges. During stage 2 the agreement was *almost perfect*, however during SWS it dropped to *substantial*. The agreement dropped significantly when the CWT detector made a distinction between slow and fast spindles. Mean slow spindle density was 2.05 during stage 2 and 3.15 during SWS for the algorithm with adjustable frequency ranges compared to 2.46 during stage 2 and 2.51 during SWS for the algorithm with fixed frequency ranges. The agreement during both, stage 2 sleep and SWS was *substantial*. Mean fast spindle density was 1.64 during stage 2 and 0.88 during SWS for algorithm with adjustable frequency ranges compared to 1.22 during stage 2 and 0.58 during SWS for algorithm with fixed frequency ranges. During stage 2 the agreement was *substantial* and during SWS it dropped to *moderate*.

### Sleep-Related Memory Consolidation Data

**Figure 9:**
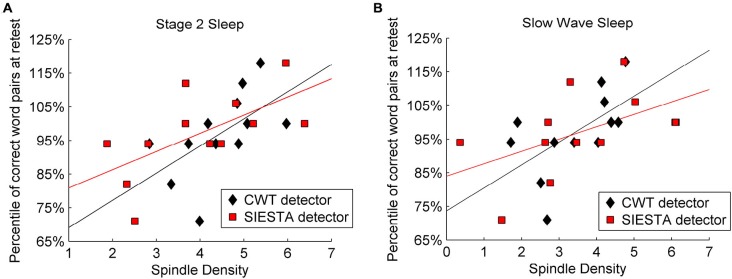
**Relation between declarative memory performance and spindle density computed by two algorithms: SIESTA spindle detector and CWT detector during (A) stage 2 sleep and (B) SWS.** Sleep spindles were detected in C4A1 EEG derivation.

Mean sleep spindle density during stage 2 sleep was 4.46 for our algorithm and 4.0 for the SIESTA detector, whereas during SWS it was 3.43 and 3.56, respectively. Sleep spindle analysis performed with the SIESTA detector was already described by Genzel et al. (2009). Results returned by the SIESTA algorithm revealed significant Pearson’s correlation between spindle density and declarative memory consolidation (stage 2 sleep: *r* = 0.627, *P* = 0.015; SWS: *r* = 0.516, *P* = 0.043). Results returned by our algorithm confirmed previous findings in terms of a significant relationship between spindle density and declarative memory consolidation (stage 2 spindles: *r* = 0.579, *P* = 0.024; SWS: *r* = 0.585, *P* = 0.023). Figure 9 shows the relation between memory consolidation and spindle density. The subject-wise correlation of spindle density between our detector and SIESTA was *r* = 0.93 for stage 2 sleep and *r* = 0.80 for SWS.

As for spindle activity (absolute number of spindles per night × mean spindle amplitude × mean spindle duration), Table 5 shows correlations between declarative memory consolidation and spindle parameters included in spindle activity calculations. During stage 2 sleep, spindle activity obtained from the SIESTA detector were significantly related to declarative memory consolidation (*r* = 0.616, *P* = 0.017; Genzel et al., 2009). However, the relationship of declarative memory consolidation and spindle activity computed using our algorithm was only marginally significant (*r* = 0.468, *P* = 0.062). In SWS, spindle activity obtained from both algorithms was in marginal relationship with declarative memory consolidation (our algorithm: *r* = 0.420, *P* = 0.087; SIESTA: *r* = 0.419, *P* = 0.087). The subject-wise correlation of spindle activity between our detector and SIESTA was *r* = 0.94 for stage 2 sleep and *r* = 0.93 for SWS.

**Table 5:** **Pearson’s correlation between declarative memory consolidation and spindle characteristics**.

	CWT detector		SIESTA detector	
	Stage 2	SWS	Stage 2	SWS
Spindle density	0.58	0.59	0.63	0.52
Spindle absolute number	0.45	0.48	0.60	0.49
Spindle amplitude (μV)^a^	0.31	0.07	0.45	0.28
Spindle length (s)	0.22	0.36	0.50	0.28
Spindle activity^b^	0.47	0.42	0.62	0.42

*Sleep spindles were scored in the C4A1 EEG channel. ^a^Computed from amplitude spectrum as described in “Computation of Sleep Spindle Amplitude and Frequency” Section. ^b^Spindle activity: absolute number of spindles per night × mean spindle amplitude × mean spindle duration*.

#### Genetic Influence on Sleep Spindles

Here we report the results of spindle detection with individually adjusted spindle frequency ranges. All estimated frequency ranges for each twin pair can be found in the Supplementary Material (Tables S1–S3 and Figures S1–S46). GVA of sleep spindles detected with fixed spindle frequency ranges are also included in the supplement. We applied individual adjustment of slow and fast spindle frequency ranges separately for stage 2 sleep and SWS. The average frequency of slow spindles detected during stage 2 sleep was 11.43 Hz with inter-subject variability ranging from 10.04–12.37 Hz. During SWS, the average frequency of slow spindles was 10.99 with 9.62–12.27 Hz inter-subject range. The average frequency of fast spindles detected during stage 2 sleep was 13.59 Hz with inter-subject variability ranging 12.30–14.83 Hz. During SWS, the average frequency of fast spindles was 13.55 with 12.26–14.73 Hz inter-subject range.

The criterion of normal distribution was not fulfilled for the average slow spindle length during stage 2 sleep in the F3A2 EEG channel, therefore it was log transformed prior to all analyses. We observed that age, as a covariate, had a marginally significant effect on fast spindle density (higher spindle density in younger subjects), and sex, as a covariate, had a marginally significant effect on slow spindle number (higher spindle number in females). Sample means of averaged over-pairs measures revealed no significant night effects (Supplementary Material, Tables S4, S6, S8 and S10). However, in the F3A2 derivation, we observed significantly higher slow spindle amplitude in DZ twins during stage 2 sleep as well as significantly higher slow spindle absolute number and density in DZ twins during SWS (Supplementary Material, Tables S8 and S10). Therefore, for these three slow spindle parameters GVA was not applicable. In both, stage 2 sleep and SWS, we identified a significant genetic influence on variance of all but one remaining slow spindle parameter. The exception was the average slow spindle frequency in the F3A2 channel during SWS, on which the genetic effect was only marginally significant. Tables 6, 7 depict GVA of sleep spindle parameters during stage 2 sleep and SWS, respectively.

**Table 6:** **Genetic variance analysis, type of estimate applied (GCT: combined among- and within-twin pair component estimate, GWT: within-pair estimate) and intraclass correlation coefficients (ICCs) for spindle parameters in stage 2 sleep**.

Variable	Type	*P*	Analysis	ICC MZ	ICC DZ	ICC MZ cn	ICC DZ cn
*EEG channel: F3A2*
Number of spindles	Slow	<0.001	GWT	0.91 (0.47, 0.12)	0.43 (0.62, 0.18)	0.92 (0.33, 0.09)	0.88 (0.49, 0.14)
	Fast	0.279	GWT	0.75 (0.44, 0.13)	0.49 (0.61, 0.18)	0.85 (0.32, 0.08)	0.92 (0.49, 0.12)
Spindle density	Slow	0.001	GCT	0.94 (0.45, 0.13)	0.24 (0.65, 0.19)	0.94 (0.31, 0.09)	0.91 (0.46, 0.13)
	Fast	0.164	GWT	0.78 (0.48, 0.11)	0.54 (0.67, 0.18)	0.86 (0.32, 0.08)	0.94 (0.50, 0.13)
Spindle length	Slow	0.002	GCT	0.96 (0.45, 0.12)	0.42 (0.65, 0.19)	0.96 (0.32, 0.09)	0.92 (0.48, 0.13)
	Fast	0.030	GWT	0.74 (0.46, 0.12)	0.40 (0.61, 0.17)	0.82 (0.33, 0.08)	0.91 (0.46, 0.13)
Spindle amplitude	Slow*	−	−	0.88 (0.46, 0.12)	0.19 (0.65, 0.18)	0.91 (0.34, 0.09)	0.88 (0.48, 0.13)
	Fast	<0.001	GCT	0.88 (0.45, 0.13)	0.10 (0.62, 0.16)	0.88 (0.32, 0.08)	0.74 (0.50, 0.13)
Spindle frequency	Slow	<0.001	GWT	0.94 (0.43, 0.12)	0.43 (0.62, 0.18)	0.93 (0.32, 0.09)	0.96 (0.48, 0.13)
	Fast	<0.001	GWT	0.93 (0.42, 0.12)	0.67 (0.64, 0.18)	0.96 (0.33, 0.09)	0.96 (0.48, 0.14)
*EEG channel: P3A2*
Number of spindles	Slow	<0.001	GCT	0.96 (0.53, 0.11)	0.22 (0.69, 0.19)	0.93 (0.34, 0.08)	0.88 (0.48, 0.13)
	Fast	0.271	GWT	0.80 (0.45, 0.12)	0.70 (0.68, 0.19)	0.83 (0.34, 0.09)	0.86 (0.46, 0.13)
Spindle density	Slow	<0.001	GCT	0.96 (0.53, 0.11)	0.04 (0.68, 0.18)	0.94 (0.36, 0.08)	0.91 (0.48, 0.13)
	Fast	0.196	GWT	0.80 (0.44, 0.12)	0.67 (0.66, 0.19)	0.85 (0.32, 0.09)	0.88 (0.44, 0.12)
Spindle length	Slow	<0.001	GCT	0.94 (0.52, 0.12)	−0.19 (0.66, 0.19)	0.90 (0.37, 0.08)	0.81 (0.47, 0.13)
	Fast	0.002	GWT	0.78 (0.45, 0.12)	0.48 (0.62, 0.19)	0.89 (0.33, 0.08)	0.92 (0.47, 0.14)
Spindle amplitude	Slow	0.005	GWT	0.84 (0.46, 0.13)	0.50 (0.61, 0.17)	0.88 (0.31, 0.09)	0.79 (0.47, 0.12)
	Fast	0.047	GWT	0.82 (0.48, 0.13)	0.57 (0.67, 0.17)	0.88 (0.33, 0.09)	0.64 (0.48, 0.12)
Spindle frequency	Slow	<0.001	GWT	0.94 (0.44, 0.12)	0.41 (0.64, 0.18)	0.90 (0.34, 0.09)	0.95 (0.46, 0.12)
	Fast	<0.001	GWT	0.94 (0.47, 0.12)	0.70 (0.61, 0.18)	0.97 (0.32, 0.09)	0.98 (0.47, 0.13)

Results of genetic variance analysis, type of estimate applied (GCT: combined among- and within-twin pair component estimate, GWT: within-pair estimate) and intraclass correlation coefficients (ICCs). ICC MZ: ICCs of monozygotic (MZ) twins, ICC DZ: ICCs of dizygotic (DZ) twins, ICC MZ cn: ICCs of consecutive nights for each subject in MZ group, ICC DZ cn: ICCs of consecutive nights for each subject in DZ group. ICC results include: original sample ICC (upper percentile of bootstrapped data, median of bootstrapped data). *Analysis of variance not applicable (significant differences between the means in DZ and MZ twin set).

**Table 7:** **Genetic variance analysis, type of estimate applied (GCT: combined among- and within-twin pair component estimate, GWT: within-pair estimate) and intraclass correlation coefficients (ICCs) for spindle parameters in slow wave sleep**.

Variable	Type	*P*	Analysis	ICC MZ	ICC DZ	ICC MZ cn	ICC DZ cn
*EEG channel: F3A2*
Number of spindles	Slow*	−	−	0.94 (0.46, 0.12)	0.14 (0.61, 0.18)	0.92 (0.35, 0.09)	0.76 (0.46, 0.13)
	Fast	0.204	GWT	0.41 (0.53, 0.12)	0.45 (0.76, 0.14)	0.84 (0.41, 0.08)	0.93 (0.61, 0.11)
Spindle density	Slow*	−	−	0.96 (0.47, 0.12)	0.05 (0.62, 0.18)	0.96 (0.32, 0.08)	0.91 (0.46, 0.13)
	Fast	0.677	GWT	0.45 (0.55, 0.12)	0.51 (0.74, 0.17)	0.81 (0.35, 0.08)	0.89 (0.57, 0.11)
Spindle length	Slow	<0.001	GCT	0.98 (0.57, 0.11)	0.45 (0.69, 0.18)	0.92 (0.42, 0.08)	0.89 (0.49, 0.13)
	Fast	0.010	GWT	0.78 (0.44, 0.13)	0.42 (0.64, 0.19)	0.66 (0.35, 0.09)	0.85 (0.44, 0.13)
Spindle amplitude	Slow	<0.001	GCT	0.89 (0.45, 0.13)	0.16 (0.65, 0.20)	0.91 (0.34, 0.09)	0.78 (0.45, 0.13)
	Fast	<0.001	GCT	0.83 (0.48, 0.12)	−0.30 (0.61, 0.19)	0.88 (0.34, 0.08)	0.60 (0.47, 0.13)
Spindle frequency	Slow	0.052	GWT	0.91 (0.45, 0.12)	0.81 (0.66, 0.19)	0.93 (0.33, 0.08)	0.97 (0.48, 0.13)
	Fast	0.027	GWT	0.86 (0.49, 0.13)	0.73 (0.58, 0.18)	0.85 (0.35, 0.09)	0.90 (0.48, 0.13)
*EEG channel: P3A2*
Number of spindles	Slow	0.005	GCT	0.88 (0.42, 0.12)	0.45 (0.66, 0.19)	0.89 (0.34, 0.08)	0.87 (0.46, 0.13)
	Fast	0.049	GWT	0.54 (0.49, 0.11)	0.15 (0.71, 0.19)	0.90 (0.35, 0.09)	0.83 (0.54, 0.12)
Spindle density	Slow	0.030	GWT	0.90 (0.53, 0.13)	0.64 (0.70, 0.20)	0.93 (0.36, 0.09)	0.95 (0.50, 0.13)
	Fast	0.071	GWT	0.68 (0.45, 0.12)	0.21 (0.59, 0.18)	0.88 (0.34, 0.08)	0.81 (0.50, 0.13)
Spindle length	Slow	<0.001	GCT	0.88 (0.57, 0.11)	0.46 (0.70, 0.20)	0.88 (0.39, 0.08)	0.90 (0.47, 0.13)
	Fast	0.020	GWT	0.74 (0.48, 0.13)	0.47 (0.63, 0.19)	0.80 (0.34, 0.09)	0.94 (0.50, 0.13)
Spindle amplitude	Slow	0.004	GWT	0.83 (0.44, 0.12)	0.41 (0.63, 0.19)	0.79 (0.33, 0.09)	0.73 (0.48, 0.12)
	Fast	0.004	GCT	0.80 (0.49, 0.12)	0.29 (0.62, 0.19)	0.84 (0.35, 0.09)	0.56 (0.48, 0.13)
Spindle frequency	Slow	0.049	GWT	0.82 (0.45, 0.12)	0.69 (0.63, 0.19)	0.88 (0.33, 0.08)	0.93 (0.48, 0.13)
	Fast	0.001	GWT	0.94 (0.47, 0.13)	0.77 (0.64, 0.18)	0.93 (0.32, 0.08)	0.96 (0.48, 0.13)

Abbreviations explanation as in Table 6. *Analysis of variance not applicable (significant differences between the means in DZ and MZ twin set).

Considering fast sleep spindles, GVA revealed significant genetic control on variance of spindle length, amplitude and frequency during both stage 2 sleep and SWS. However, we found no genetic effects on fast spindle number and density during stage 2 sleep, whereas during SWS in the P3A2 channel genetic influence on variance was significant on fast spindle number (the effect was weak: *P* = 0.049), and only marginally significant on fast spindle density.

The mean ICC of all slow spindle parameters for night-to-night stability was similar in both groups: 0.91 in the MZ set compared to 0.88 in the DZ set. All these values were above the significance threshold (*P* = 0.01) set by bootstrapping analysis. According to the Landis and Koch (1977) benchmark, night-to-night stability in the MZ set was *almost perfect* for all but one slow spindle characteristic (it was *substantial* for spindle number in P3A2 channel during SWS). Night-to-night stability in the DZ set was *almost perfect* for all but four slow spindle parameters. It was *substantial* for spindle number in the F3A2 channel during SWS as well as for spindle amplitude in the P3A2 channel during stage 2 sleep, as well as for spindle amplitude in both channels during SWS. The mean ICC of all slow spindle parameters for within-pair resemblance was 0.91 in MZ twins and 0.35 in DZ twins. In the MZ set, within-pair similarity was always above the significance level, and according to the benchmark within-pair similarity was *almost perfect* for all slow spindle parameters. In the DZ set however, within-pair similarity was below the significance level for all parameters besides spindle frequency during SWS. In addition, within-pair similarity for multiple parameters was below the bootstrapped median value, so it was lower than expected by chance. Within-pair similarity was *almost perfect* only once and *substantial* only twice. ICC estimations of slow spindle within-twin-pair resemblance as well as night-to-night stability were similar for sleep stage 2 when compared to SWS.

Considering fast spindles, the mean ICC for night-to-night stability was similar in both groups: 0.86 in the MZ set, compared to 0.85 in the DZ set. All these values were above the bootstrapped significance threshold (*P* = 0.01). Night-to-night stability in the MZ set was *almost perfect* for all fast spindle characteristics, whereas in the DZ set it was *almost perfect* for all but spindle amplitude parameters. Night-to-night stability of fast spindle amplitude in the DZ set ranged from *moderate* to *substantial*, therefore our finding of significant genetic influence on fast spindle amplitude should be treated with caution. The mean ICC of all fast spindle characteristics for within-pair resemblance was 0.76 in MZ twins and 0.45 in DZ twins. Within-pair similarity in the MZ set was below the significance level only for spindle number and density in F3A2 during SWS. According to the benchmark, in MZ twins within-pair similarity was seven times *almost perfect*, ten times *substantial* and three times only *moderate*. In DZ set within-pair similarity was at most *substantial* (six times) and only these values were above significance level. Again, some values were below the bootstrapped median, so they were lower than expected by chance.

Within-pair similarity in MZ twins was the lowest for fast spindle quantification parameters: total number and density, especially in SWS. These lower ICC results were not influenced by night-to-night stability, which was always *almost perfect*.

## Discussion

In this study we present an automatic sleep spindle detection algorithm based on CWT, which carefully localizes fast and slow spindles frequency for each individual and estimates the signal amplitude for each investigated EEG channel. We used a validation data set of 18 naps and compared our solution against human scoring and a SIESTA spindle detector. While the SIESTA detector is a popular and well tested solution, it does not distinguish between slow and fast spindles. In addition, its detection threshold is not individually adjusted according to signal amplitude (see “SIESTA Algorithm” Section). During sleep stage 2, the agreement between human scorer and both detectors was *moderate*, whereas the agreement between detectors was *substantial*. During SWS, the agreement between detectors was *moderate*. Due to observed differences between spindles scored by each algorithm, we found it interesting to apply our algorithm to sleep-related memory consolidation data previously analyzed with the SIESTA detector and described in Genzel et al. (2009). This experiment did not significantly improve our knowledge about spindles and memory consolidation, but we saw how technical differences can influence the analysis outcome. We confirmed significant positive correlation between spindle density and declarative memory consolidation, but we did not reproduce a significant positive correlation between spindle activity and declarative memory consolidation. Finally, comparison of basic spindle parameters between a group of 32 healthy MZ and 14 DZ same-gender twins revealed strong genetic influence on the variability of all slow spindle parameters, fast spindle morphology, and a weaker genetic effect on variance of fast spindle quantification parameters.

In our algorithm, we detect spindles with CWT using the Morlet wavelet, since wavelets of this type were shown to catch sleep spindle characteristics very well (Zygierewicz et al., 1999). Our solution rejects periods of signal with strong muscle artifacts as well as segments dominated by alpha activity. Furthermore, our method of adjusting spindle detection threshold was designed to reflect background signal amplitude as independent of signal/sleep quality and temporary events as much as possible. For this reason, signal activity was filtered below 6 Hz to avoid the influence of delta waves and k-complexes, and above 18 Hz to exclude possible muscle artifacts. In addition, logarithm transformation of frequency spectra, combined with usage of median instead of a mean, should decrease the influence of temporary activity bursts and frequency peaks. However, thresholds computed with our algorithm during stage 2 sleep were on average 9% lower than thresholds computed for SWS, so our threshold adjustment method is still sleep quality/stage dependent. We are not aware how different sleep stages influence adjustable thresholds used in other algorithms, but our conclusion is that, to avoid unnecessary variance among sleep recordings, thresholds based on general signal amplitude should be computed using homogenous sleep stage.

Our automatic adjustment of sleep spindle frequency boundaries is based on comparison of parietal and frontal EEG signals, like that proposed by Bódizs et al. (2009, 2012), but instead of frequency spectra our method analyses the frequency of pre-localized spindle events. Since this approach filters out all unnecessary parts of the signal it may be more exact, especially when sleep spindle density is low. Furthermore, our solution is robust against possible amplitude differences between channels. We observed considerable inter-subject variation in both slow and fast sleep spindle frequency. In addition, the average frequency of slow sleep spindles during SWS was slower than during stage 2 sleep which suggests that spindle frequency ranges should be set separately for shallow and deep sleep. The frequency distribution of pre-localized spindle events as well as estimated spindle frequency ranges for each twin can be found in Supplementary Material.

We compared spindle detection of our new algorithm with a human scorer and the commercially available SIESTA spindle detector, which was developed using a large database with manually scored sleep spindles (Anderer et al., 2005). One limitation in this study is that we did not compare scorings on an independent test set. We set detection thresholds using the validation set in order to match the sensitivity of the SIESTA algorithm. Our comparison results could thus be inflated due to an overfitting problem. The comparison of our solution with other algorithms and human scorers using an independent dataset should be the next step in future work. According to published benchmarks for the kappa coefficient (Landis and Koch, 1977), during sleep stage 2 the agreement between a human scorer and both algorithms was *moderate*, while both algorithms scored significantly more spindles. The agreement between algorithms was *substantial* during sleep stage 2 and dropped to *moderate* during SWS. In particular, the agreements with the human scorer seemed low and as presented in Figure 6, even manipulation of detection thresholds would not improve the agreement significantly. When we compared automatic algorithms we observed that spindles marked only by the SIESTA detector were either short or had the lowest amplitude, whereas spindles marked only by the SIESTA detector had a lower frequency, around 12 Hz, and higher activities in EEG background. Spindles marked only by the human scorer were the longest and had a very low amplitude in frequency spectrum. This low amplitude was problematic, since the human scored clearly the lowest amount of spindles, which means that the human detection threshold was the highest. The reason for the low average amplitude in the frequency spectrum was that marked events were often longer than activity in the sigma range. Spindles marked only by a visual scorer were rare, only 11% of total spindles scored. However, characteristics of these spindles show that visual scoring is prone to mistakes/inconsistencies. Since a sleep spindle is a very characteristic element of an EEG signal, this result seems to be disappointing. However, low spindle detection agreement is surprisingly a general phenomenon. Wendt et al. (2015) reported the average intra-expert agreement and inter-expert agreement measured with kappa at 0.66 and 0.52, respectively. Warby et al. (2014) reported that agreement between gold standard (consensus of human experts) and automatic algorithms measured with kappa ranged from 0.15–0.41 and pointed that the agreement between automatic detectors was generally lower than their agreement with the gold standard. Consistent high discrepancies between scorers indicate that even a small difference in detection approach results in a significantly different type of scored events. Unfortunately, simple differences in sensitivity between scorers only partially explain the problem. As Warby et al. (2014) observed: *“automated methods as a group were not consistent among themselves: they did not find the same “hidden” spindles”*. Automatic detectors use various signal processing techniques, spindle frequency ranges and decision-making processes. All these variables add up to significantly different detection results. Whereas most human scorers seem to share the decision process, according to Warby et al. (2014), experts *“frequently rely on spindles being a ‘distinct train of waves’ that is clearly distinguishable from background”.* The general human tendency to score spindles with a clearly strong spindle activity compared to other frequencies is most likely the main reason why inter-expert agreement is higher than agreement between automatic methods as well as between automatic methods and human scorers. There are already algorithms which mimic this approach, including ones proposed by Huupponen et al. (2007), and the SIESTA detector used to validate our algorithm. However, firstly, the average inter-expert agreement is still only *moderate*, and second, human visual scoring is usually performed on “raw” EEG signal, while all automatic methods use filtering or various transformations to extract activity in the spindle frequency range. Since we are not aware of any physiological data supporting the notion that spindles should dominate the frequency spectrum, our algorithm detects also spindles which are intermingled in other frequencies.

Low agreement between spindle detection methods combined with the highly individual character of sleep spindles (Werth et al., 1997), as well as the whole frequency spectrum (Buckelmüller et al., 2006), may lead to heterogeneous discrepancies in estimated spindle activity across subjects. As the result, the by-subject correlation of spindle activity estimated by different detection methods can be low. Warby et al. (2014) reported that the correlation between by-subject spindle density estimated from the gold standard and from the best automated detector was only *r* = 0.62. This fact leads to the question whether results of experiments are reproducible. For this reason, we re-analyzed sleep-related memory consolidation data, previously analyzed with the SIESTA detector and described in Genzel et al. (2009). The design of this project could be especially susceptible to these sort of discrepancies, since the idea was to correlate by-subject spindle activity estimations with memory retention. By-subject correlation between our algorithm and SIESTA detector did not fall below *r* = 0.80 neither for validation nor for memory consolidation data, and using our algorithm we reproduced almost all findings reported in Genzel et al. (2009). However, the highest discrepancy in by-subject correlation of spindle activity estimated by both algorithms and memory retention results was observed for spindle activity in sleep stage 2. It was surprising, since by-subject correlation of this parameter estimated by both algorithms was relatively high (*r* = 0.94). It shows that even small differences in spindle detection might lead to significantly different conclusions derived from an experiment. Significant discrepancies between spindle scorings increase the value of perfect reproducibility of the method and findings, provided by every automatic algorithm. For this reason, we conclude that the application of automatic algorithms for spindle detection in research projects should be encouraged.

Analysis of the twin data revealed high ICCs for night-to-night stability across investigated fast and slow spindle parameters during both sleep stage 2 and SWS, supporting previous reports about sleep spindle fingerprint characteristics (Werth et al., 1997; De Gennaro et al., 2005). Recently Eggert et al. (2015) reported ICC results for night-to-night stability of sleep spindles detected during stage 2 sleep with the SIESTA algorithm. The authors reported results from the central channel without distinction between slow and fast spindles. ICCs for night-to-night stability were also high for all spindle characteristics. The highest stability with ICC (0.92) was observed for spindle amplitude, and the lowest stability with ICC (0.84) was observed for spindle density. In our analysis we distinguished between slow and fast sleep spindles and we performed the analysis during stage 2 and during SWS separately. When comparing fast and slow spindles we observed that, besides frequency, stability of fast spindle parameters was moderately lower. This lower night-to-night stability of fast spindles dropped slightly further when we looked into within-pair similarity of MZ twins. Interestingly, the drop off between night-to-night stability and within-pair similarity of MZ twins was not observed for slow spindle parameters, where ICC estimates were on average exactly the same. In DZ twins, within-pair similarity was clearly lower than their night-to-night stability for both fast and slow spindle parameters. As a result, GVA revealed genetic control on variance of all slow and most of fast spindle parameters during stage 2 sleep and SWS. However, the genetic component of fast spindle parameters, besides spindle frequency, was weaker, especially for fast spindle quantities. GVA did not show significant genetic determination of fast spindle number and density during sleep stage 2. Analyses repeated with a subgroup of MZ twins closely matched for age, gender and cohabitation to DZ twins confirmed our findings in the total twin sample (see Supplementary Material, Tables S20–S23). In addition, for matched MZ sample GVA could be performed on slow spindle amplitude in SWS as well as slow spindle quantities in SWS. For all these remaining parameters we found significant genetic influence.

The number of DZ twin pairs (*n* = 14) is a limitation of our study. It is the reason why there is high variability of within-pair similarity estimates between spindle parameters in DZ twins. With our sample size, strong dissimilarity within just one twin pair strongly affects ICC outcome for the whole group. Sometimes these values were very high, above the bootstrapped significance threshold (*P* = 0.01), but sometimes these values were below similarity expected by chance (median of bootstrapped data) or even below zero, which means that resemblance between DZ twins was lower than observed in the population. Such low similarities within DZ twins has little biological sense and most likely could occur due to the small sample size. If we would compute narrow sense heritability, error margins would be high due to the small size of the DZ sample. Therefore, we do not provide narrow sense heritability estimations. Furthermore, we did not correct our GVA results for multiple testing, so there is an increased probability of type 1 error.

The next limitation of our study is the fact that we compared our algorithm to the SIESTA detector and human scorer using only fixed spindle detection frequency ranges. While individually adjusted frequency ranges may improve the quality of spindle detection, this change in the algorithm could result in significant detection differences. To illustrate how such change influences the detection, we provided the comparison of our algorithm with itself, with and without adjustable frequency ranges. The agreement was *almost perfect* when we considered all sleep spindles together during stage 2 sleep. However, the agreement dropped during SWS, since the detector with individually adjusted frequency ranges marked more spindles. This was because individually adjusted frequency ranges in SWS were often lower than 11 Hz, which was the lower boundary in the detector with fixed ranges. The agreement dropped further when we analyzed slow and fast spindles separately, since individually adjusted boundaries between fast and slow spindles varied and were rarely 13 Hz, like in fixed ranges approach. As a result, spindles classified as fast when using individually adjusted frequency ranges could be classified as slow when using fixed ranges. Ujma et al. (2015) compared a spindle detector with individually adjusted spindle frequency ranges vs. a different detector with fixed frequency ranges (slow spindles: 11–13 Hz, fast spindles: 13–15 Hz). The reported agreement was poor, especially for slow spindles. Differences between fixed and adjusted frequency ranges had a high impact on observed detection discrepancies. In many subjects individually adjusted fast spindle activity peak was approaching or fell below the 13 Hz threshold. Slow spindles seemed to be even more problematic. In approximately 25% of subjects the individually adjusted peak of slow spindle activity fell below 11 Hz, which is the commonly used lower boundary for spindle frequency. In order to compensate for the lack of validation of our adjustable frequency ranges vs. other methods, we provided detailed plots with detection results over frequency range for each twin in the Supplementary Material and in addition, we estimated genetic influence on sleep spindles also using fixed spindle detection frequency ranges. Results are included in Supplementary Material (Tables S12–S19). Due to fixed thresholds the separation between slow and fast spindles was less exact and therefore differences between two spindle types decreased. However, the outcome still supported our main observations about stronger night-to-night stability and stronger genetic influence on slow spindles when compared to fast ones.

Due to reported differences between spindle algorithms, as well as between human and automatic spindle scoring, spindle findings should be interpreted carefully. Our findings on strong genetic influence on spindle frequency, length and amplitude further promote the view that variability in the morphology of both slow and fast spindles is genetically driven. However, comparably weaker genetic effects on fast spindle quantity (density and total amount) may reflect stronger environmental influences on this spindle type (i.e., memory load). This is supported by a previous study on the role of fast spindles in sleep-dependent memory processing (Mölle et al., 2011). A detection algorithm which considers the individual morphology of two types of spindles may be an important tool to identify environmental influences on this relevant sleep phenomenon.

## Conflict of Interest Statement

The authors declare that the research was conducted in the absence of any commercial or financial relationships that could be construed as a potential conflict of interest.
