# Supplementary material for: Automatic Sleep Spindle Detection and Genetic Influence Estimation Using Continuous Wavelet Transform
Source: Front Hum Neurosci. 2015 Nov 19;9:624. doi: 10.3389/fnhum.2015.00624 (PMC4652604; doi:10.3389/fnhum.2015.00624)
Supplement: Supplementary file 1 [file DataSheet_1.pdf]

Supplementary Material

Automatic sleep spindle detection and heritability estimation using continuous wavelet transform

Authors: Marek Adamczyk[1]*, Lisa Genzel[2], Martin Dresler[1,3], Axel Steiger[1], Elisabeth Friess[1]

[1] Max Planck Institute of Psychiatry, Munich, Germany
[2] Centre for Cognitive and Neural Systems, University of Edinburgh, Edinburgh, UK
[3] Donders Institute for Brain, Cognition and Behaviour, Nijmegen, Netherlands

Correspondence:
Marek Adamczyk
Max Planck Institute of Psychiatry
Kraepelinstrasse 2-10,
80804 Munich,
Germany
E-mail: marek.adamczyk84@gmail.com

1. Supplementary Data

1.1. Similarity Measures

TP: true positives

TN: true negatives

FP: false positives

FN: false negatives

Sensitivity:

$$SE = \frac{TP}{TP + FN}$$

Specificity:

$$SP = \frac{TN}{TN + FP}$$

Precision:

$$PC = \frac{TP}{TP + FP}$$

All scores:

$$All_S = TP + FP + TN + FN$$

Accuracy:

$$ACC = \frac{TP + TN}{All_S}$$

Geometric mean:

$$Gm = \sqrt{SE \times SP}$$

The proportion of negative examples in the dataset:

$$N_N = \frac{FP + TN}{All_S}$$

Adjusted geometric-mean:

$$AGm = \frac{Gm + SP \times N_n}{1 + N_n}\ ;\ SE > 0$$

$$AGm = 0\ ;\ SE = 0$$

Matthews Correlation Coefficient:

$$MCC = \frac{TP \times TN - FP \times FN}{\sqrt{(TP + FN)(TP + FP)(TN + FP)(TN + FN)}}$$

Agreement hypothetically observed by chance:

$$\text{ACC}_{\text{chance}} = \frac{TP+FP}{\text{All}_S}\frac{TP+FN}{\text{All}_S} + \frac{FN+TN}{\text{All}_S}\frac{FP+TN}{\text{All}_S}$$

Cohen's kappa coefficient:

$$\kappa = \frac{\text{ACC - ACC}_{\text{chance}}}{1-\text{ACC}_{\text{chance}}}$$

1.2. Genetic variance analysis

In order to estimate genetic variance from the twin data, we applied a method proposed by Christian *et al*. (1974; 1987). This method is based on the general model for genetic variance estimation from twin data (Haseman and Elston, 1970). In this model, assuming that there is no evidence for inequality of the total phenotype variance of MZ and DZ twins, the expected mean squares should follow the equalities:

$$E(M_{amz} - M_{adz}) = E(M_{wdz} - M_{wmz}) = 0.5\sigma_a^2 + 0.75\sigma_d^2 + f\,0.5\sigma_i^2 \quad (1)$$

$$E(M_{amz} + M_{wmz}) = E(M_{adz} + M_{wdz}) = 2\sigma_a^2 + 2\sigma_d^2 + 2\sigma_i^2 + 2\sigma_e^2 \quad (2)$$

Where M is a mean square, amz: among MZ pairs, wmz: within MZ pairs, adz: among DZ pairs, wdz: within DZ pairs, σ_a^2: variance component due to additive genetic effects, σ_d^2: variance component due to dominant genetic effects, σ_i^2: variance component due to epistatic genetic effects, σ_e^2: variance component due to nonshared environmental effects. f : epistatic variance in DZ twins.

The first step of a genetic variance estimate, is to test, whether the assumption (2) of the model is valid by performing a two-tailed F' test comparing $M_{amz} + M_{wmz}$ with $M_{adz} + M_{wdz}$. If the sums of mean squares are significantly unequal, the environmental variance component σ_e^2 could be unequal for MZ and DZ twins. If there is a substantial genetic or environmental variance, it may be difficult to detect differences in σ_e^2. Therefore, as suggested by the authors (Christian *et al.*, 1974), the null hypothesis of equal variances was tested using alpha=0.2.

If the variances of MZ and DZ twins are not significantly different, the general model is appropriate and the genetic variance from equation (1) can be estimated using the within-

twin-pair estimate $GWT = M_{wdz} - M_{wmz}$ as well as the among-twin-pair estimate $GAT = M_{amz} - M_{adz}$. The one-tailed F test is performed to test whether the genetic variance is significant using the ratio of within-twin-pair mean squares M_{wdz} / M_{wmz}. Within-twin-pair mean squares are used, because typically their values are lower than among-twin-pair mean squares, and thus testing within-twin-pair mean squares ratio results in a more sensitive test.

If the variances of MZ and DZ twins are significantly unequal, the general model (1) has to be extended with the unique environmental effects in MZ σ^2_{emz} and DZ σ^2_{edz} twin set:

$$E(M_{amz} - M_{adz}) = [0.5\sigma^2_a + 0.75\sigma^2_d + f\,0.5\sigma^2_i] + (\sigma^2_{emz} - \sigma^2_{edz}) \qquad (3)$$

$$E(M_{wdz} - M_{wmz}) = [0.5\sigma^2_a + 0.75\sigma^2_d + f\,0.5\sigma^2_i] + (\sigma^2_{edz} - \sigma^2_{emz}) \qquad (4)$$

In this case, GWT as well as GAT estimation of genetic variance would be biased in the opposite directions. Therefore, in order to obtain the unaffected genetic variance estimation, the mean of GWT and GAT estimates is used. This combined estimator is called by the authors (Christian *et al.*, 1974) GCT:

$$GCT = \frac{GAT + GWT}{2} = \frac{(M_{amz} - M_{adz}) + (M_{wdz} - M_{wmz})}{2} = \frac{(M_{amz} + M_{wdz}) - (M_{adz} + M_{wmz})}{2}$$

(5)

The one-tailed F test is performed to test, whether the genetic variance is significant using the ratio $(M_{amz} + M_{wdz})/(M_{adz} + M_{wmz})$.

The GCT estimate is always appropriate, however it is much less powerful due to high possible values of among-twin-pair mean squares. Therefore the significance of GCT estimate is tested when there is evidence for unequal sums of mean squares in MZ and DZ twins, otherwise the significance of GWT estimate is tested.

Genetic variance analysis was performed on mean results of 2 recording nights. As a prerequisite for the analysis, each studied variable had to fulfill the assumptions of normal distribution (measured by a non-significant goodness-of-fit by the Kolmogorov-Smirnov test) in both twin samples and equal means between the twin samples (T-test). If the data did not fulfill the normal distribution criterion, they were log transformed prior to any analysis. The significantly unequal means between MZ and DZ twin samples indicate that the investigated variable could be associated with the type of twins being studied. In this case the estimation of

genetic variance would be biased. Therefore, if there was an evidence for significantly unequal means between MZ and DZ twin samples, the genetic variance analysis was not performed. Prerequisites were considered to be violated, if the appropriate test showed a significant result at the 5% level. Influence of covariates was tested by multivariate analysis of covariance (MANCOVA). Three covariates were considered:

1. Cohabitation (twins living together), since in former twin studies it was found to have a possible synchronizing effect on sleep architecture (Linkowski et al., 1989).
2. Age, since age has an effect on EEG power (Dijk et al., 1989a; Tarokh et al., 2011; EEG power decreases with age). Furthermore, younger twin pairs could show higher similarity of investigated parameters.
3. Sex, since it has an effect on EEG power (Dijk et al., 1989b; higher EEG power in females).

There was no significant difference between MZ and DZ twins in respect of all covariates:

1. Cohabitation: 15 of 32 MZ (47%) and 10 of 14 DZ (71%) twin pairs were living together at the time of the examination. Fisher exact test: P=0.19.
2. Age: MZ twins (mean (SD): 23.8 (4.8) years) were on average 1.7 years older than DZ twins (22.1 (2.7) years). Two-tailed T-test: P=0.28.
3. Sex: equally distributed over MZ (16 male pairs, 16 female pairs) and DZ twins (7 male pairs, 7 female pairs).

Here we report the results of spindle detection with individually adjusted spindle frequency ranges. Firstly, we performed MANCOVA to test, whether covariates age and sex have an effect on mean values of sleep spindle parameters (dependent variables) with zygosity as independent variable. Each spindle parameter (number, density, amplitude, length, frequency) was tested separately, using together data from four EEG channels (Fp1A2, F3A2, C3A2, P3A2) and two sleep stages (stage 2, slow wave sleep). We observed marginally significant covariate effect of age on fast spindle density ($F_{(8,\ 35)}$=1,871, P=0.96) and marginally significant covariate effect of sex on slow spindle number ($F_{(8, 35)}$=1,905, P=0.91).

Second, we performed MANCOVA to test, whether covariates age and cohabitation have an effect on variability of sleep spindle parameters within twin pairs (dependent variables) with zygosity as independent variable. As variability measure we used the absolute difference of a parameter values between twin partners. Variability of each spindle parameter (number,

density, amplitude, length, frequency) was tested separately, using together data from four EEG channels (Fp1A2, F3A2, C3A2, P3A2) and two sleep stages (stage 2, slow wave sleep). We did not observe significant covariate effect of age and cohabitation on variability of any sleep spindle parameters within twin pairs.

We observed just marginal effects of covariates on mean values of sleep spindle quantification parameters. However, to decrease their possible effect, we repeated the genetic variance analysis on a subgroup of MZ twins closely matched for age, gender and cohabitation to DZ twins (Supplement Tables S20-S23).

Christian, J. C., Kang, K. W., Norton, J. J. Jr. (1974). Choice of an estimate of genetic variance from twin data. *Am. J. Hum. Genet*. 26, 154–161.

Christian, J. C., Borhani, N. O., Castelli, W. P., Fabsitz, R., Norton, J. A. Jr., Reed, T., et al. (1987). Plasma cholesterol variation in the National Heart, Lung and Blood Institute Twin Study. *Genet. Epidemiol*. 4, 433–446.

Dijk, D. J., Beersma, D. G., van den Hoofdakker, R. H. (1989a). All night spectral analysis of EEG sleep in young adult and middle-aged male subjects. Neurobiol. Aging. 10, 677–682.

Dijk, D. J., Beersma, D. G., Bloem, G. M. (1989b). Sex differences in the sleep EEG of young adults: visual scoring and spectral analysis. Sleep. 12, 500–507.

Haseman, J. K. and Elston, R. C. (1970). The estimation of genetic variance from twin data. *Behav. Genet*. 1, 11–19.

Linkowski, P., Kerkhofs, M., Hauspie, R., Susanne, C., Mendlewicz, J. (1989). EEG sleep patterns in man: a twin study. *Electroencephalogr. Clin. Neurophysiol*. 73, 279–284.

1.3. Preprocessing before spindle detection

Here, we describe more in detail what was the reasoning behind our preprocessing methods. Exact preprocessing procedures are described in the main manuscript.

The exclusion of EEG signal fragments where occurrence of sleep spindles is unlikely was applied in multiple previously published algorithms. We focused on solutions described by Schimicek et al. (1994) and Huupponen et al. (2007). Schimicek algorithm computed root-mean-square (RMS) on 5 s fragments for activity in 30-40 Hz range (putative muscles activity), 11.5-16 range (spindle activity) and 8-12 Hz range (alpha activity). Schimicek was rejecting the epoch either if RMS(muscle) exceeded 5 μV or when RMS(alpha) /

RMS(spindle) > 1.2. Another approach was described by Huupponen who proposed "*sleep depth measure*" (SDP). Briefly, SDP was measuring the mean frequency in 0.5-12 Hz range using amplitude spectra for each second (see Hupponen et al. (2007) for details.). In order to determine SDP, mean frequency results for each second were smoothed using 31 s median filtering, and values smaller than 6 Hz indicated sleep.

1.3.1.Artifact exclusion

In order to detect fragments with muscle contamination, we also decided to look for strong activities in high frequencies, similarly to Schimicek et al. (1994). Our artifact detector rejects only fragments with very strong disturbances and our threshold settings were based on visual signal inspection. Our method, in general, is similar to Schimicek algorithm. However, our solution searches for disturbances in wider frequency ranges (band-pass FIR filter; -3 dB at 19.8 and 45.5 Hz) and within shorter time windows (1 s). We found that sometimes frequency of muscle contamination fell below 30 Hz, so wider range should be more robust. Furthermore, shorter time window seemed more sensitive.

1.3.2. Exclusion of segments with strong alpha activity

Both solutions, Schimicek et al. (1994) and Huupponen et al. (2007), presented interesting approaches to exclude wake/shallow sleep and fragments contaminated with alpha activity. We tried to combine both these solutions. SDP presented by Huupponen et al. (2007) tried to estimate, whether subject sleeps. However, we were not interested as much in excluding signal from wake as we were interested in excluding signal from mostly wake and stage 1 sleep contaminated with high alpha activity. Delta activity during wake and shallow sleep should be low. Therefore, we decided to compare the average $alpha_{activity}$ with $delta_{activity}$. Alpha activity tends to last on long fragments of EEG signal, therefore, similarly to Huupponen et al. (2007), we also evaluated alpha activity using long, 15 s sliding windows (half a minute seemed a bit too long). In addition, we also added the solution similar to the one proposed by Schimicek et al. (1994), where alpha activity is compared to sigma activity. For this reason, we computed additionally $sigma_{activity}$ on 12-16 Hz range (from amplitude spectra, procedure was exactly the same as for $alpha_{activity}$ and $delta_{activity}$, see the manuscript).
We had the condition:

$$alpha_{activity} > \alpha \times maximum([delta_{activity} , sigma_{activity}])$$

So in order to accept the fragment for further analysis, either $delta_{activity}$ or $sigma_{activity}$ multiplied by α had to be larger than $alpha_{activity}$. Solution worked nicely and the sensitivity was dependent on α parameter. We did not have a database with marked alpha activity. But empirically we saw what α values make sense. We were interested in excluding possibly

many fragments with alpha activity from wake and shallow sleep, but not from deep sleep, especially since sleep spindles in slow wave sleep tend to be slower. We used data from 16 nap recordings, 90 minutes each, of healthy male subjects (mean age = 23.3, SD = 0.8) to evaluate how many fragments of wakefulness and each stage our solution excludes. Nap recordings are especially useful in this case, since they have more wake and shallow sleep than whole night sleep recordings. Below, there is a statistics of used α values and percentages of each stage accepted for spindle detection.

α=1.00	artifacts: 58%	wake: 42%	stage 1: 83%	stage 2: 98%	sws: 100%	REM: 96%
α=1.10	artifacts: 61%	wake: 49%	stage 1: 87%	stage 2: 99%	sws: 100%	REM: 97%
α=1.20	artifacts: 64%	wake: 57%	stage 1: 89%	stage 2: 100%	sws: 100%	REM: 97%
α=1.30	artifacts: 67%	wake: 64%	stage 1: 91%	stage 2: 100%	sws: 100%	REM: 99%
α=1.40	artifacts: 70%	wake: 70%	stage 1: 93%	stage 2: 100%	sws: 100%	REM: 99%
α=1.50	artifacts: 71%	wake: 76%	stage 1: 95%	stage 2: 100%	sws: 100%	REM: 99%
α=1.60	artifacts: 72%	wake: 81%	stage 1: 96%	stage 2: 100%	sws: 100%	REM: 99%

When we evaluated $delta_{activity}$ and $sigma_{activity}$ separately, we saw that $sigma_{activity}$, when combined with $delta_{activity}$, has very little impact on the outcome.
For the condition:

$$alpha_{activity} > \alpha \times delta_{activity}$$

we had:

α=1.00	artifacts: 59%	wake: 44%	stage 1: 82%	stage 2: 99%	sws: 100%	REM: 95%
α=1.10	artifacts: 63%	wake: 52%	stage 1: 86%	stage 2: 99%	sws: 100%	REM: 96%
α=1.20	artifacts: 65%	wake: 60%	stage 1: 89%	stage 2: 100%	sws: 100%	REM: 97%
α=1.30	artifacts: 69%	wake: 67%	stage 1: 92%	stage 2: 100%	sws: 100%	REM: 99%
α=1.40	artifacts: 70%	wake: 73%	stage 1: 94%	stage 2: 100%	sws: 100%	REM: 99%
α=1.50	artifacts: 71%	wake: 79%	stage 1: 95%	stage 2: 100%	sws: 100%	REM: 99%
α=1.60	artifacts: 72%	wake: 84%	stage 1: 97%	stage 2: 100%	sws: 100%	REM: 99%

Very small differences in performance encouraged us to resign from $sigma_{activity}$ and our algorithm just compared the ratio of $alpha_{activity}$ and $delta_{activity}$. We chose α=1.1 as a sensitive solution which still excludes very small amount of sleep fragments.

Huupponen, E., Gomez-Herrero, G., Saastamoinen, A., Varri, A., Hasan, J., Himanen, S. L. (2007). Development and comparison of four sleep spindle detection methods. *Artif. Intell. Med*. 40, 157–170. doi: http://dx.doi.org/10.1016/j.artmed.2007.04.003

Schimicek, P., Zeitlhofer, J., Anderer, P., Saletu, B. (1994). Automatic sleep-spindle detection procedure: Aspects of reliability and validity. *Clin. Electroencephalogr*. 25, 26–29.

2. Supplementary Figures and Tables

Heritability estimation of sleep spindle activity.
Using whole MZ set:

2.1. Supplementary Tables

Supplementary Table S1: Individually adjusted frequency ranges in MZ twins part 1.

	Slow spindles				Fast spindles			
Subject ID	*Stage 2*		*SWS*		*Stage 2*		*SWS*	
	Night 2	*Night 3*	*Night 2*	*Night 3*	*Night 2*	*Night 3*	*Night 2*	*Night 3*
MZ twins								
sigt01a	10.3, 13.3	10.5, 13.5	9.2, 13.0	9.2, 13.4	13.5, 14.8	13.7, 14.9	13.2, 14.5	13.6, 14.7
sigt01b	9.8, 13.6	10.0, 13.7	9.2, 12.6	9.2, 13.4	13.8, 15.0	13.9, 15.2	13.6, 14.8	13.7, 14.9
sigt03a	10.8, 12.2	10.5, 12.1	10.5, 12.1	10.5, 12.2	12.4, 13.5	12.3, 13.3	12.3, 13.4	12.4, 13.5
sigt03b	10.8, 12.3	10.9, 12.3	10.5, 12.2	10.7, 12.2	12.5, 13.6	12.5, 13.6	12.4, 13.5	12.4, 13.5
sigt05a	11.0, 12.8	10.7, 12.8	10.5, 12.6	10.5, 12.5	13.0, 14.1	13.0, 14.1	12.8, 13.8	12.7, 13.8
sigt05b	10.9, 12.4	10.8, 12.5	9.6, 12.4	9.7, 12.4	12.6, 13.6	12.7, 13.7	12.6, 13.6	12.6, 13.6
sigt06a	11.2, 13.2	11.2, 13.6	11.0, 13.5	10.9, 13.1	13.5, 15.0	13.8, 14.9	13.8, 14.8	13.8, 14.8
sigt06b	11.1, 13.2	11.1, 13.1	9.4, 13.6	10.9, 13.0	13.4, 14.6	13.4, 14.6	13.8, 14.8	13.2, 14.3
sigt08a	10.6, 11.9	10.5, 11.8	9.3, 12.1	9.2, 11.9	12.1, 13.2	12.0, 13.0	12.3, 13.3	12.1, 13.1
sigt08b	10.7, 11.9	10.7, 12.0	9.3, 12.4	9.4, 13.3	12.1, 13.1	12.2, 13.3	12.6, 13.6	13.5, 14.5
sigt10a	9.3, 12.1	9.6, 12.2	10.3, 12.0	10.1, 12.0	12.6, 13.6	12.5, 13.6	12.4, 13.5	12.4, 13.4
sigt10b	9.6, 12.4	9.9, 12.4	9.7, 12.3	10.3, 12.3	12.6, 13.6	12.7, 13.7	12.5, 13.6	12.6, 13.6
sigt11a	10.7, 12.2	10.7, 12.3	10.9, 12.1	10.7, 11.9	12.4, 13.4	12.5, 13.5	12.3, 13.4	12.1, 13.2
sigt11b	10.6, 12.2	10.6, 12.3	11.1, 12.9	10.7, 12.1	12.4, 13.4	12.5, 13.5	13.1, 14.9	12.3, 13.3
sigt14a	11.2, 12.7	11.1, 12.7	9.5, 12.6	9.7, 12.6	12.9, 14.0	12.9, 14.1	12.8, 13.9	12.8, 13.9
sigt14b	11.3, 13.0	11.5, 13.0	9.8, 13.0	10.1, 12.8	13.2, 14.3	13.2, 14.2	13.2, 14.2	13.0, 14.0
sigt16a	11.3, 13.1	11.6, 13.1	9.2, 12.8	9.2, 13.0	13.3, 14.4	13.3, 14.4	13.0, 14.0	13.2, 14.4
sigt16b	11.1, 13.0	11.0, 12.9	9.2, 12.8	9.3, 12.8	13.4, 14.5	13.2, 14.4	13.4, 14.5	13.2, 14.3
sigt21a	11.1, 12.9	10.8, 12.8	11.2, 13.0	10.6, 12.8	13.1, 14.2	13.0, 14.0	13.2, 14.4	13.0, 14.0
sigt21b	10.9, 12.9	10.7, 12.7	10.6, 12.9	10.5, 12.7	13.1, 14.2	12.9, 14.1	13.1, 14.2	12.9, 13.9
sigt23a	11.1, 12.8	11.1, 12.8	10.3, 12.5	10.4, 12.6	13.0, 14.1	13.2, 14.2	12.7, 13.7	12.8, 14.0
sigt23b	10.9, 12.7	11.2, 13.0	10.9, 12.4	11.1, 12.8	12.9, 14.0	13.2, 14.3	12.6, 13.7	13.0, 14.1
sigt24a	10.2, 11.9	10.4, 11.9	9.8, 11.7	10.2, 11.6	12.1, 13.3	12.1, 13.3	11.9, 13.0	11.8, 12.8
sigt24b	10.4, 11.9	10.3, 11.7	9.9, 11.8	10.0, 11.7	12.1, 13.2	11.9, 13.1	12.0, 13.2	11.9, 12.9
sigt29a	10.6, 12.7	10.9, 12.8	10.8, 12.7	11.7, 12.6	12.9, 13.9	13.0, 14.1	12.9, 13.9	12.8, 13.9
sigt29b	11.0, 12.9	11.2, 13.1	11.3, 12.9	10.4, 13.0	13.1, 14.2	13.3, 14.4	13.1, 14.2	13.2, 14.2
sigt30a	11.4, 12.7	11.2, 12.6	11.1, 12.6	11.2, 12.5	12.9, 14.0	12.8, 13.9	12.8, 13.8	12.7, 13.7
sigt30b	11.3, 12.7	11.1, 12.6	11.1, 12.5	11.2, 12.5	12.9, 14.0	12.8, 13.9	12.7, 13.8	12.7, 13.7
sigt31a	11.5, 12.9	11.3, 12.7	11.4, 12.8	11.4, 12.8	13.1, 14.2	12.9, 14.0	13.0, 14.0	13.0, 14.1
sigt31b	11.6, 13.0	11.6, 13.0	10.8, 13.0	11.4, 13.1	13.2, 14.3	13.2, 14.2	13.2, 14.3	13.3, 14.3
sigt34a	11.0, 12.4	10.5, 12.3	9.1, 12.3	9.1, 12.6	12.6, 13.7	12.5, 13.6	12.5, 13.7	13.5, 14.5
sigt34b	10.9, 12.3	11.1, 12.6	9.1, 11.6	9.2, 12.1	12.5, 13.6	12.8, 13.9	11.8, 12.8	12.3, 13.3

Results in each column present begin and end of detection frequency range. DZ: dizygotic twins, MZ: monozygotic twins, SWS: slow wave sleep.

Supplementary Table S2: Individually adjusted frequency ranges in MZ twins part 2.

	Slow spindles				Fast spindles			
Subject ID	*Stage 2*		*SWS*		*Stage 2*		*SWS*	
	Night 2	*Night 3*	*Night 2*	*Night 3*	*Night 2*	*Night 3*	*Night 2*	*Night 3*
MZ twins								
sigt37a	10.7, 12.7	10.5, 12.7	10.7, 12.5	10.4, 12.7	12.9, 13.9	12.9, 14.0	12.7, 13.7	12.9, 14.0
sigt37b	10.8, 12.8	10.8, 12.9	10.5, 12.5	10.6, 12.7	13.0, 14.2	13.1, 14.1	12.8, 13.8	13.0, 14.0
sigt38a	9.3, 12.3	9.2, 12.4	9.1, 11.6	9.2, 11.8	12.5, 13.5	12.6, 13.8	11.8, 12.8	12.0, 13.0
sigt38b	9.2, 12.2	9.5, 12.4	9.2, 11.4	9.1, 11.6	12.4, 13.5	12.6, 13.7	13.5, 14.5	11.8, 12.8
sigt39a	11.1, 12.5	11.0, 12.6	11.7, 12.4	11.4, 12.6	12.7, 13.9	12.8, 13.9	12.6, 13.8	12.8, 13.9
sigt39b	11.0, 12.8	11.0, 12.7	11.1, 12.7	11.5, 12.7	13.0, 14.1	12.9, 14.0	12.9, 14.0	12.9, 13.9
sigt43a	10.4, 13.4	11.3, 13.5	10.4, 13.2	10.6, 13.4	13.6, 14.7	13.7, 14.7	13.4, 14.6	13.7, 14.8
sigt43b	11.3, 13.5	10.8, 13.5	11.0, 13.3	10.0, 13.1	13.8, 14.9	13.7, 14.8	13.5, 14.6	13.3, 14.3
sigt44a	10.9, 12.8	11.0, 13.0	10.0, 12.7	10.5, 12.9	13.0, 14.1	13.2, 14.3	12.9, 14.0	13.1, 14.2
sigt44b	10.7, 13.0	11.5, 13.2	10.4, 12.8	10.8, 13.2	13.2, 14.3	13.4, 14.5	13.0, 14.3	13.4, 14.4
sigt45a	10.4, 11.7	10.5, 11.7	9.1, 11.8	9.9, 11.7	11.9, 12.9	11.9, 13.0	12.0, 13.1	11.9, 13.0
sigt45b	10.6, 11.8	10.6, 11.8	9.2, 11.8	9.3, 11.6	12.0, 13.1	12.0, 13.1	12.0, 13.1	11.8, 13.1
sigt46a	10.3, 12.2	10.3, 12.1	10.4, 12.3	10.5, 12.2	12.4, 13.6	12.3, 13.5	12.5, 13.6	12.4, 13.6
sigt46b	10.4, 12.2	10.0, 12.2	10.6, 12.2	9.5, 12.1	12.4, 13.4	12.4, 13.5	12.4, 13.5	12.3, 13.3
sigt50a	11.1, 12.6	11.2, 12.6	11.5, 12.6	12.0, 12.8	12.8, 14.0	12.8, 14.0	12.8, 13.9	13.0, 14.1
sigt50b	11.2, 12.5	11.2, 12.4	11.1, 12.6	11.2, 12.6	12.7, 13.9	12.6, 13.9	12.8, 13.8	12.8, 13.9
sigt53a	11.4, 12.9	11.5, 12.9	10.5, 12.9	11.4, 12.9	13.1, 14.1	13.1, 14.2	13.1, 14.2	13.1, 14.3
sigt53b	11.2, 12.8	11.2, 12.9	10.1, 12.7	10.1, 12.7	13.0, 14.0	13.1, 14.1	12.9, 13.9	12.9, 13.9
sigt55a	10.4, 12.4	10.8, 12.5	10.4, 12.3	10.3, 12.6	12.6, 13.6	12.7, 13.7	12.5, 13.7	12.8, 13.9
sigt55b	10.5, 12.4	11.0, 12.2	10.4, 12.7	10.0, 12.4	12.8, 14.0	12.7, 13.8	12.9, 14.0	12.6, 13.6
sigt57a	10.3, 12.8	10.4, 12.6	9.7, 12.6	9.8, 12.6	13.4, 14.6	12.8, 14.5	13.2, 14.5	13.0, 14.3
sigt57b	10.4, 12.6	10.5, 12.6	9.4, 12.3	9.2, 12.3	12.9, 14.3	13.4, 14.4	12.9, 14.3	13.2, 14.4
sigt59a	11.3, 12.6	11.2, 12.5	11.4, 12.3	11.2, 12.2	12.8, 13.9	12.7, 13.9	12.5, 13.5	12.4, 13.8
sigt59b	11.3, 12.7	11.3, 12.7	11.2, 12.5	11.2, 12.4	12.9, 14.0	12.9, 13.9	12.7, 13.8	12.6, 13.7
sigt64a	10.4, 11.9	10.7, 12.0	10.6, 11.8	10.5, 11.9	12.1, 13.2	12.2, 13.4	12.0, 13.1	12.1, 13.1
sigt64b	11.0, 12.0	11.0, 12.1	10.8, 11.9	10.8, 12.0	12.2, 13.3	12.3, 13.4	12.1, 13.2	12.2, 13.3
sigt66a	11.4, 12.6	11.2, 12.5	11.3, 12.4	11.3, 12.4	12.8, 13.8	12.7, 13.7	12.6, 13.8	12.6, 13.7
sigt66b	11.5, 12.6	11.5, 12.6	11.2, 12.6	11.2, 12.5	12.8, 13.9	12.8, 13.9	12.8, 13.9	12.7, 13.8
sigt67a	11.4, 13.0	11.4, 12.9	11.3, 13.1	11.3, 13.1	13.2, 14.2	13.1, 14.2	13.3, 14.3	13.3, 14.3
sigt67b	11.7, 13.0	11.7, 12.8	11.6, 13.0	11.5, 12.8	13.2, 14.3	13.0, 14.1	13.2, 14.2	13.0, 14.2
sigt68a	9.4, 11.9	9.3, 11.7	9.9, 11.7	10.3, 11.6	12.1, 13.1	11.9, 12.9	11.9, 13.0	11.8, 12.8
sigt68b	9.3, 11.4	9.2, 11.6	9.3, 11.4	9.3, 11.5	11.6, 12.6	11.8, 12.8	11.6, 12.6	11.7, 12.7

Results in each column present begin and end of detection frequency range. DZ: dizygotic twins, MZ: monozygotic twins, SWS: slow wave sleep.

Supplementary Table S3: Individually adjusted frequency ranges in DZ twins.

	Slow spindles				Fast spindles			
Subject ID	*Stage 2*		*SWS*		*Stage 2*		*SWS*	
	Night 2	*Night 3*	*Night 2*	*Night 3*	*Night 2*	*Night 3*	*Night 2*	*Night 3*
DZ twins								
sigt02a	11.3, 13.0	11.2, 12.9	11.2, 13.1	11.2, 13.0	13.2, 14.2	13.1, 14.2	13.3, 14.4	13.2, 14.3
sigt02b	11.1, 13.2	11.0, 13.0	11.1, 13.3	11.1, 13.3	13.4, 14.4	13.2, 14.3	13.5, 14.6	13.5, 14.5
sigt04a	10.2, 12.1	10.1, 12.0	9.6, 12.1	9.3, 12.0	12.3, 13.3	12.2, 13.3	12.4, 13.4	12.2, 13.3
sigt04b	11.0, 12.9	11.1, 13.0	9.9, 12.8	9.6, 12.6	13.1, 14.3	13.2, 14.3	13.0, 14.1	12.8, 14.1
sigt07a	10.3, 12.2	10.7, 12.4	9.3, 12.1	10.9, 12.4	12.4, 13.5	12.6, 13.7	12.3, 13.3	12.6, 13.7
sigt07b	10.2, 12.0	10.3, 12.0	10.0, 12.0	9.8, 11.9	12.2, 13.2	12.2, 13.2	12.2, 13.2	12.1, 13.2
sigt12a	11.2, 12.5	11.1, 12.4	10.4, 12.2	10.2, 12.1	12.7, 13.8	12.6, 13.7	12.4, 13.4	12.3, 13.5
sigt12b	10.6, 12.4	10.6, 12.4	10.2, 12.3	10.0, 12.2	12.6, 13.7	12.6, 13.7	12.5, 13.6	12.4, 13.5
sigt18a	12.3, 13.3	11.8, 13.7	9.7, 13.1	9.3, 13.5	13.5, 14.6	13.9, 15.0	13.3, 14.3	13.7, 14.8
sigt18b	10.9, 13.2	11.1, 13.2	9.8, 13.0	9.5, 13.0	13.5, 14.7	13.4, 14.8	13.3, 14.5	13.4, 14.5
sigt28a	11.9, 13.1	12.0, 13.2	11.8, 13.2	12.2, 13.3	13.3, 14.3	13.4, 14.4	13.4, 14.4	13.5, 14.5
sigt28b	11.7, 13.1	11.8, 13.3	10.9, 13.4	10.7, 13.6	13.3, 14.4	13.5, 14.6	13.6, 14.7	13.8, 14.9
sigt33a	11.0, 12.9	11.2, 12.9	11.0, 12.8	11.1, 12.9	13.1, 14.2	13.1, 14.2	13.0, 14.1	13.1, 14.2
sigt33b	11.2, 13.0	11.1, 13.2	11.4, 13.0	11.2, 13.1	13.4, 14.4	13.4, 14.5	13.2, 14.3	13.3, 14.5
sigt47a	11.1, 12.5	10.5, 12.4	10.2, 12.4	10.0, 12.2	12.7, 13.7	12.6, 13.6	12.7, 13.8	12.4, 13.5
sigt47b	11.1, 12.5	11.0, 12.5	11.1, 12.6	11.0, 12.6	12.7, 13.7	12.7, 13.9	12.8, 13.9	12.8, 13.9
sigt49a	9.7, 12.6	9.4, 12.7	9.1, 12.6	9.1, 12.6	12.8, 13.8	12.9, 14.0	12.8, 13.9	12.8, 14.2
sigt49b	11.0, 13.3	10.8, 13.2	9.2, 13.0	9.2, 13.2	13.5, 14.5	13.4, 14.5	13.3, 14.4	13.5, 14.5
sigt52a	11.7, 13.2	11.7, 13.2	11.5, 12.3	11.4, 12.9	13.4, 14.5	13.4, 14.6	12.5, 13.7	13.1, 14.1
sigt52b	12.0, 13.1	11.7, 13.0	11.1, 12.9	11.1, 12.7	13.3, 14.4	13.2, 14.3	13.1, 14.2	12.9, 14.1
sigt54a	10.9, 12.7	11.0, 12.9	9.9, 12.6	10.0, 12.7	12.9, 14.0	13.1, 14.3	12.8, 14.0	12.9, 14.1
sigt54b	10.8, 12.6	11.0, 12.6	10.7, 12.6	10.8, 12.6	12.8, 14.0	12.8, 13.9	12.8, 14.2	12.8, 13.9
sigt58a	10.7, 11.9	10.7, 11.9	10.7, 11.9	10.3, 12.1	12.1, 13.1	12.1, 13.2	12.1, 13.2	12.3, 13.4
sigt58b	11.2, 12.2	11.3, 12.2	10.9, 12.3	10.8, 12.1	12.4, 13.5	12.4, 13.5	12.5, 13.5	12.3, 13.4
sigt62a	11.2, 12.6	11.2, 12.6	10.9, 12.5	11.0, 12.4	12.8, 13.9	12.8, 13.8	12.7, 13.7	12.6, 13.7
sigt62b	11.6, 13.1	11.9, 13.3	9.8, 12.9	9.6, 13.0	13.3, 14.4	13.5, 14.5	13.1, 14.1	13.2, 14.2
sigt63a	10.7, 12.2	10.7, 12.1	9.3, 12.1	9.6, 12.1	12.4, 13.5	12.3, 13.5	12.3, 13.4	12.3, 13.4
sigt63b	10.1, 11.5	10.1, 11.5	10.0, 11.5	10.1, 11.4	11.7, 12.8	11.7, 13.0	11.7, 12.8	11.6, 12.7

Results in each column present begin and end of detection frequency range. DZ: dizygotic twins, MZ: monozygotic twins, SWS: slow wave sleep.

Supplementary Table S4: Individually adjusted frequency ranges. Fast spindles in stage 2 sleep. Parameters Averaged Over Pairs.

	DZ n = 14			MZ n = 32		
	Night 2	*Night 3*	*2 nights mean*	*Night 2*	*Night 3*	*2 nights mean*
EEG channel: Fp1A2						
Number of spindles	255.32±45.07	240.68±43.34	248.00±43.11	266.55±39.84	272.20±38.55	269.38±37.96
Spindle density	0.56±0.10	0.53±0.09	0.55±0.10	0.55±0.08	0.56±0.08	0.56±0.08
Spindle length	0.69±0.01	0.68±0.01	0.69±0.01	0.68±0.01	0.68±0.01	0.68±0.01
Spindle amplitude (μV)	8.61±0.25	8.45±0.27	8.53±0.25	8.48±0.24	8.41±0.23	8.45±0.23
Spindle frequency	13.49±0.13	13.54±0.13	13.52±0.13	13.38±0.08	13.39±0.08	13.38±0.08
EEG channel: F3A2						
Number of spindles	557.50±70.88	544.75±68.75	551.13±68.74	600.20±65.97	602.19±61.97	601.20±62.38
Spindle density	1.21±0.16	1.21±0.15	1.21±0.15	1.25±0.13	1.26±0.13	1.25±0.13
Spindle length	0.76±0.02	0.75±0.01	0.75±0.01	0.73±0.01	0.74±0.01	0.73±0.01
Spindle amplitude (μV)	10.88±0.23	10.66±0.29	10.77±0.25	10.13±0.31	10.12±0.29	10.13±0.30
Spindle frequency	13.56±0.12	13.59±0.12	13.57±0.12	13.44±0.08	13.45±0.08	13.45±0.08
EEG channel: C3A2						
Number of spindles	1081.82±115.84	1064.61±101.64	1073.21±106.45	1191.45±93.90	1160.63±89.66	1176.04±89.68
Spindle density	2.35±0.25	2.35±0.21	2.35±0.22	2.51±0.19	2.43±0.18	2.47±0.18
Spindle length	0.86±0.02	0.86±0.02	0.86±0.02	0.84±0.02	0.83±0.02	0.84±0.02
Spindle amplitude (μV)	10.88±0.28	10.82±0.30	10.85±0.28	10.50±0.29	10.35±0.27	10.42±0.28
Spindle frequency	13.68±0.12	13.70±0.13	13.69±0.12	13.57±0.09	13.58±0.09	13.57±0.09
EEG channel: P3A2						
Number of spindles	1175.61±136.10	1150.75±117.96	1163.18±125.47	1218.08±96.00	1185.23±91.67	1201.66±91.14
Spindle density	2.54±0.27	2.53±0.24	2.54±0.25	2.59±0.20	2.51±0.19	2.55±0.19
Spindle length	0.89±0.03	0.89±0.02	0.89±0.02	0.86±0.02	0.85±0.02	0.86±0.01
Spindle amplitude (μV)	10.29±0.44	9.99±0.36	10.14±0.38	9.59±0.30	9.45±0.29	9.52±0.29
Spindle frequency	13.66±0.13	13.67±0.13	13.67±0.13	13.56±0.09	13.57±0.09	13.56±0.09

Group mean ± SEM. DZ: dizygotic twins, MZ: monozygotic twins.

Supplementary Table S5: Individually adjusted frequency ranges. Genetic variance analysis, type of estimate applied (GCT: combined among- and within-twin pair component estimate, GWT: within-pair estimate) and intraclass correlation coefficients (ICCs) for fast spindle parameters in stage 2 sleep.

Variable	*P*	GWT vs GCT	ICC MZ	ICC DZ	ICC MZ cn	ICC DZ cn
EEG channel: Fp1A2						
Number of spindles	.002	GWT	0.84(0.48, 0.12)	0.33(0.73, 0.17)	0.82(0.34, 0.09)	0.91(0.56, 0.12)
Spindle density	<.001	GWT	0.85(0.47, 0.12)	0.38(0.74, 0.18)	0.86(0.36, 0.09)	0.94(0.57, 0.13)
Spindle length	<.001	GWT	0.79(0.47, 0.12)	0.40(0.64, 0.17)	0.85(0.30, 0.09)	0.89(0.49, 0.13)
Spindle amplitude	.005	GCT	0.82(0.47, 0.12)	0.21(0.63, 0.18)	0.91(0.32, 0.08)	0.86(0.45, 0.13)
Spindle frequency	<.001	GWT	0.93(0.42, 0.11)	0.67(0.62, 0.18)	0.95(0.31, 0.09)	0.96(0.48, 0.13)
EEG channel: F3A2						
Number of spindles	.279	GWT	0.75(0.44, 0.13)	0.49(0.61, 0.18)	0.85(0.32, 0.08)	0.92(0.49, 0.12)
Spindle density	.164	GWT	0.78(0.48, 0.11)	0.54(0.67, 0.18)	0.86(0.32, 0.08)	0.94(0.50, 0.13)
Spindle length	.030	GWT	0.74(0.46, 0.12)	0.40(0.61, 0.17)	0.82(0.33, 0.08)	0.91(0.46, 0.13)
Spindle amplitude	<.001	GCT	0.88(0.45, 0.13)	0.10(0.62, 0.16)	0.88(0.32, 0.08)	0.74(0.50, 0.13)
Spindle frequency	<.001	GWT	0.93(0.42, 0.12)	0.67(0.64, 0.18)	0.96(0.33, 0.09)	0.96(0.48, 0.14)
EEG channel: C3A2						
Number of spindles	.443	GWT	0.75(0.44, 0.12)	0.61(0.65, 0.18)	0.89(0.31, 0.09)	0.82(0.49, 0.13)
Spindle density	.147	GWT	0.82(0.43, 0.12)	0.64(0.65, 0.19)	0.90(0.31, 0.08)	0.84(0.46, 0.13)
Spindle length	.005	GWT	0.82(0.47, 0.11)	0.55(0.67, 0.19)	0.93(0.32, 0.09)	0.93(0.48, 0.13)
Spindle amplitude	.002	GCT	0.88(0.44, 0.12)	0.33(0.65, 0.17)	0.90(0.32, 0.09)	0.83(0.49, 0.13)
Spindle frequency	<.001	GWT	0.95(0.44, 0.12)	0.66(0.65, 0.18)	0.97(0.33, 0.09)	0.97(0.48, 0.13)
EEG channel: P3A2						
Number of spindles	.271	GWT	0.80(0.45, 0.12)	0.70(0.68, 0.19)	0.83(0.34, 0.09)	0.86(0.46, 0.13)
Spindle density	.196	GWT	0.80(0.44, 0.12)	0.67(0.66, 0.19)	0.85(0.32, 0.09)	0.88(0.44, 0.12)
Spindle length	.002	GWT	0.78(0.45, 0.12)	0.48(0.62, 0.19)	0.89(0.33, 0.08)	0.92(0.47, 0.14)
Spindle amplitude	.047	GWT	0.82(0.48, 0.13)	0.57(0.67, 0.17)	0.88(0.33, 0.09)	0.64(0.48, 0.12)
Spindle frequency	<.001	GWT	0.94(0.47, 0.12)	0.70(0.61, 0.18)	0.97(0.32, 0.09)	0.98(0.47, 0.13)

Results of genetic variance analysis, type of estimate applied (GCT: combined among- and within-twin pair component estimate, GWT: within-pair estimate) and intraclass correlation coefficients (ICCs). ICC MZ: ICCs of monozygotic (MZ) twins, ICC DZ: ICCs of dizygotic (DZ) twins, ICC MZ cn: ICCs of consecutive nights for each subject in MZ group, ICC DZ cn: ICCs of consecutive nights for each subject in DZ group. ICC results include: original sample ICC (upper percentile of bootstrapped data, median of bootstrapped data).

Supplementary Table S6: Individually adjusted frequency ranges. Fast spindles in slow wave sleep. Parameters Averaged Over Pairs.

	DZ n = 14			MZ n = 32		
	Night 2	*Night 3*	*2 nights mean*	*Night 2*	*Night 3*	*2 nights mean*
EEG channel: Fp1A2						
Number of spindles	30.89±11.75	33.82±11.21	32.36±11.41	22.44±4.11	24.19±4.90	23.31±4.38
Spindle density	0.15±0.05	0.16±0.05	0.15±0.05	0.13±0.02	0.15±0.03	0.14±0.03
Spindle length	0.63±0.01	0.65±0.01	0.64±0.01	0.62±0.01	0.63±0.01	0.63±0.01
Spindle amplitude (µV)	8.75±0.30	8.79±0.29	8.77±0.27	8.85±0.34	8.49±0.26	8.67±0.25
Spindle frequency	13.32±0.14	13.25±0.16	13.28±0.16	13.18±0.08	13.15±0.09	13.16±0.09
EEG channel: F3A2						
Number of spindles	93.79±26.30	96.79±21.59	95.29±23.63	74.86±12.76	76.81±12.99	75.84±12.46
Spindle density	0.48±0.12	0.47±0.09	0.47±0.10	0.44±0.07	0.48±0.08	0.46±0.07
Spindle length	0.64±0.01	0.65±0.01	0.64±0.01	0.65±0.01	0.65±0.01	0.65±0.01
Spindle amplitude (µV)	11.30±0.22	10.86±0.22	11.08±0.18	10.40±0.39	10.21±0.38	10.25±0.39
Spindle frequency	13.41±0.13	13.42±0.13	13.42±0.13	13.26±0.08	13.31±0.09	13.30±0.08
EEG channel: C3A2						
Number of spindles	258.68±43.40	269.07±41.98	263.88±40.94	215.48±32.26	204.92±30.42	210.20±30.63
Spindle density	1.32±0.19	1.31±0.16	1.32±0.17	1.24±0.17	1.22±0.16	1.23±0.16
Spindle length	0.70±0.02	0.71±0.01	0.71±0.02	0.70±0.01	0.70±0.01	0.70±0.01
Spindle amplitude (µV)	10.70±0.21	10.78±0.22	10.74±0.19	10.44±0.28	10.28±0.30	10.36±0.28
Spindle frequency	13.59±0.12	13.60±0.13	13.59±0.13	13.50±0.09	13.52±0.09	13.51±0.09
EEG channel: P3A2						
Number of spindles	308.54±49.90	306.71±44.83	307.63±46.00	237.88±34.78	211.83±33.72	224.85±33.67
Spindle density	1.56±0.22	1.50±0.18	1.53±0.20	1.32±0.18	1.25±0.17	1.29±0.18
Spindle length	0.73±0.02	0.74±0.02	0.73±0.02	0.72±0.01	0.72±0.01	0.72±0.01
Spindle amplitude (µV)	9.83±0.33	9.66±0.27	9.74±0.28	9.32±0.28	9.13±0.29	9.23±0.28
Spindle frequency	13.59±0.13	13.63±0.13	13.61±0.13	13.54±0.09	13.51±0.09	13.53±0.09

Group mean ± SEM. DZ: dizygotic twins, MZ: monozygotic twins.

Supplementary Table S7: Individually adjusted frequency ranges. Genetic variance analysis, type of estimate applied (GCT: combined among- and within-twin pair component estimate, GWT: within-pair estimate) and intraclass correlation coefficients (ICCs) for fast spindle parameters in slow wave sleep.

Variable	*P*	GWT vs GCT	ICC MZ	ICC DZ	ICC MZ cn	ICC DZ cn
EEG channel: Fp1A2						
Number of spindles	.857	GCT	0.37(0.66, 0.10)	0.33(0.88, 0.14)	0.85(0.39, 0.07)	0.94(0.72, 0.11)
Spindle density	.071	GWT	0.47(0.60, 0.11)	0.40(0.88, 0.16)	0.82(0.37, 0.07)	0.88(0.69, 0.12)
Spindle length	.008	GWT	0.40(0.56, 0.16)	0.03(0.76, 0.22)	0.17(0.35, 0.10)	0.12(0.52, 0.13)
Spindle amplitude	.039	GWT	0.70(0.56, 0.16)	0.21(0.74, 0.23)	0.64(0.36, 0.11)	0.78(0.51, 0.15)
Spindle frequency	.005	GWT	0.87(0.57, 0.16)	0.71(0.75, 0.23)	0.88(0.42, 0.10)	0.94(0.49, 0.14)
EEG channel: F3A2						
Number of spindles	.204	GWT	0.41(0.53, 0.12)	0.45(0.76, 0.14)	0.84(0.41, 0.08)	0.93(0.61, 0.11)
Spindle density	.677	GWT	0.45(0.55, 0.12)	0.51(0.74, 0.17)	0.80(0.35, 0.08)	0.89(0.57, 0.11)
Spindle length	.010	GWT	0.78(0.44, 0.13)	0.42(0.64, 0.19)	0.66(0.35, 0.09)	0.85(0.44, 0.13)
Spindle amplitude	<.001	GCT	0.83(0.48, 0.12)	-0.30(0.61, 0.19)	0.88(0.34, 0.08)	0.60(0.47, 0.13)
Spindle frequency	.027	GWT	0.86(0.49, 0.13)	0.73(0.58, 0.18)	0.85(0.35, 0.09)	0.90(0.48, 0.13)
EEG channel: C3A2						
Number of spindles	.040	GWT	0.72(0.50, 0.13)	0.39(0.66, 0.18)	0.86(0.33, 0.08)	0.76(0.52, 0.13)
Spindle density	.010	GCT	0.77(0.46, 0.12)	0.41(0.65, 0.19)	0.87(0.32, 0.08)	0.75(0.49, 0.13)
Spindle length	.022	GWT	0.83(0.46, 0.12)	0.62(0.63, 0.19)	0.82(0.36, 0.08)	0.83(0.45, 0.13)
Spindle amplitude	<.001	GCT	0.84(0.45, 0.12)	-0.06(0.66, 0.19)	0.82(0.34, 0.09)	0.67(0.46, 0.13)
Spindle frequency	.003	GWT	0.91(0.45, 0.12)	0.72(0.69, 0.19)	0.93(0.34, 0.09)	0.94(0.49, 0.13)
EEG channel: P3A2						
Number of spindles	.049	GWT	0.54(0.49, 0.11)	0.15(0.71, 0.19)	0.90(0.35, 0.09)	0.83(0.54, 0.12)
Spindle density	.071	GWT	0.68(0.45, 0.12)	0.21(0.59, 0.18)	0.88(0.34, 0.08)	0.80(0.50, 0.13)
Spindle length	.020	GWT	0.74(0.48, 0.13)	0.47(0.63, 0.19)	0.80(0.34, 0.09)	0.94(0.50, 0.13)
Spindle amplitude	.004	GCT	0.80(0.49, 0.12)	0.29(0.62, 0.19)	0.84(0.35, 0.09)	0.56(0.48, 0.13)
Spindle frequency	.001	GWT	0.94(0.47, 0.13)	0.77(0.64, 0.18)	0.93(0.32, 0.08)	0.96(0.48, 0.13)

Results of genetic variance analysis, type of estimate applied (GCT: combined among- and within-twin pair component estimate, GWT: within-pair estimate) and intraclass correlation coefficients (ICCs). ICC MZ: ICCs of monozygotic (MZ) twins, ICC DZ: ICCs of dizygotic (DZ) twins, ICC MZ cn: ICCs of consecutive nights for each subject in MZ group, ICC DZ cn: ICCs of consecutive nights for each subject in DZ group. ICC results include: original sample ICC (upper percentile of bootstrapped data, median of bootstrapped data).

Supplementary Table S8: Individually adjusted frequency ranges. Slow spindles in stage 2 sleep. Parameters Averaged Over Pairs.

	DZ n = 14			MZ n = 32		
	Night 2	*Night 3*	*2 nights mean*	*Night 2*	*Night 3*	*2 nights mean*
EEG channel: Fp1A2						
Number of spindles	1372.25±127.32	1383.36±110.60	1377.80±115.36	1261.34±95.57	1270.30±90.89	1265.82±92.52
Spindle density	2.98±0.24	3.06±0.22	3.02±0.22	2.66±0.19	2.68±0.19	2.67±0.19
Spindle length	0.95±0.03	0.96±0.03	0.95±0.03	0.90±0.02	0.91±0.02	0.90±0.02
Spindle amplitude (μV)	8.75±0.26	8.60±0.28	8.68±0.27	8.29±0.25	8.30±0.23	8.30±0.24
Spindle frequency	11.49±0.11	11.49±0.11	11.49±0.11	11.36±0.08	11.39±0.08	11.38±0.08
EEG channel: F3A2						
Number of spindles	1376.86±126.30	1381.07±106.04	1378.96±113.80	1243.14±100.28	1226.30±90.80	1234.72±94.83
Spindle density	2.98±0.22	3.05±0.20	3.02±0.21	2.63±0.20	2.59±0.19	2.61±0.20
Spindle length*	0.95±0.02	0.96±0.03	0.96±0.02	0.89±0.02	0.89±0.02	0.89±0.02
Spindle amplitude (μV)**	10.63±0.26	10.56±0.37	10.59±0.31	9.59±0.27	9.57±0.27	9.58±0.27
Spindle frequency	11.53±0.11	11.51±0.12	11.52±0.12	11.38±0.08	11.40±0.08	11.39±0.08
EEG channel: C3A2						
Number of spindles	916.68±92.50	937.57±94.13	927.13±91.04	822.38±93.07	804.63±82.29	813.50±87.07
Spindle density	1.99±0.17	2.06±0.19	2.03±0.17	1.74±0.19	1.71±0.18	1.72±0.18
Spindle length	0.84±0.02	0.85±0.02	0.84±0.02	0.81±0.02	0.81±0.02	0.81±0.02
Spindle amplitude (μV)	9.51±0.23	9.44±0.29	9.48±0.26	8.99±0.21	8.92±0.21	8.96±0.21
Spindle frequency	11.47±0.12	11.46±0.12	11.46±0.12	11.29±0.08	11.32±0.08	11.31±0.08
EEG channel: P3A2						
Number of spindles	524.36±55.94	537.57±64.86	530.96±59.06	505.44±69.79	502.33±63.32	503.88±66.15
Spindle density	1.13±0.11	1.17±0.12	1.15±0.11	1.08±0.15	1.07±0.14	1.07±0.14
Spindle length	0.77±0.01	0.77±0.01	0.77±0.01	0.77±0.01	0.76±0.01	0.77±0.01
Spindle amplitude (μV)	8.60±0.26	8.41±0.25	8.51±0.25	8.10±0.19	8.03±0.19	8.07±0.19
Spindle frequency	11.39±0.11	11.37±0.12	11.38±0.11	11.21±0.08	11.24±0.08	11.22±0.08

Group mean ± SEM. DZ: dizygotic twins, MZ: monozygotic twins.
* Data were logarithm transformed prior to analysis.
** DZ and MZ means are not equal at the 5% level.

Supplementary Table S9: Individually adjusted frequency ranges. Genetic variance analysis, type of estimate applied (GCT: combined among- and within-twin pair component estimate, GWT: within-pair estimate) and intraclass correlation coefficients (ICCs) for slow spindle parameters in stage 2 sleep.

Variable	*P*	GWT vs GCT	ICC MZ	ICC DZ	ICC MZ cn	ICC DZ cn
EEG channel: Fp1A2						
Number of spindles	<.001	GWT	0.88(0.45, 0.12)	0.36(0.65, 0.19)	0.94(0.31, 0.08)	0.85(0.47, 0.14)
Spindle density	<.001	GWT	0.92(0.44, 0.12)	0.24(0.65, 0.19)	0.96(0.30, 0.08)	0.91(0.48, 0.14)
Spindle length	.002	GCT	0.94(0.48, 0.12)	0.40(0.63, 0.19)	0.98(0.39, 0.09)	0.92(0.48, 0.13)
Spindle amplitude	<.001	GWT	0.86(0.45, 0.12)	0.23(0.63, 0.18)	0.93(0.32, 0.09)	0.92(0.44, 0.13)
Spindle frequency	<.001	GWT	0.95(0.46, 0.12)	0.38(0.65, 0.19)	0.94(0.32, 0.08)	0.97(0.48, 0.12)
EEG channel: F3A2						
Number of spindles	<.001	GWT	0.91(0.47, 0.12)	0.43(0.62, 0.18)	0.92(0.33, 0.09)	0.88(0.49, 0.14)
Spindle density	.001	GCT	0.94(0.45, 0.13)	0.24(0.65, 0.19)	0.94(0.31, 0.09)	0.91(0.46, 0.13)
Spindle length	.002	GCT	0.96(0.45, 0.12)	0.42(0.65, 0.19)	0.96(0.32, 0.09)	0.92(0.48, 0.13)
Spindle amplitude*	-	-	0.88(0.46, 0.12)	0.19(0.65, 0.18)	0.91(0.34, 0.09)	0.88(0.48, 0.13)
Spindle frequency	<.001	GWT	0.94(0.43, 0.12)	0.43(0.62, 0.18)	0.93(0.32, 0.09)	0.96(0.48, 0.13)
EEG channel: C3A2						
Number of spindles	.003	GCT	0.92(0.46, 0.12)	0.39(0.65, 0.19)	0.92(0.32, 0.09)	0.86(0.50, 0.12)
Spindle density	<.001	GCT	0.94(0.49, 0.12)	0.20(0.69, 0.18)	0.94(0.32, 0.08)	0.92(0.49, 0.12)
Spindle length	<.001	GCT	0.93(0.52, 0.12)	0.33(0.69, 0.19)	0.95(0.39, 0.08)	0.83(0.46, 0.13)
Spindle amplitude	<.001	GWT	0.89(0.47, 0.12)	0.41(0.72, 0.17)	0.91(0.32, 0.09)	0.92(0.47, 0.13)
Spindle frequency	<.001	GWT	0.93(0.47, 0.12)	0.51(0.67, 0.19)	0.91(0.30, 0.09)	0.95(0.46, 0.13)
EEG channel: P3A2						
Number of spindles	<.001	GCT	0.96(0.53, 0.11)	0.22(0.69, 0.19)	0.93(0.34, 0.08)	0.88(0.48, 0.13)
Spindle density	<.001	GCT	0.96(0.53, 0.11)	0.04(0.68, 0.18)	0.94(0.36, 0.08)	0.91(0.48, 0.13)
Spindle length	<.001	GCT	0.94(0.52, 0.12)	-0.19(0.66, 0.19)	0.90(0.37, 0.08)	0.81(0.47, 0.13)
Spindle amplitude	.005	GWT	0.84(0.46, 0.13)	0.50(0.61, 0.17)	0.88(0.31, 0.09)	0.79(0.47, 0.12)
Spindle frequency	<.001	GWT	0.94(0.44, 0.12)	0.41(0.64, 0.18)	0.90(0.34, 0.09)	0.95(0.46, 0.12)

Results of genetic variance analysis, type of estimate applied (GCT: combined among- and within-twin pair component estimate, GWT: within-pair estimate) and intraclass correlation coefficients (ICCs). ICC MZ: ICCs of monozygotic (MZ) twins, ICC DZ: ICCs of dizygotic (DZ) twins, ICC MZ cn: ICCs of consecutive nights for each subject in MZ group, ICC DZ cn: ICCs of consecutive nights for each subject in DZ group. ICC results include: original sample ICC (upper percentile of bootstrapped data, median of bootstrapped data).
* Analysis of variance not applicable (significant differences between the means in DZ and MZ twin set).

Supplementary Table S10: Individually adjusted frequency ranges. Slow spindles in slow wave sleep. Parameters Averaged Over Pairs.

	DZ n = 14			MZ n = 32		
	Night 2	*Night 3*	*2 nights mean*	*Night 2*	*Night 3*	*2 nights mean*
EEG channel: Fp1A2						
Number of spindles	849.39±101.39	892.39±84.21	870.89±90.32	649.84±88.15	581.89±78.76	615.87±82.86
Spindle density	4.48±0.38	4.60±0.42	4.54±0.40	3.84±0.45	3.67±0.43	3.75±0.43
Spindle length	0.96±0.04	0.94±0.03	0.95±0.03	0.93±0.04	0.92±0.04	0.93±0.04
Spindle amplitude (µV)	8.60±0.23	8.47±0.24	8.53±0.23	8.38±0.29	8.35±0.28	8.37±0.28
Spindle frequency	10.98±0.15	10.97±0.15	10.98±0.15	10.94±0.11	10.99±0.11	10.96±0.11
EEG channel: F3A2						
Number of spindles**	968.79±104.83	1038.93±79.72	1003.86±88.05	691.06±92.62	613.11±82.77	652.09±86.95
Spindle density**	5.11±0.36	5.35±0.39	5.23±0.37	4.11±0.49	3.87±0.48	3.99±0.48
Spindle length	0.98±0.04	0.98±0.03	0.98±0.03	0.96±0.05	0.93±0.05	0.94±0.05
Spindle amplitude (µV)	10.06±0.20	9.95±0.30	10.01±0.24	9.36±0.30	9.32±0.31	9.34±0.30
Spindle frequency	11.01±0.16	11.01±0.17	11.01±0.16	10.95±0.11	11.01±0.11	10.98±0.11
EEG channel: C3A2						
Number of spindles	621.43±97.82	657.29±71.33	639.36±79.60	491.67±83.13	425.22±72.34	458.45±77.07
Spindle density	3.25±0.45	3.41±0.44	3.33±0.44	2.88±0.45	2.68±0.44	2.78±0.44
Spindle length	0.86±0.03	0.86±0.03	0.86±0.03	0.86±0.03	0.85±0.04	0.85±0.04
Spindle amplitude (µV)	8.97±0.18	8.90±0.24	8.93±0.20	8.67±0.22	8.56±0.25	8.62±0.23
Spindle frequency	10.95±0.16	10.94±0.16	10.95±0.16	10.89±0.11	10.92±0.11	10.90±0.11
EEG channel: P3A2						
Number of spindles*	271.21±56.71	272.46±50.09	271.83±52.43	254.94±58.28	212.31±44.82	233.62±51.21
Spindle density	1.43±0.32	1.47±0.32	1.45±0.32	1.43±0.30	1.32±0.29	1.37±0.29
Spindle length	0.77±0.02	0.76±0.02	0.77±0.02	0.78±0.03	0.77±0.02	0.77±0.02
Spindle amplitude (µV)	8.05±0.24	7.82±0.22	7.94±0.22	7.70±0.18	7.65±0.18	7.67±0.18
Spindle frequency	10.81±0.15	10.82±0.17	10.81±0.16	10.76±0.10	10.81±0.10	10.78±0.10

Group mean ± SEM. DZ: dizygotic twins, MZ: monozygotic twins.
* Data were logarithm transformed prior to analysis.
** DZ and MZ means are not equal at the 5% level.

Supplementary Table S11: Individually adjusted frequency ranges. Genetic variance analysis, type of estimate applied (GCT: combined among- and within-twin pair component estimate, GWT: within-pair estimate) and intraclass correlation coefficients (ICCs) for slow spindle parameters in slow wave sleep.

Variable	*P*	GWT vs GCT	ICC MZ	ICC DZ	ICC MZ cn	ICC DZ cn
EEG channel: Fp1A2						
Number of spindles	<.001	GWT	0.90(0.46, 0.12)	0.23(0.72, 0.18)	0.92(0.34, 0.08)	0.85(0.51, 0.14)
Spindle density	.001	GCT	0.92(0.45, 0.13)	0.18(0.66, 0.18)	0.95(0.32, 0.09)	0.95(0.47, 0.13)
Spindle length	<.001	GCT	0.97(0.48, 0.11)	0.33(0.75, 0.17)	0.95(0.35, 0.09)	0.94(0.48, 0.12)
Spindle amplitude	<.001	GCT	0.91(0.43, 0.12)	0.18(0.65, 0.20)	0.93(0.33, 0.08)	0.88(0.45, 0.13)
Spindle frequency	.066	GWT	0.90(0.47, 0.12)	0.81(0.69, 0.19)	0.94(0.32, 0.08)	0.97(0.43, 0.13)
EEG channel: F3A2						
Number of spindles*	-	-	0.94(0.46, 0.12)	0.14(0.61, 0.18)	0.92(0.35, 0.09)	0.76(0.46, 0.13)
Spindle density*	-	-	0.96(0.47, 0.12)	0.05(0.62, 0.18)	0.96(0.32, 0.08)	0.91(0.46, 0.13)
Spindle length	<.001	GCT	0.98(0.57, 0.11)	0.45(0.69, 0.18)	0.92(0.42, 0.08)	0.89(0.49, 0.13)
Spindle amplitude	<.001	GCT	0.89(0.45, 0.13)	0.16(0.65, 0.20)	0.91(0.34, 0.09)	0.78(0.45, 0.13)
Spindle frequency	.052	GWT	0.91(0.45, 0.12)	0.81(0.66, 0.19)	0.93(0.33, 0.08)	0.97(0.48, 0.13)
EEG channel: C3A2						
Number of spindles	<.001	GCT	0.94(0.47, 0.13)	0.20(0.69, 0.18)	0.92(0.33, 0.08)	0.73(0.51, 0.13)
Spindle density	<.001	GCT	0.94(0.49, 0.12)	0.41(0.64, 0.19)	0.95(0.34, 0.09)	0.89(0.48, 0.13)
Spindle length	<.001	GCT	0.91(0.49, 0.11)	0.64(0.70, 0.18)	0.95(0.37, 0.08)	0.90(0.51, 0.13)
Spindle amplitude	<.001	GCT	0.89(0.42, 0.12)	0.31(0.64, 0.18)	0.85(0.31, 0.08)	0.81(0.46, 0.14)
Spindle frequency	.073	GWT	0.88(0.44, 0.12)	0.78(0.62, 0.20)	0.91(0.33, 0.09)	0.97(0.46, 0.13)
EEG channel: P3A2						
Number of spindles	.005	GCT	0.88(0.42, 0.12)	0.45(0.66, 0.19)	0.89(0.34, 0.08)	0.87(0.46, 0.13)
Spindle density	.030	GWT	0.90(0.53, 0.13)	0.64(0.70, 0.20)	0.93(0.36, 0.09)	0.95(0.50, 0.13)
Spindle length	<.001	GCT	0.88(0.57, 0.11)	0.46(0.70, 0.20)	0.88(0.39, 0.08)	0.90(0.47, 0.13)
Spindle amplitude	.004	GWT	0.83(0.44, 0.12)	0.41(0.63, 0.19)	0.79(0.33, 0.09)	0.73(0.48, 0.12)
Spindle frequency	.049	GWT	0.82(0.45, 0.12)	0.69(0.63, 0.19)	0.88(0.33, 0.08)	0.93(0.48, 0.13)

Results of genetic variance analysis, type of estimate applied (GCT: combined among- and within-twin pair component estimate, GWT: within-pair estimate) and intraclass correlation coefficients (ICCs). ICC MZ: ICCs of monozygotic (MZ) twins, ICC DZ: ICCs of dizygotic (DZ) twins, ICC MZ cn: ICCs of consecutive nights for each subject in MZ group, ICC DZ cn: ICCs of consecutive nights for each subject in DZ group. ICC results include: original sample ICC (upper percentile of bootstrapped data, median of bootstrapped data).
* Analysis of variance not applicable (significant differences between the means in DZ and MZ twin set).

Supplementary Table S12: Fixed frequency ranges. Slow spindle: 11-12.9 Hz. Fast spindle: 13.1-16 Hz. Fast spindles in stage 2 sleep. Parameters Averaged Over Pairs.

	DZ n = 14			MZ n = 32		
	Night 2	*Night 3*	*2 nights mean*	*Night 2*	*Night 3*	*2 nights mean*
EEG channel: Fp1A2						
Number of spindles	206.25±45.83	198.32±53.12	202.29±49.01	152.25±25.28	155.64±29.45	153.95±27.02
Spindle density	0.43±0.09	0.42±0.10	0.42±0.10	0.31±0.05	0.32±0.06	0.31±0.05
Spindle length	0.68±0.01	0.67±0.01	0.68±0.01	0.66±0.01	0.66±0.01	0.66±0.01
Spindle amplitude (µV)	8.49±0.25	8.25±0.26	8.37±0.25	8.17±0.25	8.19±0.22	8.18±0.23
Spindle frequency	13.84±0.05	13.78±0.04	13.81±0.04	13.79±0.02	13.76±0.02	13.77±0.02
EEG channel: F3A2						
Number of spindles	455.11±86.00	445.96±91.62	450.54±87.98	388.14±55.85	399.63±61.26	393.88±57.67
Spindle density	0.95±0.16	0.96±0.17	0.95±0.17	0.80±0.11	0.82±0.12	0.81±0.12
Spindle length	0.73±0.01	0.72±0.01	0.72±0.01	0.70±0.01	0.71±0.01	0.70±0.01
Spindle amplitude (µV)	10.65±0.22	10.43±0.30	10.54±0.26	9.83±0.30	9.80±0.29	9.81±0.29
Spindle frequency	13.81±0.03	13.81±0.03	13.81±0.03	13.77±0.02	13.79±0.02	13.78±0.02
EEG channel: C3A2						
Number of spindles	893.00±129.26	890.71±121.65	891.86±123.01	888.39±100.77	867.58±99.52	877.98±98.71
Spindle density	1.88±0.24	1.93±0.23	1.90±0.23	1.86±0.20	1.81±0.20	1.83±0.20
Spindle length	0.80±0.02	0.80±0.02	0.80±0.02	0.78±0.01	0.78±0.01	0.78±0.01
Spindle amplitude (µV)	10.83±0.27	10.75±0.29	10.79±0.27	10.39±0.27	10.23±0.27	10.31±0.27
Spindle frequency	13.88±0.05	13.89±0.06	13.89±0.05	13.81±0.04	13.83±0.04	13.82±0.04
EEG channel: P3A2						
Number of spindles	951.25±157.08	945.61±143.09	948.43±148.00	921.41±108.11	905.50±109.30	913.45±107.03
Spindle density	2.00±0.30	2.04±0.28	2.02±0.29	1.94±0.22	1.91±0.22	1.93±0.22
Spindle length	0.82±0.02	0.83±0.02	0.82±0.02	0.80±0.01	0.79±0.01	0.79±0.01
Spindle amplitude (µV)	10.26±0.44	9.95±0.35	10.10±0.37	9.56±0.29	9.43±0.29	9.50±0.29
Spindle frequency	13.86±0.06	13.87±0.07	13.87±0.07	13.78±0.04	13.79±0.05	13.79±0.04

Group mean ± SEM. DZ: dizygotic twins, MZ: monozygotic twins.

Supplementary Table S13: Fixed frequency ranges. Slow spindle: 11-12.9 Hz. Fast spindle: 13.1-16 Hz. Genetic variance analysis, type of estimate applied (GCT: combined among- and within-twin pair component estimate, GWT: within-pair estimate) and intraclass correlation coefficients (ICCs) for fast spindle parameters in stage 2 sleep.

Variable	*P*	GWT vs GCT	ICC MZ	ICC DZ	ICC MZ cn	ICC DZ cn
EEG channel: Fp1A2						
Number of spindles	.050	GWT	0.82(0.56, 0.12)	0.76(0.77, 0.18)	0.85(0.36, 0.09)	0.94(0.51, 0.13)
Spindle density	.029	GWT	0.83(0.58, 0.12)	0.74(0.68, 0.18)	0.85(0.39, 0.09)	0.95(0.54, 0.13)
Spindle length	.318	GWT	0.68(0.43, 0.11)	0.59(0.69, 0.17)	0.56(0.32, 0.09)	0.72(0.42, 0.13)
Spindle amplitude	<.001	GWT	0.85(0.47, 0.12)	0.20(0.62, 0.19)	0.90(0.34, 0.09)	0.87(0.44, 0.13)
Spindle frequency	.406	GCT	0.58(0.40, 0.12)	0.15(0.71, 0.17)	0.36(0.31, 0.08)	0.77(0.51, 0.12)
EEG channel: F3A2						
Number of spindles	.505	GWT	0.78(0.48, 0.12)	0.79(0.74, 0.18)	0.87(0.32, 0.08)	0.93(0.54, 0.13)
Spindle density	.396	GWT	0.82(0.46, 0.12)	0.79(0.71, 0.16)	0.85(0.33, 0.08)	0.94(0.52, 0.13)
Spindle length	.202	GWT	0.74(0.50, 0.12)	0.60(0.63, 0.19)	0.72(0.31, 0.08)	0.86(0.49, 0.12)
Spindle amplitude	.005	GCT	0.87(0.45, 0.12)	0.15(0.64, 0.20)	0.88(0.34, 0.08)	0.75(0.47, 0.13)
Spindle frequency	.015	GWT	0.76(0.43, 0.11)	0.37(0.63, 0.19)	0.62(0.33, 0.08)	0.87(0.47, 0.13)
EEG channel: C3A2						
Number of spindles	.333	GWT	0.81(0.44, 0.12)	0.70(0.66, 0.18)	0.92(0.32, 0.08)	0.85(0.48, 0.13)
Spindle density	.081	GWT	0.87(0.45, 0.12)	0.66(0.65, 0.18)	0.90(0.33, 0.09)	0.83(0.51, 0.13)
Spindle length	.007	GWT	0.85(0.41, 0.12)	0.54(0.64, 0.19)	0.90(0.33, 0.08)	0.87(0.49, 0.13)
Spindle amplitude	.003	GWT	0.87(0.40, 0.12)	0.33(0.61, 0.18)	0.90(0.34, 0.08)	0.84(0.48, 0.14)
Spindle frequency	<.001	GWT	0.91(0.43, 0.12)	0.61(0.67, 0.18)	0.95(0.34, 0.08)	0.94(0.48, 0.13)
EEG channel: P3A2						
Number of spindles	.097	GWT	0.87(0.49, 0.12)	0.74(0.61, 0.20)	0.90(0.33, 0.09)	0.87(0.48, 0.13)
Spindle density	.021	GWT	0.88(0.43, 0.12)	0.67(0.65, 0.19)	0.89(0.36, 0.09)	0.87(0.45, 0.13)
Spindle length	.005	GWT	0.82(0.45, 0.12)	0.55(0.67, 0.18)	0.88(0.34, 0.09)	0.86(0.49, 0.13)
Spindle amplitude	.065	GWT	0.81(0.45, 0.12)	0.58(0.66, 0.18)	0.87(0.32, 0.08)	0.64(0.47, 0.13)
Spindle frequency	.001	GWT	0.90(0.46, 0.12)	0.68(0.64, 0.20)	0.95(0.34, 0.09)	0.97(0.50, 0.13)

Results of genetic variance analysis, type of estimate applied (GCT: combined among- and within-twin pair component estimate, GWT: within-pair estimate) and intraclass correlation coefficients (ICCs). ICC MZ: ICCs of monozygotic (MZ) twins, ICC DZ: ICCs of dizygotic (DZ) twins, ICC MZ cn: ICCs of consecutive nights for each subject in MZ group, ICC DZ cn: ICCs of consecutive nights for each subject in DZ group. ICC results include: original sample ICC (upper percentile of bootstrapped data, median of bootstrapped data).

Supplementary Table S14: Fixed frequency ranges. Slow spindle: 11-12.9 Hz. Fast spindle: 13.1-16 Hz. Slow spindles in stage 2 sleep. Parameters Averaged Over Pairs.

	DZ n = 14			MZ n = 32		
	Night 2	*Night 3*	*2 nights mean*	*Night 2*	*Night 3*	*2 nights mean*
EEG channel: Fp1A2						
Number of spindles	1509.89±137.17	1454.57±117.46	1482.23±125.25	1374.11±96.64	1400.81±90.57	1387.46±92.46
Spindle density	3.26±0.27	3.23±0.25	3.25±0.26	2.90±0.19	2.96±0.19	2.93±0.19
Spindle length	0.98±0.03	0.98±0.03	0.98±0.03	0.92±0.02	0.92±0.02	0.92±0.02
Spindle amplitude (μV)	8.74±0.23	8.60±0.26	8.67±0.24	8.44±0.24	8.43±0.22	8.44±0.23
Spindle frequency	11.45±0.05	11.45±0.05	11.45±0.05	11.52±0.04	11.52±0.04	11.52±0.04
EEG channel: F3A2						
Number of spindles	1550.82±127.81	1509.64±112.45	1530.23±118.24	1412.66±91.22	1407.61±84.90	1410.13±87.00
Spindle density	3.35±0.25	3.36±0.24	3.36±0.24	2.99±0.18	2.98±0.18	2.98±0.18
Spindle length	0.99±0.03	0.99±0.03	0.99±0.03	0.91±0.02	0.91±0.02	0.91±0.02
Spindle amplitude (μV)	10.63±0.23	10.54±0.34	10.58±0.28	9.85±0.27	9.79±0.25	9.82±0.26
Spindle frequency	11.52±0.05	11.51±0.06	11.51±0.05	11.57±0.05	11.57±0.04	11.57±0.04
EEG channel: C3A2						
Number of spindles	1221.50±108.34	1176.57±96.65	1199.04±101.51	1064.33±92.24	1061.64±85.52	1062.98±87.37
Spindle density	2.66±0.24	2.63±0.22	2.65±0.23	2.26±0.20	2.25±0.18	2.26±0.19
Spindle length	0.87±0.02	0.87±0.02	0.87±0.02	0.84±0.02	0.84±0.02	0.84±0.02
Spindle amplitude (μV)	9.73±0.23	9.65±0.27	9.69±0.24	9.46±0.21	9.34±0.21	9.40±0.21
Spindle frequency	11.51±0.07	11.50±0.08	11.51±0.07	11.55±0.06	11.55±0.06	11.55±0.06
EEG channel: P3A2						
Number of spindles	873.68±100.60	831.21±85.53	852.45±91.56	747.05±84.13	744.91±82.31	745.98±82.35
Spindle density	1.90±0.23	1.86±0.20	1.88±0.21	1.61±0.19	1.58±0.18	1.59±0.18
Spindle length	0.81±0.01	0.81±0.01	0.81±0.01	0.80±0.02	0.79±0.01	0.80±0.01
Spindle amplitude (μV)	9.07±0.27	8.88±0.23	8.98±0.24	8.69±0.20	8.52±0.19	8.61±0.19
Spindle frequency	11.45±0.10	11.44±0.11	11.45±0.10	11.50±0.06	11.49±0.06	11.50±0.06

Group mean ± SEM. DZ: dizygotic twins, MZ: monozygotic twins.

Supplementary Table S15: Fixed frequency ranges. Slow spindle: 11-12.9 Hz. Fast spindle: 13.1-16 Hz. Genetic variance analysis, type of estimate applied (GCT: combined among- and within-twin pair component estimate, GWT: within-pair estimate) and intraclass correlation coefficients (ICCs) for slow spindle parameters in stage 2 sleep.

Variable	*P*	GWT vs GCT	ICC MZ	ICC DZ	ICC MZ cn	ICC DZ cn
EEG channel: Fp1A2						
Number of spindles	<.001	GWT	0.90(0.45, 0.12)	0.47(0.68, 0.19)	0.93(0.32, 0.09)	0.91(0.46, 0.12)
Spindle density	<.001	GWT	0.94(0.44, 0.13)	0.49(0.66, 0.19)	0.97(0.33, 0.08)	0.96(0.49, 0.13)
Spindle length	<.001	GWT	0.91(0.47, 0.11)	0.68(0.65, 0.20)	0.98(0.32, 0.08)	0.94(0.47, 0.13)
Spindle amplitude	<.001	GWT	0.84(0.44, 0.12)	0.18(0.64, 0.18)	0.92(0.32, 0.08)	0.90(0.46, 0.12)
Spindle frequency	<.001	GWT	0.94(0.43, 0.12)	0.33(0.67, 0.18)	0.97(0.31, 0.08)	0.95(0.48, 0.13)
EEG channel: F3A2						
Number of spindles	<.001	GWT	0.89(0.46, 0.12)	0.57(0.64, 0.19)	0.91(0.33, 0.08)	0.91(0.49, 0.14)
Spindle density	<.001	GWT	0.93(0.44, 0.12)	0.55(0.62, 0.18)	0.96(0.31, 0.08)	0.93(0.46, 0.13)
Spindle length	.001	GWT	0.93(0.51, 0.12)	0.79(0.64, 0.19)	0.97(0.32, 0.08)	0.95(0.50, 0.13)
Spindle amplitude	<.001	GWT	0.86(0.44, 0.12)	0.13(0.64, 0.18)	0.90(0.31, 0.08)	0.87(0.43, 0.12)
Spindle frequency	<.001	GWT	0.96(0.45, 0.13)	0.49(0.61, 0.19)	0.98(0.36, 0.09)	0.96(0.47, 0.12)
EEG channel: C3A2						
Number of spindles	<.001	GWT	0.91(0.46, 0.12)	0.49(0.65, 0.18)	0.88(0.31, 0.09)	0.92(0.48, 0.13)
Spindle density	<.001	GWT	0.94(0.45, 0.12)	0.48(0.69, 0.19)	0.95(0.32, 0.08)	0.95(0.49, 0.13)
Spindle length	.018	GWT	0.89(0.48, 0.13)	0.55(0.61, 0.19)	0.96(0.32, 0.09)	0.83(0.45, 0.13)
Spindle amplitude	<.001	GWT	0.89(0.46, 0.11)	0.38(0.65, 0.18)	0.91(0.31, 0.09)	0.90(0.51, 0.13)
Spindle frequency	.003	GWT	0.93(0.41, 0.12)	0.74(0.62, 0.17)	0.97(0.32, 0.08)	0.96(0.47, 0.13)
EEG channel: P3A2						
Number of spindles	.001	GWT	0.90(0.45, 0.12)	0.49(0.63, 0.18)	0.93(0.32, 0.09)	0.91(0.51, 0.12)
Spindle density	.002	GWT	0.89(0.48, 0.12)	0.51(0.61, 0.18)	0.97(0.31, 0.09)	0.94(0.46, 0.14)
Spindle length	.010	GCT	0.82(0.49, 0.11)	0.28(0.67, 0.18)	0.93(0.37, 0.08)	0.82(0.46, 0.13)
Spindle amplitude	.022	GWT	0.83(0.45, 0.12)	0.51(0.64, 0.17)	0.84(0.35, 0.09)	0.71(0.50, 0.13)
Spindle frequency	.003	GWT	0.87(0.45, 0.12)	0.68(0.65, 0.18)	0.95(0.32, 0.09)	0.96(0.50, 0.14)

Results of genetic variance analysis, type of estimate applied (GCT: combined among- and within-twin pair component estimate, GWT: within-pair estimate) and intraclass correlation coefficients (ICCs). ICC MZ: ICCs of monozygotic (MZ) twins, ICC DZ: ICCs of dizygotic (DZ) twins, ICC MZ cn: ICCs of consecutive nights for each subject in MZ group, ICC DZ cn: ICCs of consecutive nights for each subject in DZ group. ICC results include: original sample ICC (upper percentile of bootstrapped data, median of bootstrapped data).

Supplementary Table S16: Fixed frequency ranges. Slow spindle: 11-12.9 Hz. Fast spindle: 13.1-16 Hz. Fast spindles in slow wave sleep. Parameters Averaged Over Pairs.

	DZ n = 14			MZ n = 32		
	Night 2	*Night 3*	*2 nights mean*	*Night 2*	*Night 3*	*2 nights mean*
EEG channel: Fp1A2						
Number of spindles*	10.89±2.92	13.96±3.91	12.43±3.19	9.88±3.60	8.70±2.65	9.29±3.10
Spindle density***	0.07±0.03	0.08±0.03	0.08±0.03	0.06±0.02	0.06±0.02	0.06±0.02
Spindle length	0.61±0.01	0.60±0.01	0.61±0.01	0.60±0.01	0.61±0.01	0.61±0.01
Spindle amplitude (µV)	8.63±0.23	8.94±0.33	8.79±0.22	8.96±0.39	8.82±0.43	9.01±0.45
Spindle frequency	13.39±0.20	13.62±0.04	13.51±0.10	13.59±0.03	13.59±0.04	13.58±0.02
EEG channel: F3A2						
Number of spindles	47.89±13.40	49.46±12.97	48.68±12.52	36.66±8.40	34.38±7.23	35.52±7.48
Spindle density	0.29±0.10	0.29±0.11	0.29±0.10	0.21±0.05	0.22±0.04	0.22±0.04
Spindle length	0.63±0.01	0.63±0.01	0.63±0.01	0.62±0.01	0.62±0.01	0.62±0.01
Spindle amplitude (µV)	11.02±0.18	10.61±0.26	10.81±0.19	10.37±0.35	10.01±0.37	10.19±0.35
Spindle frequency	13.63±0.04	13.69±0.03	13.66±0.03	13.60±0.04	13.67±0.03	13.64±0.03
EEG channel: C3A2						
Number of spindles	167.32±33.43	181.57±32.49	174.45±30.41	137.47±23.62	127.92±23.70	132.70±22.97
Spindle density	0.91±0.17	0.94±0.17	0.92±0.16	0.79±0.14	0.76±0.12	0.77±0.13
Spindle length	0.67±0.01	0.68±0.01	0.67±0.01	0.66±0.01	0.67±0.01	0.66±0.01
Spindle amplitude (µV)	10.69±0.21	10.73±0.22	10.71±0.19	10.42±0.25	10.22±0.31	10.32±0.28
Spindle frequency	13.80±0.05	13.83±0.06	13.81±0.06	13.76±0.04	13.78±0.04	13.77±0.04
EEG channel: P3A2						
Number of spindles	213.36±46.44	219.86±38.73	216.61±40.74	160.17±28.72	143.27±27.14	151.72±27.38
Spindle density	1.12±0.22	1.12±0.19	1.12±0.20	0.90±0.16	0.85±0.15	0.88±0.15
Spindle length	0.70±0.01	0.70±0.01	0.70±0.01	0.68±0.01	0.67±0.01	0.68±0.01
Spindle amplitude (µV)	9.97±0.33	9.68±0.25	9.82±0.27	9.42±0.26	9.27±0.29	9.35±0.28
Spindle frequency	13.83±0.07	13.84±0.07	13.83±0.07	13.75±0.04	13.77±0.05	13.76±0.04

Group mean ± SEM. DZ: dizygotic twins, MZ: monozygotic twins.
* Data were logarithm transformed prior to analysis.
** DZ and MZ means are not equal at the 5% level.
*** Data were logarithm transformed prior to analysis and DZ and MZ means were not equal at the 5% level.

Supplementary Table S17: Fixed frequency ranges. Slow spindle: 11-12.9 Hz. Fast spindle: 13.1-16 Hz. Genetic variance analysis, type of estimate applied (GCT: combined among- and within-twin pair component estimate, GWT: within-pair estimate) and intraclass correlation coefficients (ICCs) for fast spindle parameters in slow wave sleep.

Variable	*P*	GWT vs GCT	ICC MZ	ICC DZ	ICC MZ cn	ICC DZ cn
EEG channel: Fp1A2						
Number of spindles	<.001	GWT	0.83(0.45, 0.11)	0.24(0.65, 0.19)	0.79(0.32, 0.08)	0.82(0.49, 0.13)
Spindle density	-	-	0.74(0.59, 0.12)	0.82(0.84, 0.17)	0.89(0.39, 0.08)	0.86(0.57, 0.12)
Spindle length	.362	GWT	-0.08(0.66, 0.18)	-0.08(0.73, 0.23)	-0.03(0.48, 0.10)	0.12(0.53, 0.15)
Spindle amplitude	<.001	GCT	0.77(0.69, 0.16)	0.32(0.77, 0.22)	0.78(0.42, 0.11)	0.33(0.52, 0.13)
Spindle frequency	.350	GCT	0.12(0.63, 0.17)	-0.12(0.92, 0.14)	-0.10(0.41, 0.11)	0.08(0.88, 0.10)
EEG channel: F3A2						
Number of spindles	.780	GWT	0.58(0.53, 0.12)	0.74(0.73, 0.18)	0.70(0.43, 0.09)	0.77(0.52, 0.12)
Spindle density	.988	GCT	0.60(0.50, 0.13)	0.91(0.83, 0.15)	0.76(0.37, 0.09)	0.92(0.59, 0.12)
Spindle length	.017	GWT	0.67(0.54, 0.14)	-0.04(0.65, 0.19)	0.42(0.36, 0.10)	0.48(0.45, 0.13)
Spindle amplitude	<.001	GCT	0.67(0.53, 0.14)	-0.39(0.66, 0.19)	0.72(0.37, 0.09)	0.60(0.46, 0.12)
Spindle frequency	.627	GWT	0.51(0.51, 0.13)	0.38(0.63, 0.18)	0.25(0.39, 0.10)	0.56(0.46, 0.13)
EEG channel: C3A2						
Number of spindles	0.085	GWT	0.74(0.51, 0.13)	0.48(0.67, 0.18)	0.81(0.33, 0.08)	0.61(0.49, 0.12)
Spindle density	0.189	GWT	0.75(0.50, 0.12)	0.56(0.69, 0.19)	0.85(0.35, 0.08)	0.73(0.50, 0.13)
Spindle length	0.005	GWT	0.84(0.46, 0.12)	0.44(0.66, 0.19)	0.71(0.31, 0.09)	0.67(0.49, 0.13)
Spindle amplitude	<.001	GCT	0.74(0.45, 0.12)	-0.01(0.71, 0.18)	0.81(0.33, 0.09)	0.66(0.48, 0.14)
Spindle frequency	<.029	GWT	0.84(0.47, 0.12)	0.62(0.62, 0.19)	0.81(0.32, 0.09)	0.85(0.50, 0.13)
EEG channel: P3A2						
Number of spindles	.169	GWT	0.58(0.51, 0.12)	0.41(0.69, 0.19)	0.90(0.37, 0.09)	0.71(0.52, 0.13)
Spindle density	.089	GWT	0.75(0.51, 0.13)	0.47(0.68, 0.19)	0.89(0.34, 0.09)	0.71(0.52, 0.13)
Spindle length	.037	GWT	0.76(0.45, 0.13)	0.53(0.66, 0.18)	0.72(0.32, 0.09)	0.72(0.45, 0.12)
Spindle amplitude	.046	GWT	0.80(0.49, 0.13)	0.32(0.67, 0.19)	0.80(0.35, 0.09)	0.55(0.49, 0.12)
Spindle frequency	.083	GWT	0.82(0.47, 0.13)	0.73(0.64, 0.19)	0.87(0.32, 0.09)	0.95(0.51, 0.13)

Results of genetic variance analysis, type of estimate applied (GCT: combined among- and within-twin pair component estimate, GWT: within-pair estimate) and intraclass correlation coefficients (ICCs). ICC MZ: ICCs of monozygotic (MZ) twins, ICC DZ: ICCs of dizygotic (DZ) twins, ICC MZ cn: ICCs of consecutive nights for each subject in MZ group, ICC DZ cn: ICCs of consecutive nights for each subject in DZ group. ICC results include: original sample ICC (upper percentile of bootstrapped data, median of bootstrapped data).

Supplementary Table S18: Fixed frequency ranges. Slow spindle: 11-12.9 Hz. Fast spindle: 13.1-16 Hz. Slow spindles in slow wave sleep. Parameters Averaged Over Pairs.

	DZ n = 14			MZ n = 32		
	Night 2	*Night 3*	*2 nights mean*	*Night 2*	*Night 3*	*2 nights mean*
EEG channel: Fp1A2						
Number of spindles**	695.54±88.08	719.89±85.26	707.71±84.87	479.91±63.60	463.14±60.29	471.52±61.58
Spindle density	3.62±0.34	3.59±0.35	3.61±0.34	2.88±0.33	2.94±0.33	2.91±0.33
Spindle length	0.91±0.02	0.90±0.02	0.91±0.02	0.85±0.02	0.85±0.02	0.85±0.02
Spindle amplitude (µV)	8.99±0.22	8.90±0.22	8.94±0.21	8.91±0.33	8.81±0.33	8.86±0.33
Spindle frequency	11.21±0.08	11.22±0.08	11.22±0.08	11.28±0.06	11.29±0.06	11.28±0.06
EEG channel: F3A2						
Number of spindles**	816.43±97.61	877.96±96.28	847.20±93.99	532.17±66.12	507.00±63.48	519.59±64.18
Spindle density	4.24±0.33	4.36±0.34	4.30±0.33	3.22±0.36	3.22±0.37	3.22±0.36
Spindle length	0.94±0.02	0.93±0.02	0.94±0.02	0.87±0.02	0.86±0.02	0.87±0.02
Spindle amplitude (µV)	10.53±0.16	10.43±0.24	10.48±0.19	10.00±0.36	9.86±0.34	9.93±0.35
Spindle frequency	11.29±0.09	11.28±0.09	11.29±0.09	11.34±0.07	11.36±0.07	11.35±0.07
EEG channel: C3A2						
Number of spindles	514.39±79.81	543.68±70.43	529.04±71.42	370.08±56.72	348.31±50.60	359.20±53.03
Spindle density	2.61±0.26	2.66±0.26	2.63±0.25	2.23±0.33	2.21±0.31	2.22±0.31
Spindle length	0.82±0.01	0.82±0.01	0.82±0.01	0.80±0.02	0.79±0.02	0.79±0.02
Spindle amplitude (µV)	9.51±0.22	9.41±0.22	9.46±0.21	9.36±0.25	9.20±0.27	9.28±0.26
Spindle frequency	11.32±0.09	11.31±0.09	11.32±0.09	11.33±0.07	11.37±0.08	11.35±0.07
EEG channel: P3A2						
Number of spindles*	216.75±42.97	209.57±36.49	213.16±39.04	175.38±31.68	155.42±26.15	165.40±28.73
Spindle density*	1.08±0.17	1.04±0.16	1.06±0.16	0.99±0.17	0.96±0.16	0.98±0.17
Spindle length	0.74±0.01	0.75±0.01	0.75±0.01	0.74±0.01	0.73±0.01	0.73±0.01
Spindle amplitude (µV)	8.62±0.23	8.40±0.20	8.51±0.21	8.40±0.22	8.29±0.21	8.35±0.21
Spindle frequency	11.37±0.10	11.38±0.11	11.38±0.11	11.36±0.08	11.37±0.09	11.36±0.08

Group mean ± SEM. DZ: dizygotic twins, MZ: monozygotic twins.
* Data were logarithm transformed prior to analysis.
** DZ and MZ means are not equal at the 5% level.

Supplementary Table S19: Fixed frequency ranges. Slow spindle: 11-12.9 Hz. Fast spindle: 13.1-16 Hz. Genetic variance analysis, type of estimate applied (GCT: combined among- and within-twin pair component estimate, GWT: within-pair estimate) and intraclass correlation coefficients (ICCs) for slow spindle parameters in slow wave sleep.

Variable	*P*	GWT vs GCT	ICC MZ	ICC DZ	ICC MZ cn	ICC DZ cn
EEG channel: Fp1A2						
Number of spindles	-	-	0.89(0.47, 0.12)	0.35(0.66, 0.18)	0.94(0.35, 0.09)	0.89(0.50, 0.13)
Spindle density	<.001	GWT	0.93(0.47, 0.12)	0.20(0.63, 0.18)	0.96(0.32, 0.08)	0.97(0.51, 0.12)
Spindle length	<.001	GWT	0.92(0.48, 0.12)	0.21(0.70, 0.16)	0.95(0.32, 0.09)	0.96(0.51, 0.12)
Spindle amplitude	<.001	GCT	0.94(0.48, 0.13)	0.01(0.64, 0.18)	0.95(0.34, 0.09)	0.91(0.46, 0.13)
Spindle frequency	<.001	GWT	0.93(0.43, 0.12)	0.65(0.66, 0.18)	0.97(0.32, 0.09)	0.97(0.49, 0.12)
EEG channel: F3A2						
Number of spindles	-	-	0.90(0.44, 0.12)	0.46(0.63, 0.18)	0.93(0.33, 0.09)	0.83(0.47, 0.12)
Spindle density	.008	GCT	0.94(0.48, 0.12)	0.18(0.64, 0.18)	0.97(0.32, 0.09)	0.95(0.46, 0.13)
Spindle length	<.001	GWT	0.93(0.45, 0.12)	0.31(0.63, 0.17)	0.93(0.32, 0.09)	0.91(0.47, 0.12)
Spindle amplitude	<.001	GCT	0.93(0.45, 0.13)	-0.19(0.67, 0.19)	0.93(0.30, 0.09)	0.76(0.48, 0.12)
Spindle frequency	<.001	GWT	0.97(0.46, 0.12)	0.68(0.69, 0.19)	0.97(0.34, 0.08)	0.96(0.47, 0.14)
EEG channel: C3A2						
Number of spindles	<.001	GWT	0.90(0.46, 0.13)	0.39(0.68, 0.18)	0.92(0.33, 0.09)	0.76(0.49, 0.13)
Spindle density	.002	GCT	0.94(0.47, 0.12)	0.13(0.67, 0.18)	0.95(0.34, 0.08)	0.88(0.45, 0.13)
Spindle length	.002	GCT	0.88(0.47, 0.12)	0.05(0.64, 0.19)	0.87(0.35, 0.09)	0.86(0.47, 0.13)
Spindle amplitude	.003	GCT	0.90(0.44, 0.11)	0.26(0.64, 0.18)	0.91(0.33, 0.08)	0.84(0.46, 0.13)
Spindle frequency	<.001	GWT	0.96(0.48, 0.12)	0.69(0.70, 0.18)	0.93(0.31, 0.09)	0.96(0.48, 0.12)
EEG channel: P3A2						
Number of spindles	.021	GWT	0.85(0.45, 0.12)	0.52(0.62, 0.17)	0.88(0.32, 0.09)	0.89(0.47, 0.12)
Spindle density	.009	GWT	0.85(0.44, 0.12)	0.43(0.62, 0.19)	0.94(0.36, 0.08)	0.92(0.50, 0.12)
Spindle length	.007	GWT	0.69(0.49, 0.12)	-0.05(0.59, 0.20)	0.68(0.32, 0.09)	0.62(0.46, 0.13)
Spindle amplitude	.021	GCT	0.85(0.47, 0.13)	0.34(0.65, 0.19)	0.86(0.33, 0.08)	0.68(0.45, 0.14)
Spindle frequency	<.001	GWT	0.91(0.45, 0.12)	0.55(0.66, 0.18)	0.90(0.32, 0.09)	0.93(0.49, 0.13)

Results of genetic variance analysis, type of estimate applied (GCT: combined among- and within-twin pair component estimate, GWT: within-pair estimate) and intraclass correlation coefficients (ICCs). ICC MZ: ICCs of monozygotic (MZ) twins, ICC DZ: ICCs of dizygotic (DZ) twins, ICC MZ cn: ICCs of consecutive nights for each subject in MZ group, ICC DZ cn: ICCs of consecutive nights for each subject in DZ group. ICC results include: original sample ICC (upper percentile of bootstrapped data, median of bootstrapped data).

MZ_{match}: a subgroup of n = 14 MZ twin pairs who were closely matched for age, gender and cohabitation to the group of DZ twins (mean ± SD: MZ_{match}: 22.2±2.8yr, 18–27yr, 7m:7f, 10 pairs lived together; DZ: 22.1±2.7yr, 18–26yr, 7m:7f, 10 pairs lived together).

Supplementary Table S20: Individually adjusted frequency ranges. Genetic variance analysis, type of estimate applied (GCT: combined among- and within-twin pair component estimate, GWT: within-pair estimate) and intraclass correlation coefficients (ICCs) for fast spindle parameters in stage 2 sleep.

Variable	*P*	GWT vs GCT	ICC MZ_{match}	ICC DZ	ICC MZ_{match} cn	ICC DZ cn
EEG channel: Fp1A2						
Number of spindles	.001	GWT	0.90(0.80, 0.16)	0.33(0.75, 0.16)	0.90(0.55, 0.11)	0.91(0.53, 0.14)
Spindle density	<.001	GWT	0.91(0.77, 0.18)	0.38(0.68, 0.18)	0.91(0.55, 0.12)	0.94(0.56, 0.13)
Spindle length	.007	GWT	0.78(0.64, 0.19)	0.40(0.62, 0.18)	0.85(0.46, 0.13)	0.89(0.49, 0.13)
Spindle amplitude	.006	GCT	0.81(0.64, 0.18)	0.21(0.61, 0.18)	0.95(0.50, 0.13)	0.86(0.47, 0.14)
Spindle frequency	<.001	GWT	0.96(0.62, 0.19)	0.67(0.66, 0.18)	0.91(0.49, 0.13)	0.96(0.48, 0.12)
EEG channel: F3A2						
Number of spindles	.050	GWT	0.66(0.65, 0.18)	0.49(0.67, 0.19)	0.64(0.46, 0.13)	0.92(0.48, 0.13)
Spindle density	.045	GWT	0.67(0.68, 0.17)	0.54(0.66, 0.18)	0.62(0.50, 0.14)	0.94(0.46, 0.12)
Spindle length	.036	GWT	0.72(0.65, 0.18)	0.40(0.66, 0.19)	0.73(0.46, 0.12)	0.91(0.49, 0.14)
Spindle amplitude	<.001	GCT	0.90(0.61, 0.17)	0.10(0.64, 0.18)	0.87(0.48, 0.13)	0.74(0.47, 0.13)
Spindle frequency	<.001	GWT	0.97(0.65, 0.19)	0.67(0.66, 0.20)	0.95(0.45, 0.12)	0.96(0.50, 0.14)
EEG channel: C3A2						
Number of spindles	.241	GWT	0.64(0.66, 0.18)	0.61(0.62, 0.19)	0.78(0.45, 0.12)	0.82(0.47, 0.13)
Spindle density	.138	GWT	0.73(0.64, 0.18)	0.64(0.68, 0.19)	0.81(0.45, 0.13)	0.84(0.44, 0.12)
Spindle length	.004	GWT	0.85(0.64, 0.18)	0.55(0.62, 0.19)	0.91(0.46, 0.12)	0.93(0.47, 0.13)
Spindle amplitude	.014	GCT	0.89(0.65, 0.19)	0.33(0.61, 0.17)	0.94(0.51, 0.13)	0.83(0.46, 0.13)
Spindle frequency	<.001	GWT	0.96(0.62, 0.18)	0.66(0.64, 0.18)	0.95(0.47, 0.12)	0.97(0.48, 0.13)
EEG channel: P3A2						
Number of spindles	.136	GWT	0.81(0.64, 0.18)	0.70(0.62, 0.18)	0.88(0.49, 0.13)	0.86(0.45, 0.12)
Spindle density	.156	GWT	0.79(0.71, 0.19)	0.67(0.66, 0.18)	0.91(0.45, 0.14)	0.88(0.48, 0.13)
Spindle length	.002	GWT	0.85(0.69, 0.18)	0.48(0.62, 0.19)	0.92(0.47, 0.13)	0.92(0.47, 0.13)
Spindle amplitude	.162	GWT	0.78(0.63, 0.19)	0.57(0.61, 0.18)	0.91(0.48, 0.13)	0.64(0.47, 0.13)
Spindle frequency	<.001	GWT	0.96(0.61, 0.20)	0.70(0.66, 0.19)	0.95(0.46, 0.12)	0.98(0.47, 0.13)

Results of genetic variance analysis, type of estimate applied (GCT: combined among- and within-twin pair component estimate, GWT: within-pair estimate) and intraclass correlation coefficients (ICCs). ICC MZ: ICCs of monozygotic (MZ) twins, ICC DZ: ICCs of dizygotic (DZ) twins, ICC MZ cn: ICCs of consecutive nights for each subject in MZ group, ICC DZ cn: ICCs of consecutive nights for each subject in DZ group. ICC results include: original sample ICC (upper percentile of bootstrapped data, median of bootstrapped data).

Supplementary Table S21: Individually adjusted frequency ranges. Genetic variance analysis, type of estimate applied (GCT: combined among- and within-twin pair component estimate, GWT: within-pair estimate) and intraclass correlation coefficients (ICCs) for fast spindle parameters in slow wave sleep.

Variable	*P*	GWT vs GCT	ICC MZ_{match}	ICC DZ	ICC MZ_{match} cn	ICC DZ cn
EEG channel: Fp1A2						
Number of spindles	.818	GCT	0.31(0.77, 0.18)	0.33(0.81, 0.14)	0.89(0.60, 0.11)	0.94(0.71, 0.11)
Spindle density	.825	GCT	0.40(0.75, 0.16)	0.40(0.89, 0.15)	0.85(0.57, 0.12)	0.88(0.76, 0.11)
Spindle length	.059	GWT	0.28(0.83, 0.17)	0.03(0.73, 0.21)	0.07(0.53, 0.15)	0.12(0.56, 0.14)
Spindle amplitude	.031	GWT	0.84(0.87, 0.25)	0.21(0.76, 0.23)	0.83(0.56, 0.14)	0.78(0.52, 0.14)
Spindle frequency	.002	GWT	0.94(0.87, 0.19)	0.71(0.68, 0.21)	0.83(0.56, 0.15)	0.94(0.51, 0.14)
EEG channel: F3A2						
Number of spindles	.095	GWT	0.45(0.70, 0.19)	0.45(0.75, 0.14)	0.78(0.52, 0.13)	0.93(0.59, 0.11)
Spindle density	.070	GWT	0.55(0.69, 0.18)	0.51(0.80, 0.15)	0.70(0.51, 0.12)	0.89(0.58, 0.12)
Spindle length	.007	GWT	0.82(0.65, 0.19)	0.42(0.64, 0.18)	0.55(0.45, 0.13)	0.85(0.47, 0.13)
Spindle amplitude	<.001	GCT	0.84(0.73, 0.19)	-0.30(0.60, 0.18)	0.89(0.51, 0.12)	0.60(0.50, 0.13)
Spindle frequency	.082	GWT	0.81(0.67, 0.19)	0.73(0.64, 0.19)	0.85(0.44, 0.13)	0.90(0.49, 0.14)
EEG channel: C3A2						
Number of spindles	.128	GWT	0.72(0.69, 0.18)	0.39(0.70, 0.19)	0.88(0.50, 0.12)	0.76(0.51, 0.13)
Spindle density	.048	GWT	0.81(0.66, 0.19)	0.41(0.64, 0.20)	0.89(0.44, 0.12)	0.75(0.47, 0.13)
Spindle length	.047	GWT	0.86(0.64, 0.18)	0.62(0.61, 0.19)	0.83(0.49, 0.13)	0.83(0.47, 0.13)
Spindle amplitude	<.001	GCT	0.89(0.67, 0.19)	-0.06(0.63, 0.18)	0.87(0.47, 0.13)	0.67(0.47, 0.13)
Spindle frequency	.045	GWT	0.83(0.65, 0.19)	0.72(0.63, 0.19)	0.89(0.47, 0.13)	0.94(0.46, 0.13)
EEG channel: P3A2						
Number of spindles	.259	GWT	0.52(0.64, 0.18)	0.15(0.68, 0.19)	0.93(0.52, 0.14)	0.83(0.51, 0.13)
Spindle density	.092	GWT	0.68(0.64, 0.19)	0.21(0.61, 0.18)	0.92(0.48, 0.14)	0.80(0.47, 0.13)
Spindle length	.068	GWT	0.79(0.68, 0.18)	0.47(0.66, 0.18)	0.84(0.49, 0.14)	0.94(0.46, 0.13)
Spindle amplitude	.015	GCT	0.80(0.70, 0.19)	0.29(0.59, 0.18)	0.91(0.48, 0.13)	0.56(0.49, 0.14)
Spindle frequency	.002	GWT	0.93(0.63, 0.18)	0.77(0.68, 0.20)	0.90(0.45, 0.13)	0.96(0.47, 0.13)

Results of genetic variance analysis, type of estimate applied (GCT: combined among- and within-twin pair component estimate, GWT: within-pair estimate) and intraclass correlation coefficients (ICCs). ICC MZ: ICCs of monozygotic (MZ) twins, ICC DZ: ICCs of dizygotic (DZ) twins, ICC MZ cn: ICCs of consecutive nights for each subject in MZ group, ICC DZ cn: ICCs of consecutive nights for each subject in DZ group. ICC results include: original sample ICC (upper percentile of bootstrapped data, median of bootstrapped data).

Supplementary Table S22: Individually adjusted frequency ranges. Genetic variance analysis, type of estimate applied (GCT: combined among- and within-twin pair component estimate, GWT: within-pair estimate) and intraclass correlation coefficients (ICCs) for slow spindle parameters in stage 2 sleep.

Variable	*P*	GWT vs GCT	ICC MZ_{match}	ICC DZ	ICC MZ_{match} cn	ICC DZ cn
EEG channel: Fp1A2						
Number of spindles	.006	GWT	0.86(0.64, 0.19)	0.36(0.66, 0.19)	0.94(0.48, 0.13)	0.85(0.46, 0.13)
Spindle density	.007	GCT	0.93(0.64, 0.19)	0.24(0.65, 0.19)	0.95(0.49, 0.14)	0.91(0.47, 0.13)
Spindle length	<.001	GCT	0.95(0.72, 0.18)	0.40(0.62, 0.18)	0.98(0.50, 0.13)	0.92(0.46, 0.13)
Spindle amplitude	.014	GCT	0.84(0.68, 0.19)	0.23(0.66, 0.18)	0.94(0.49, 0.14)	0.92(0.47, 0.12)
Spindle frequency	<.001	GWT	0.95(0.64, 0.18)	0.38(0.63, 0.18)	0.87(0.48, 0.13)	0.97(0.51, 0.13)
EEG channel: F3A2						
Number of spindles	.004	GWT	0.88(0.66, 0.19)	0.43(0.67, 0.18)	0.90(0.49, 0.13)	0.88(0.48, 0.12)
Spindle density	.007	GCT	0.94(0.64, 0.19)	0.24(0.64, 0.19)	0.91(0.48, 0.13)	0.91(0.47, 0.13)
Spindle length	.001	GCT	0.97(0.73, 0.18)	0.44(0.63, 0.19)	0.97(0.51, 0.13)	0.92(0.46, 0.13)
Spindle amplitude	<.001	GWT	0.90(0.67, 0.19)	0.19(0.61, 0.18)	0.88(0.48, 0.13)	0.88(0.49, 0.14)
Spindle frequency	<.001	GWT	0.93(0.61, 0.20)	0.43(0.66, 0.18)	0.86(0.45, 0.13)	0.96(0.46, 0.13)
EEG channel: C3A2						
Number of spindles	.003	GWT	0.90(0.64, 0.18)	0.39(0.62, 0.18)	0.88(0.48, 0.13)	0.86(0.52, 0.13)
Spindle density	.003	GCT	0.94(0.68, 0.18)	0.20(0.68, 0.19)	0.90(0.49, 0.13)	0.92(0.49, 0.13)
Spindle length	.001	GCT	0.97(0.67, 0.17)	0.33(0.69, 0.18)	0.93(0.58, 0.12)	0.83(0.46, 0.13)
Spindle amplitude	.001	GWT	0.90(0.65, 0.19)	0.41(0.66, 0.16)	0.96(0.44, 0.12)	0.92(0.51, 0.12)
Spindle frequency	<.001	GWT	0.90(0.62, 0.19)	0.51(0.61, 0.17)	0.81(0.49, 0.13)	0.95(0.45, 0.13)
EEG channel: P3A2						
Number of spindles	.009	GCT	0.93(0.66, 0.19)	0.22(0.66, 0.19)	0.87(0.53, 0.13)	0.88(0.50, 0.14)
Spindle density	<.001	GCT	0.97(0.64, 0.18)	0.04(0.67, 0.19)	0.88(0.51, 0.13)	0.91(0.48, 0.13)
Spindle length	<.001	GCT	0.93(0.73, 0.17)	-0.19(0.66, 0.18)	0.84(0.58, 0.12)	0.81(0.46, 0.14)
Spindle amplitude	.056	GWT	0.75(0.65, 0.19)	0.50(0.64, 0.18)	0.89(0.47, 0.13)	0.79(0.45, 0.13)
Spindle frequency	<.001	GWT	0.90(0.64, 0.19)	0.41(0.62, 0.18)	0.80(0.47, 0.14)	0.95(0.50, 0.13)

Results of genetic variance analysis, type of estimate applied (GCT: combined among- and within-twin pair component estimate, GWT: within-pair estimate) and intraclass correlation coefficients (ICCs). ICC MZ: ICCs of monozygotic (MZ) twins, ICC DZ: ICCs of dizygotic (DZ) twins, ICC MZ cn: ICCs of consecutive nights for each subject in MZ group, ICC DZ cn: ICCs of consecutive nights for each subject in DZ group. ICC results include: original sample ICC (upper percentile of bootstrapped data, median of bootstrapped data).

Supplementary Table S23: Individually adjusted frequency ranges. Genetic variance analysis, type of estimate applied (GCT: combined among- and within-twin pair component estimate, GWT: within-pair estimate) and intraclass correlation coefficients (ICCs) for slow spindle parameters in slow wave sleep.

Variable	*P*	GWT vs GCT	ICC MZ_{match}	ICC DZ	ICC MZ_{match} cn	ICC DZ cn
EEG channel: Fp1A2						
Number of spindles	.005	GCT	0.96(0.64, 0.18)	0.23(0.67, 0.17)	0.95(0.50, 0.13)	0.85(0.48, 0.12)
Spindle density	.001	GCT	0.97(0.63, 0.19)	0.18(0.66, 0.18)	0.97(0.47, 0.13)	0.95(0.45, 0.13)
Spindle length	<.001	GCT	0.99(0.76, 0.17)	0.33(0.76, 0.16)	0.96(0.54, 0.13)	0.94(0.53, 0.13)
Spindle amplitude	<.001	GCT	0.90(0.66, 0.19)	0.18(0.64, 0.19)	0.94(0.48, 0.13)	0.88(0.47, 0.13)
Spindle frequency	.022	GWT	0.94(0.62, 0.19)	0.81(0.63, 0.19)	0.96(0.47, 0.13)	0.97(0.48, 0.13)
EEG channel: F3A2						
Number of spindles	.003	GCT	0.95(0.66, 0.18)	0.14(0.66, 0.17)	0.94(0.48, 0.12)	0.76(0.50, 0.13)
Spindle density	<.001	GCT	0.98(0.66, 0.18)	0.05(0.62, 0.18)	0.97(0.50, 0.14)	0.91(0.47, 0.13)
Spindle length	<.001	GCT	0.99(0.81, 0.17)	0.45(0.71, 0.19)	0.92(0.60, 0.11)	0.89(0.49, 0.12)
Spindle amplitude	<.001	GCT	0.93(0.63, 0.18)	0.16(0.63, 0.18)	0.92(0.49, 0.13)	0.78(0.47, 0.13)
Spindle frequency	.019	GWT	0.94(0.64, 0.18)	0.81(0.62, 0.19)	0.95(0.47, 0.13)	0.97(0.47, 0.12)
EEG channel: C3A2						
Number of spindles	<.001	GWT	0.96(0.69, 0.19)	0.20(0.65, 0.18)	0.93(0.51, 0.13)	0.73(0.51, 0.13)
Spindle density	<.001	GWT	0.97(0.66, 0.19)	0.41(0.64, 0.18)	0.93(0.49, 0.13)	0.89(0.51, 0.12)
Spindle length	<.001	GCT	0.98(0.85, 0.15)	0.64(0.68, 0.18)	0.97(0.62, 0.11)	0.90(0.55, 0.13)
Spindle amplitude	<.001	GCT	0.95(0.68, 0.19)	0.31(0.59, 0.18)	0.89(0.50, 0.13)	0.81(0.45, 0.13)
Spindle frequency	.028	GWT	0.92(0.68, 0.19)	0.78(0.63, 0.18)	0.94(0.45, 0.13)	0.97(0.45, 0.14)
EEG channel: P3A2						
Number of spindles	.008	GCT	0.96(0.86, 0.15)	0.42(0.72, 0.18)	0.94(0.62, 0.12)	0.90(0.49, 0.14)
Spindle density	<.001	GWT	0.98(0.83, 0.15)	0.64(0.70, 0.19)	0.96(0.62, 0.12)	0.95(0.51, 0.13)
Spindle length	<.001	GCT	0.96(0.86, 0.13)	0.46(0.65, 0.19)	0.92(0.64, 0.11)	0.90(0.49, 0.13)
Spindle amplitude	.019	GWT	0.85(0.60, 0.17)	0.41(0.64, 0.18)	0.87(0.47, 0.13)	0.73(0.48, 0.13)
Spindle frequency	.005	GWT	0.92(0.64, 0.19)	0.69(0.66, 0.18)	0.91(0.47, 0.13)	0.93(0.48, 0.12)

Results of genetic variance analysis, type of estimate applied (GCT: combined among- and within-twin pair component estimate, GWT: within-pair estimate) and intraclass correlation coefficients (ICCs). ICC MZ: ICCs of monozygotic (MZ) twins, ICC DZ: ICCs of dizygotic (DZ) twins, ICC MZ cn: ICCs of consecutive nights for each subject in MZ group, ICC DZ cn: ICCs of consecutive nights for each subject in DZ group. ICC results include: original sample ICC (upper percentile of bootstrapped data, median of bootstrapped data).

2.2. Supplementary Figures

Spindle distribution in all twin pairs used in the analysis.

MZ twins:

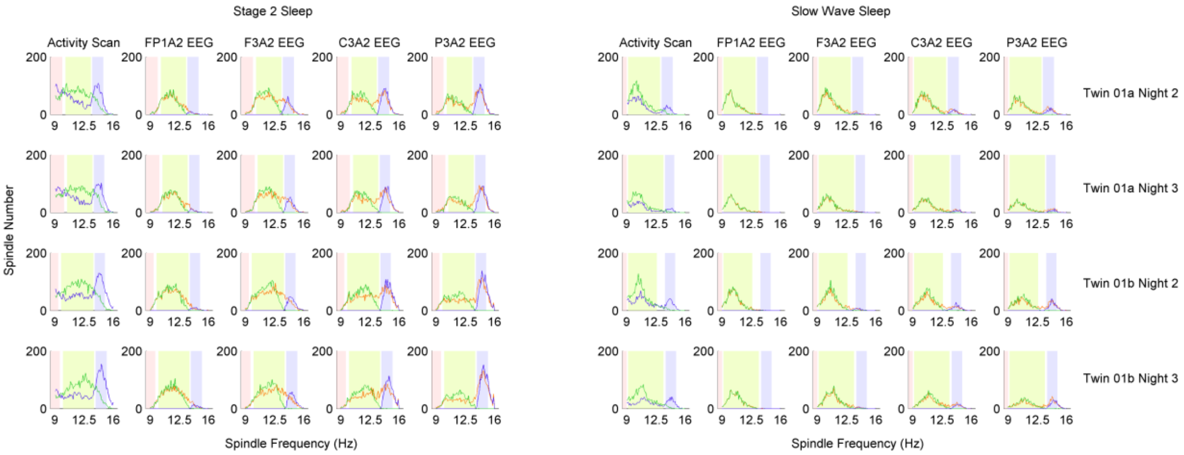

Supplementary Figure S1: Distribution of detected sleep spindles in 0.1 Hz frequency bins. Analysis was performed separately for stage 2 and slow wave sleep. Each row of plots represents one recording night. Column *Activity Scan* shows the result of pre-analysis performed to localize slow and fast spindle frequency ranges. During *activity scan* spindles were detected in two EEG derivations: parietal channel P3A2 (blue color) and frontal channel F3A2 (green color). Information from *activity scan* was used to set frequency range of fast spindles (light blue color), slow spindles (light green color) and range in which spindles should not be detected anymore (light red color). Localized frequency ranges were used to detect sleep spindles in four EEG derivations, which are presented in distinct columns: *FP1A2*, *F3A2*, *C3A2* and *P3A2*. Blue color depicts sleep spindles detected with wavelets in fast spindle frequency range, green color depicts sleep spindles detected with wavelets in slow spindle frequency range whereas orange color depicts sleep spindles detected with combined slow and fast spindle frequency ranges.

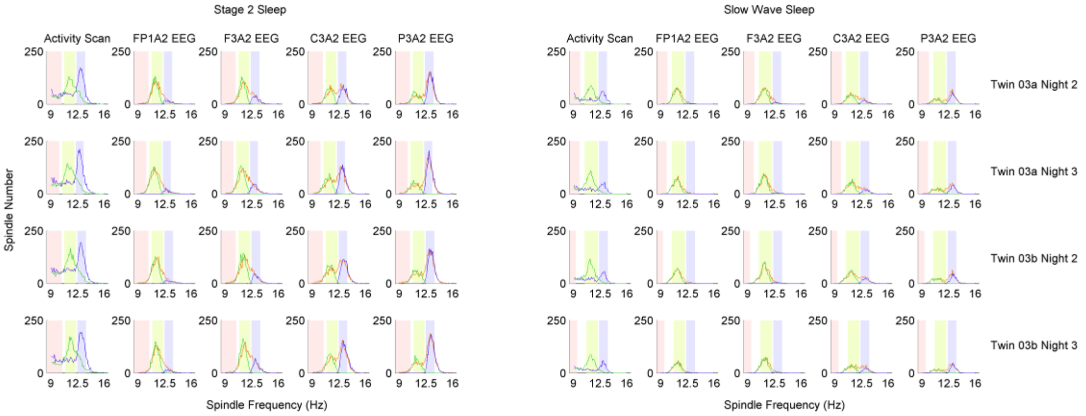

Supplementary Figure S2: Distribution of detected sleep spindles in 0.1 Hz frequency bins. Plots explanation as in Figure S1.

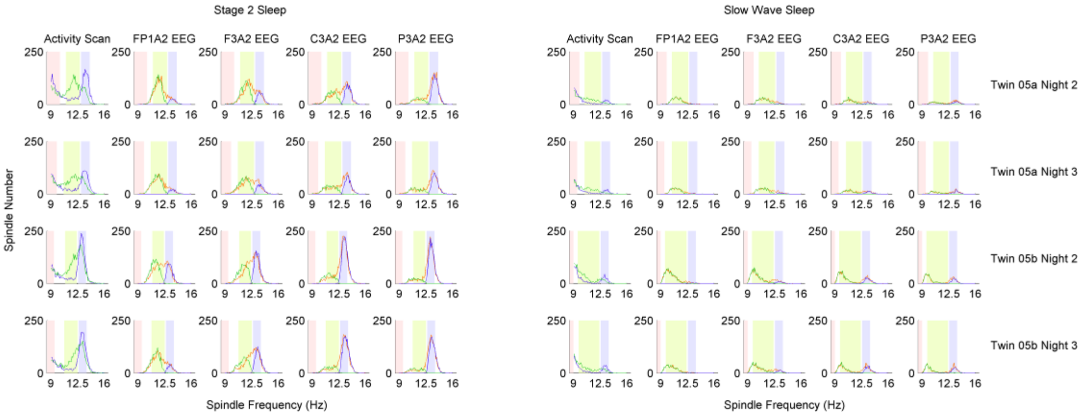

Supplementary Figure S3: Distribution of detected sleep spindles in 0.1 Hz frequency bins. Plots explanation as in Figure S1.

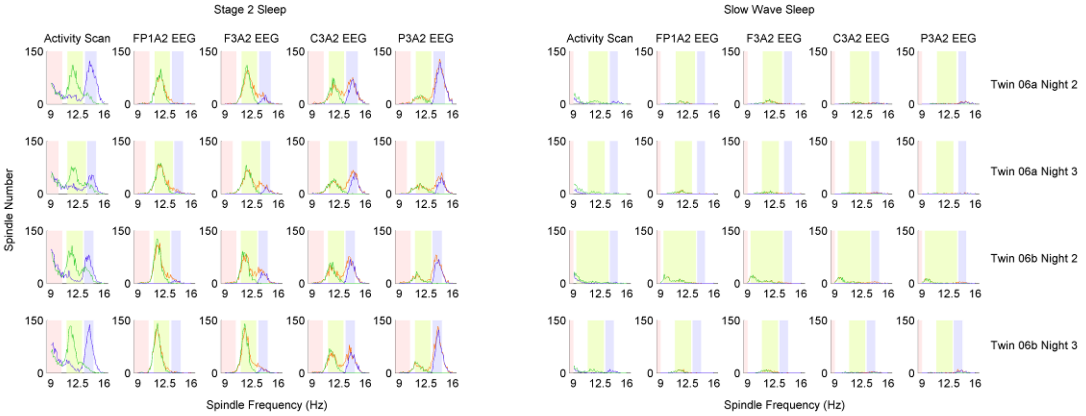

Supplementary Figure S4: Distribution of detected sleep spindles in 0.1 Hz frequency bins. Plots explanation as in Figure S1.

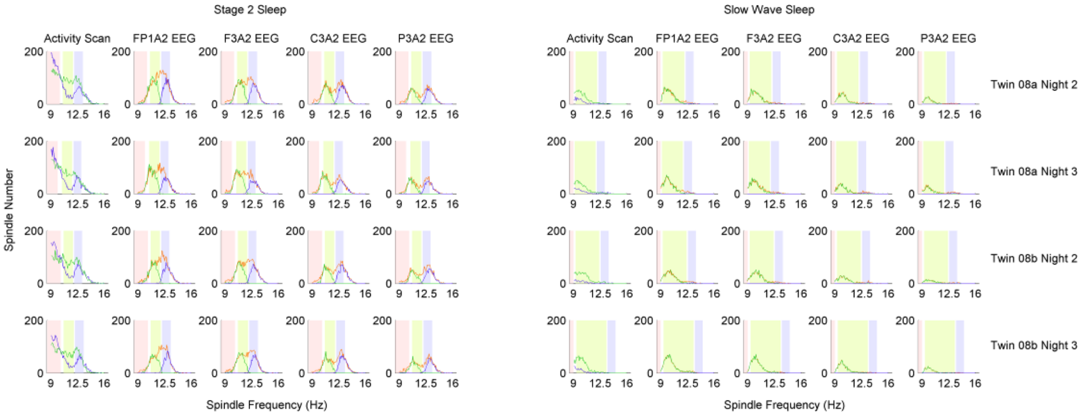

Supplementary Figure S5: Distribution of detected sleep spindles in 0.1 Hz frequency bins. Plots explanation as in Figure S1.

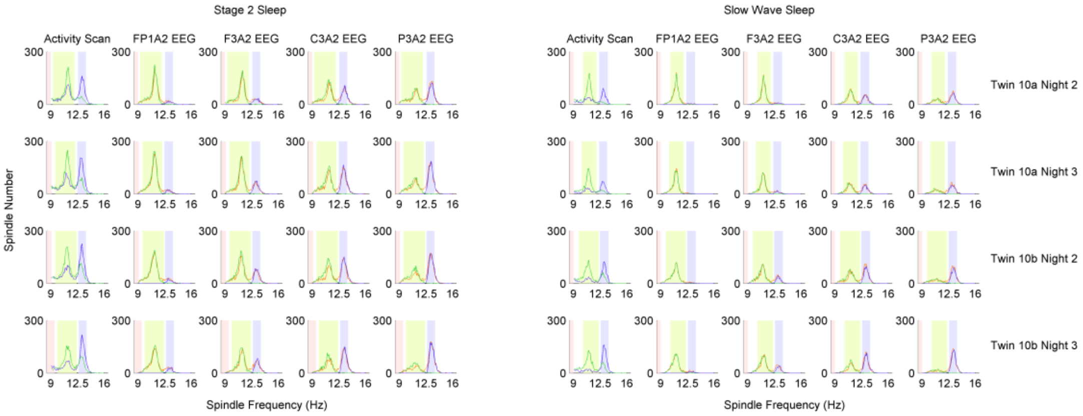

Supplementary Figure S6: Distribution of detected sleep spindles in 0.1 Hz frequency bins. Plots explanation as in Figure S1.

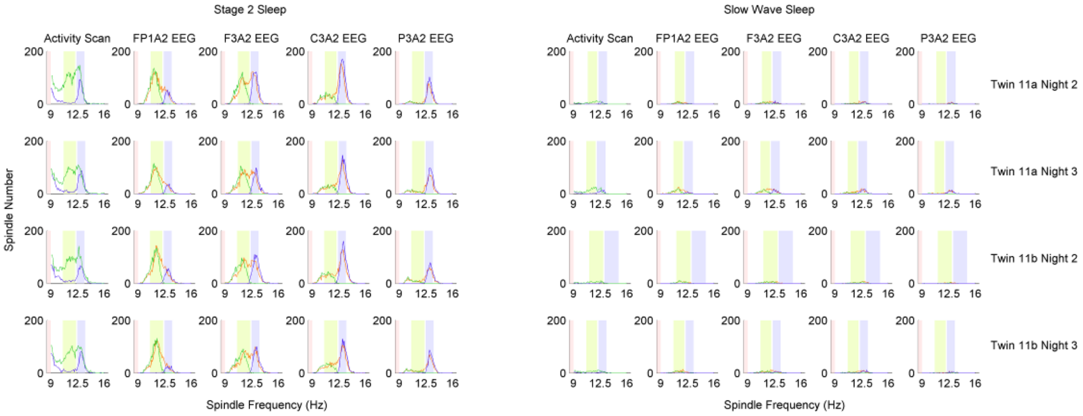

Supplementary Figure S7: Distribution of detected sleep spindles in 0.1 Hz frequency bins. Plots explanation as in Figure S1.

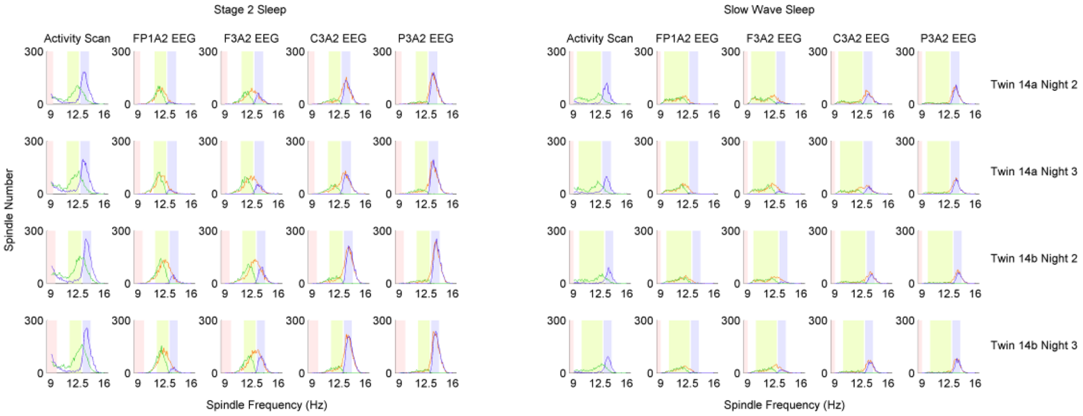

Supplementary Figure S8: Distribution of detected sleep spindles in 0.1 Hz frequency bins. Plots explanation as in Figure S1.

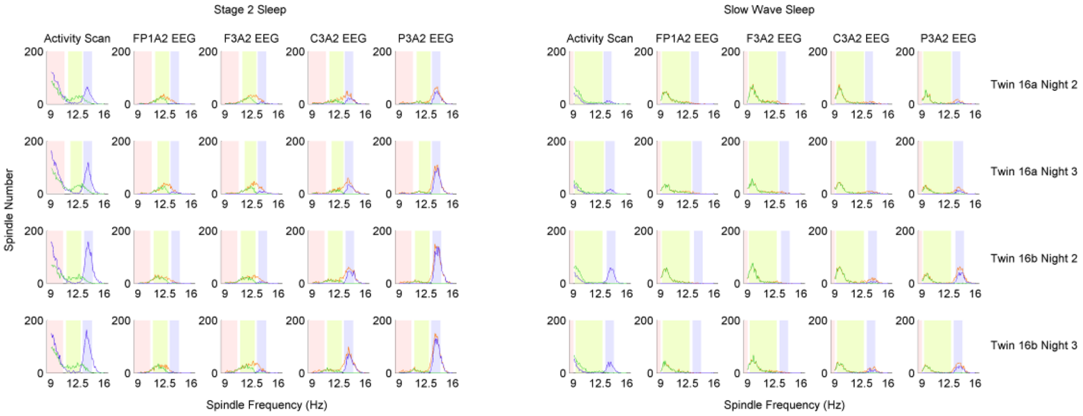

Supplementary Figure S9: Distribution of detected sleep spindles in 0.1 Hz frequency bins. Plots explanation as in Figure S1.

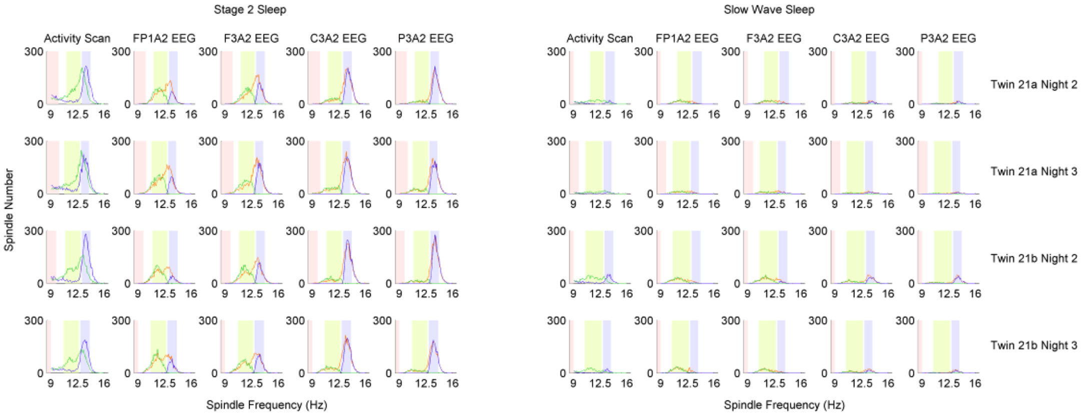

Supplementary Figure S10: Distribution of detected sleep spindles in 0.1 Hz frequency bins. Plots explanation as in Figure S1.

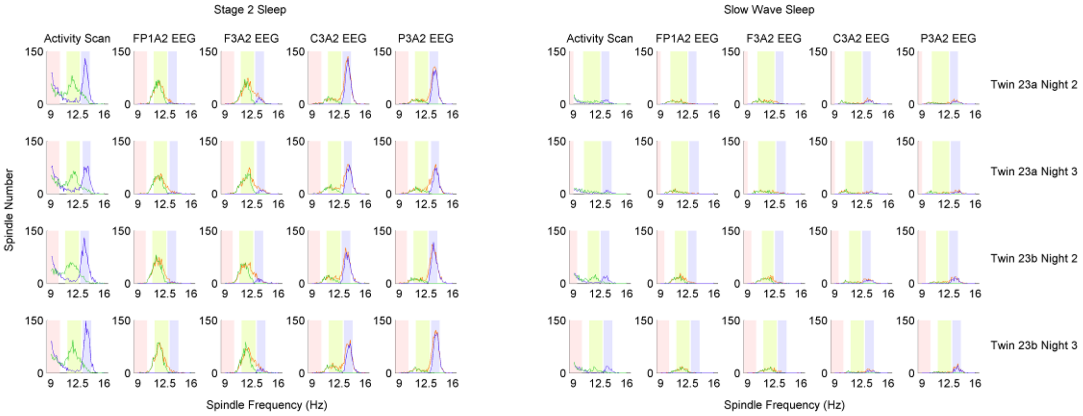

Supplementary Figure S11: Distribution of detected sleep spindles in 0.1 Hz frequency bins. Plots explanation as in Figure S1.

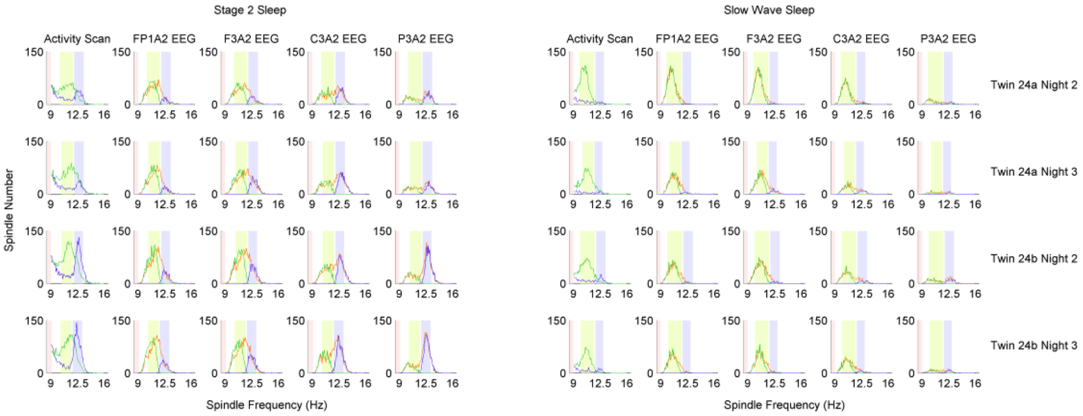

Supplementary Figure S12: Distribution of detected sleep spindles in 0.1 Hz frequency bins. Plots explanation as in Figure S1.

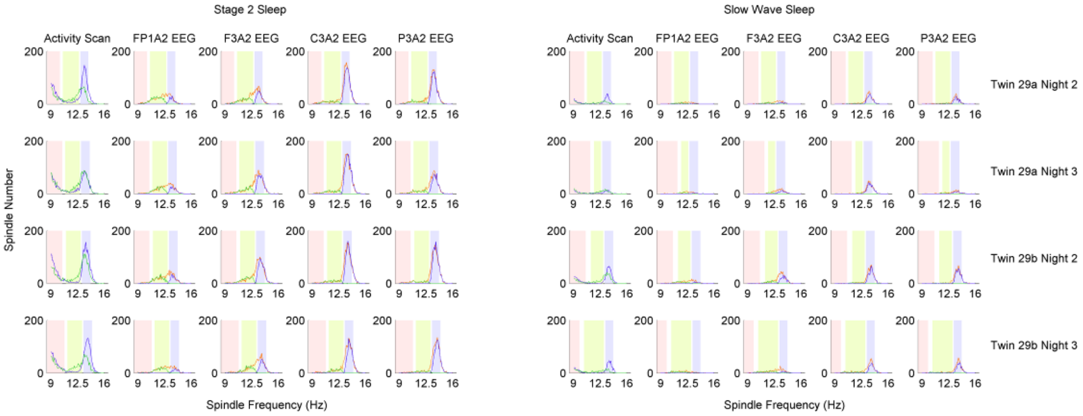

Supplementary Figure S13: Distribution of detected sleep spindles in 0.1 Hz frequency bins. Plots explanation as in Figure S1.

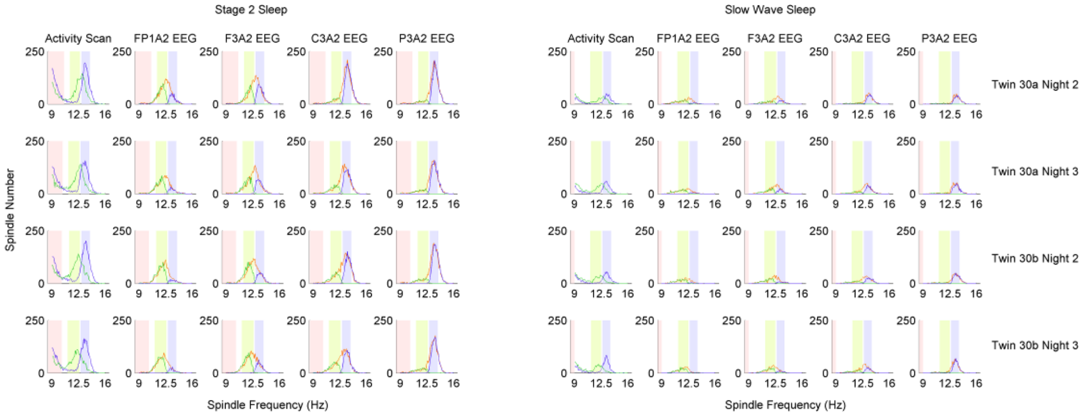

Supplementary Figure S14: Distribution of detected sleep spindles in 0.1 Hz frequency bins. Plots explanation as in Figure S1.

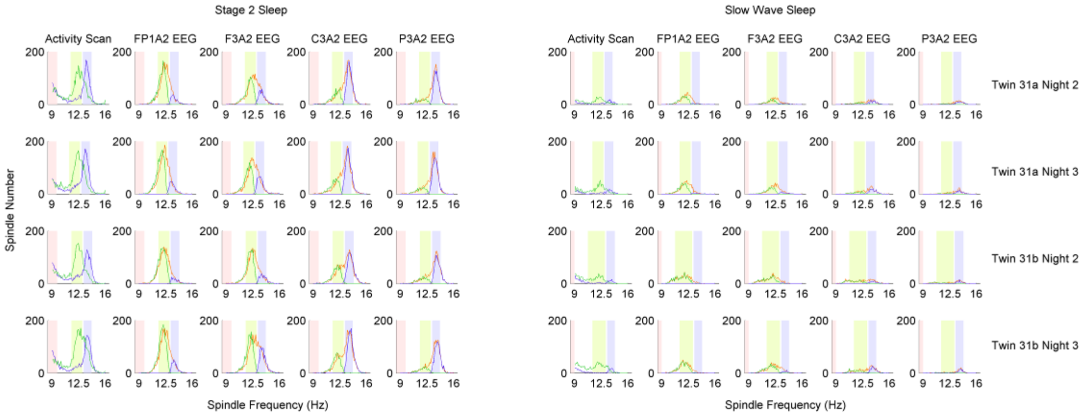

Supplementary Figure S15: Distribution of detected sleep spindles in 0.1 Hz frequency bins. Plots explanation as in Figure S1.

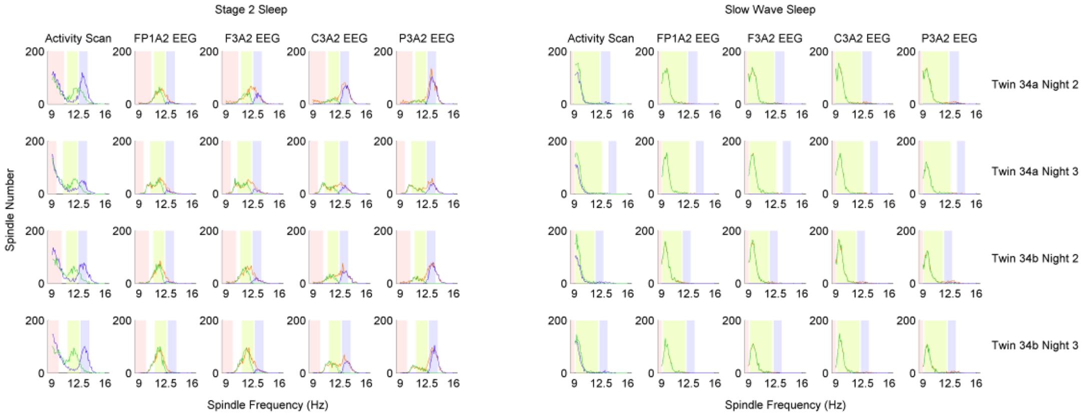

Supplementary Figure S16: Distribution of detected sleep spindles in 0.1 Hz frequency bins. Plots explanation as in Figure S1.

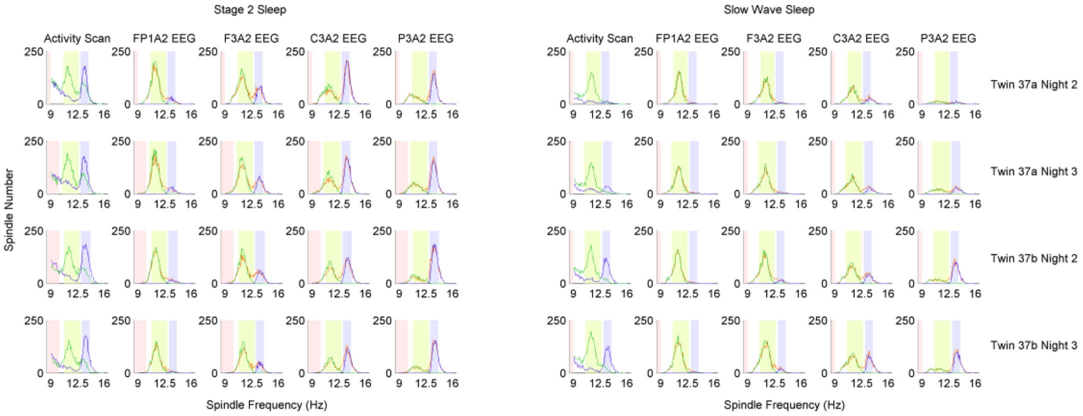

Supplementary Figure S17: Distribution of detected sleep spindles in 0.1 Hz frequency bins. Plots explanation as in Figure S1.

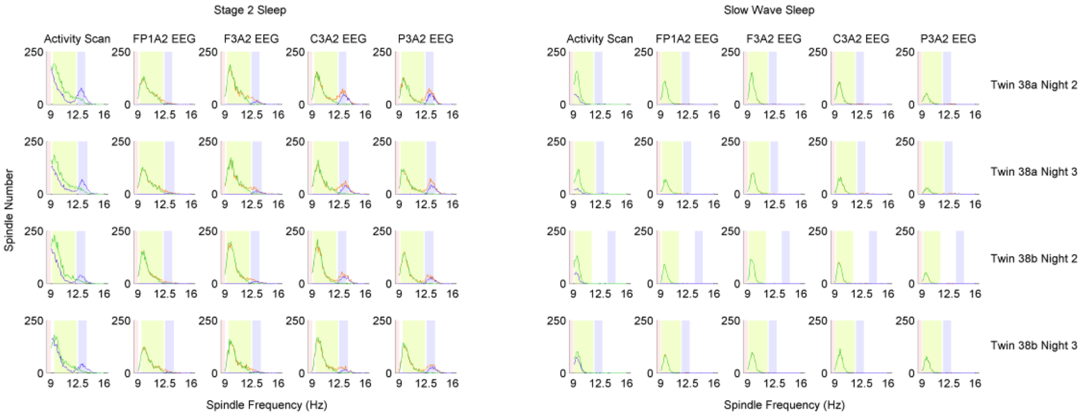

Supplementary Figure S18: Distribution of detected sleep spindles in 0.1 Hz frequency bins. Plots explanation as in Figure S1.

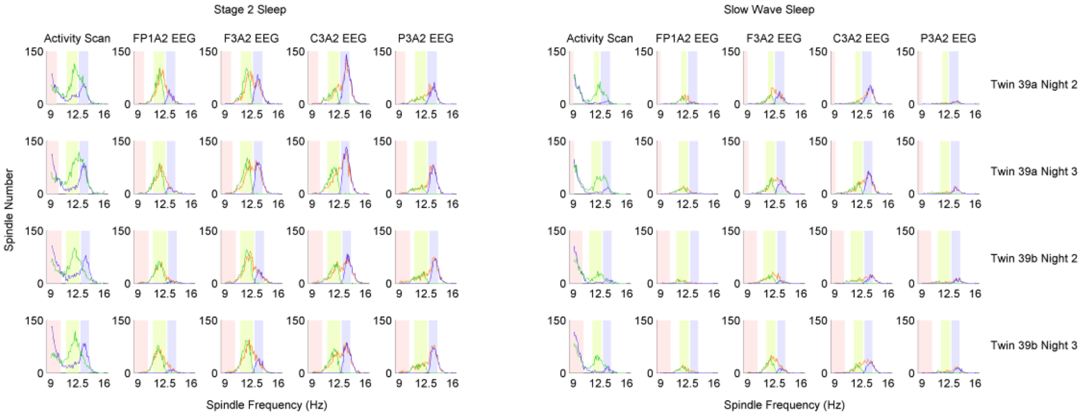

Supplementary Figure S19: Distribution of detected sleep spindles in 0.1 Hz frequency bins. Plots explanation as in Figure S1.

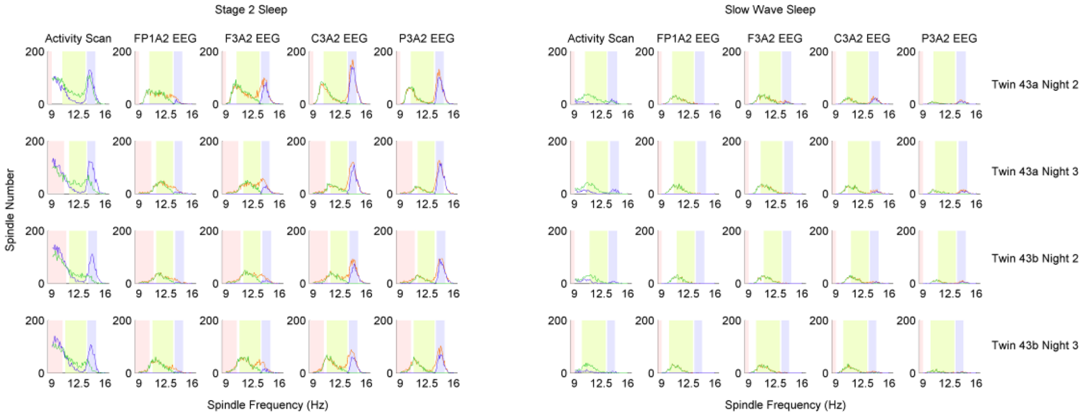

Supplementary Figure S20: Distribution of detected sleep spindles in 0.1 Hz frequency bins. Plots explanation as in Figure S1.

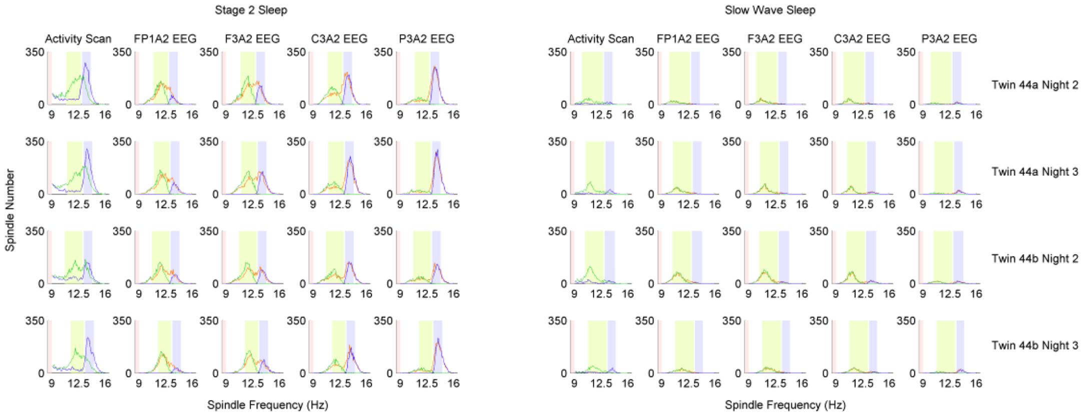

Supplementary Figure S21: Distribution of detected sleep spindles in 0.1 Hz frequency bins. Plots explanation as in Figure S1.

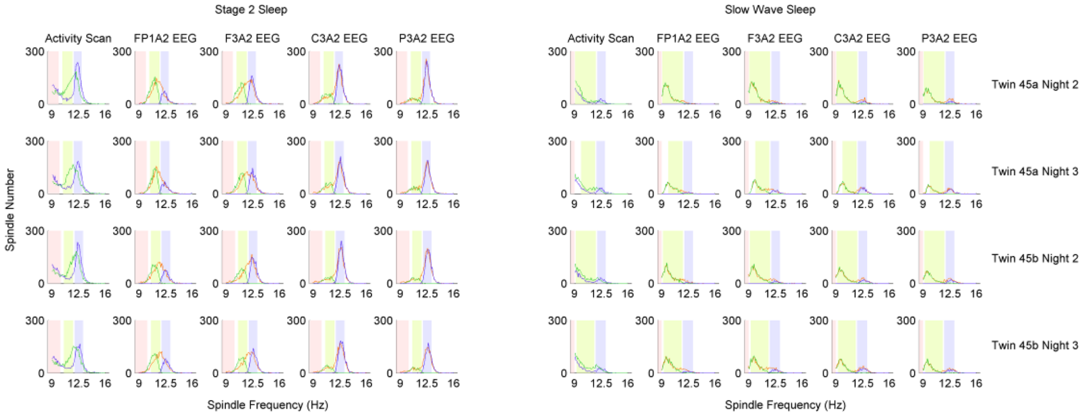

Supplementary Figure S22: Distribution of detected sleep spindles in 0.1 Hz frequency bins. Plots explanation as in Figure S1.

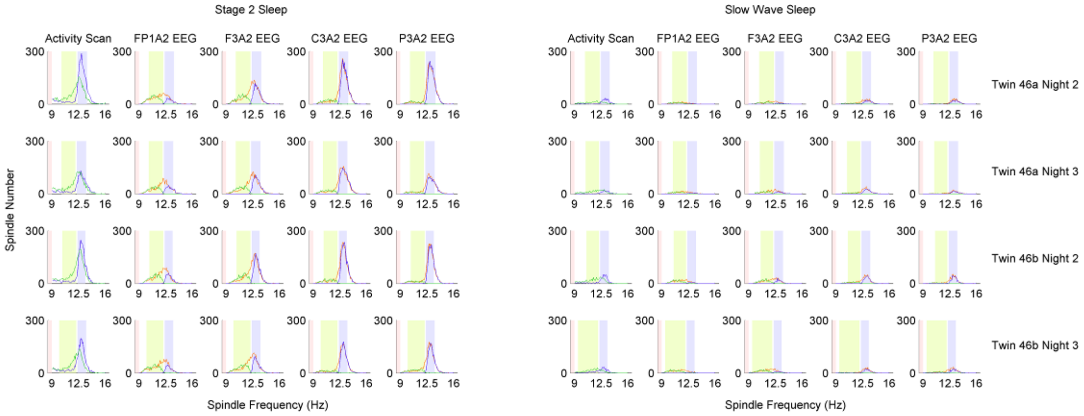

Supplementary Figure S23: Distribution of detected sleep spindles in 0.1 Hz frequency bins. Plots explanation as in Figure S1.

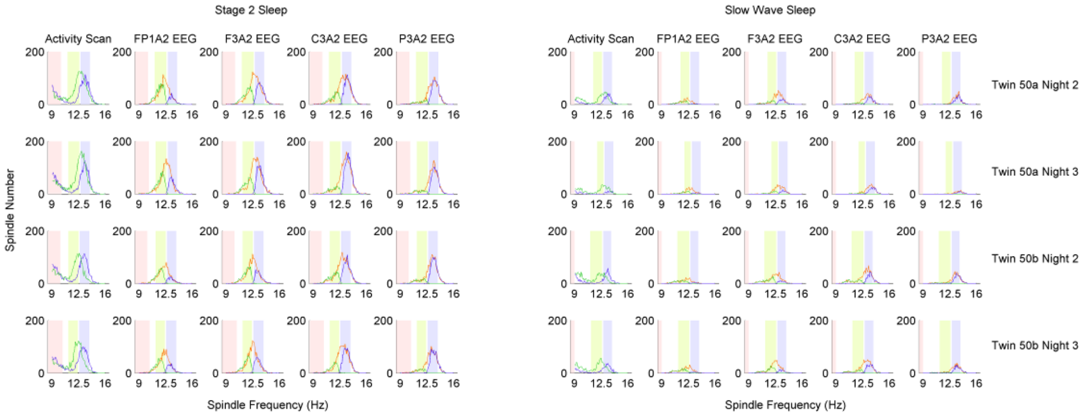

Supplementary Figure S24: Distribution of detected sleep spindles in 0.1 Hz frequency bins. Plots explanation as in Figure S1.

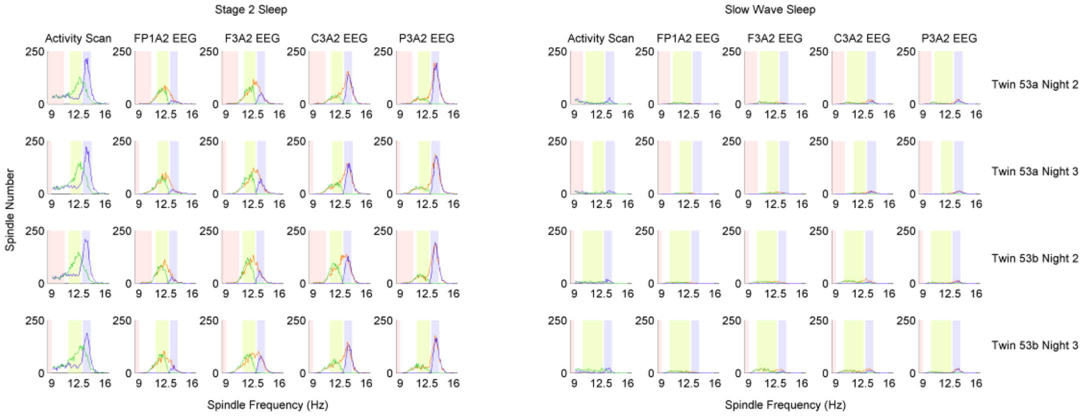

Supplementary Figure S25: Distribution of detected sleep spindles in 0.1 Hz frequency bins. Plots explanation as in Figure S1.

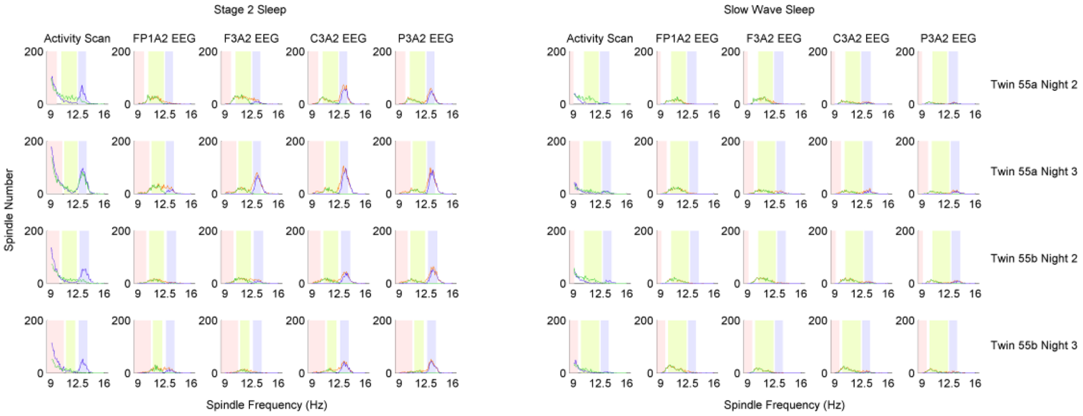

Supplementary Figure S26: Distribution of detected sleep spindles in 0.1 Hz frequency bins. Plots explanation as in Figure S1.

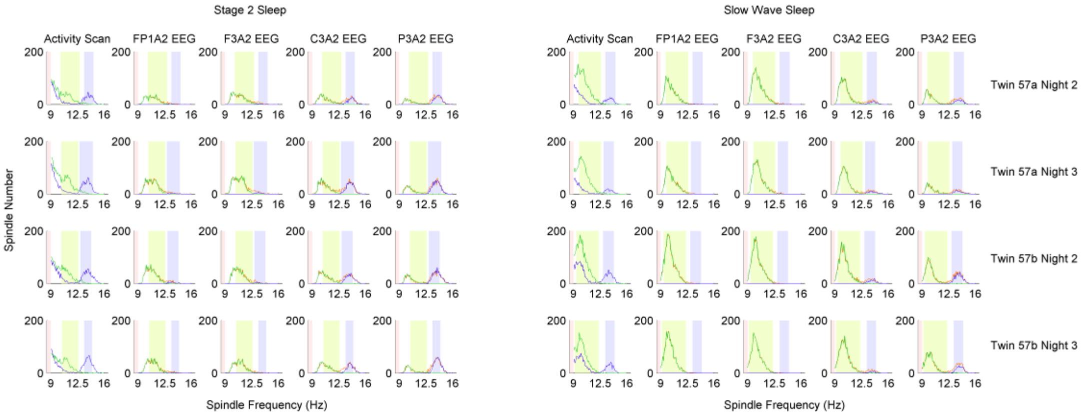

Supplementary Figure S27: Distribution of detected sleep spindles in 0.1 Hz frequency bins. Plots explanation as in Figure S1.

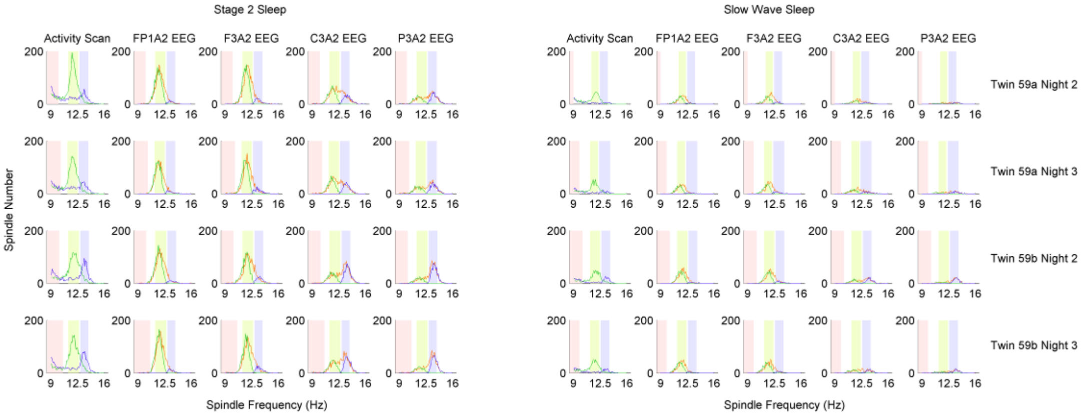

Supplementary Figure S28: Distribution of detected sleep spindles in 0.1 Hz frequency bins. Plots explanation as in Figure S1.

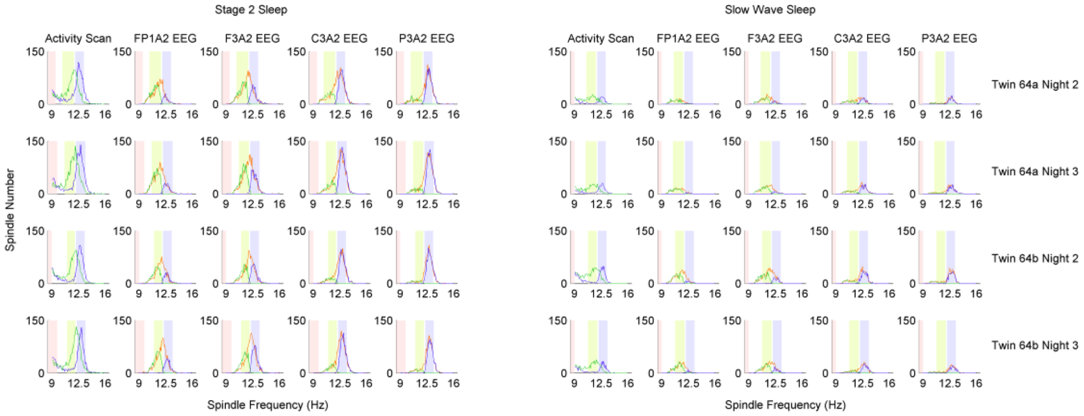

Supplementary Figure S29: Distribution of detected sleep spindles in 0.1 Hz frequency bins. Plots explanation as in Figure S1.

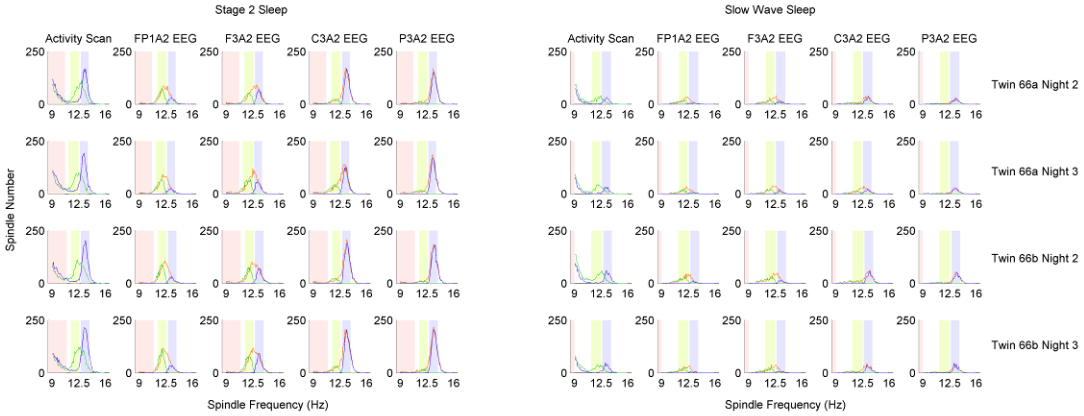

Supplementary Figure S30: Distribution of detected sleep spindles in 0.1 Hz frequency bins. Plots explanation as in Figure S1.

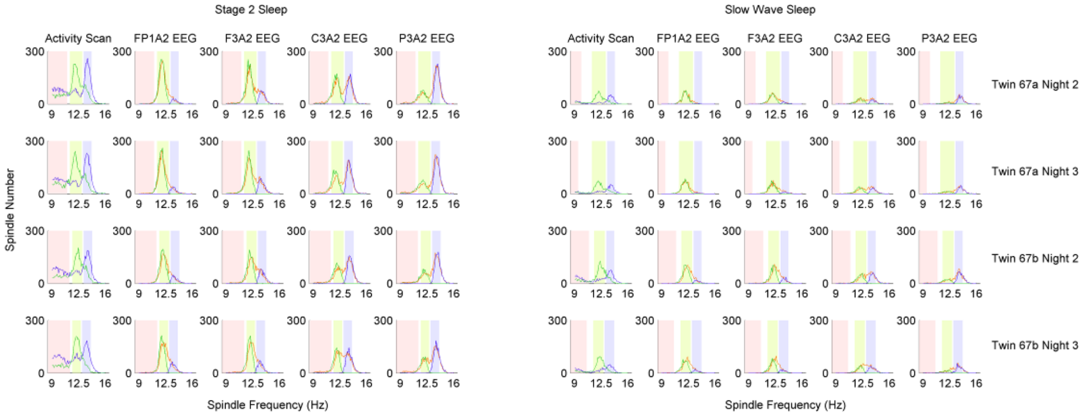

Supplementary Figure S31: Distribution of detected sleep spindles in 0.1 Hz frequency bins. Plots explanation as in Figure S1.

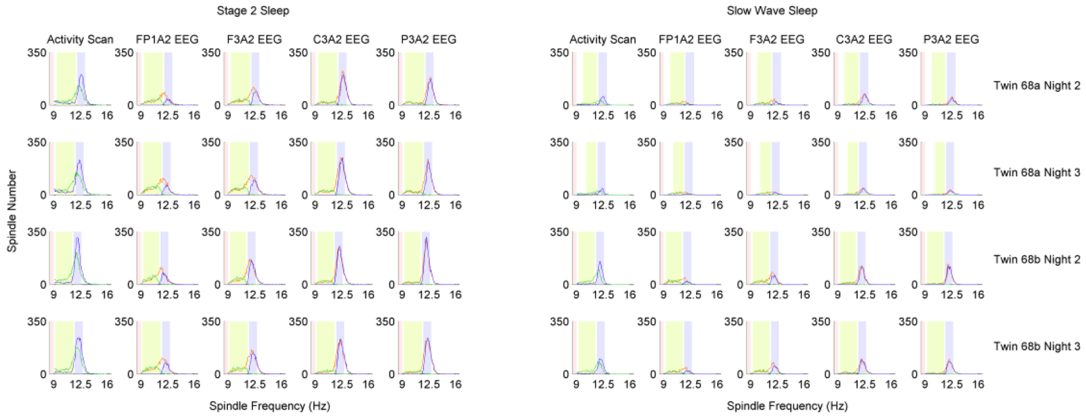

Supplementary Figure S32: Distribution of detected sleep spindles in 0.1 Hz frequency bins. Plots explanation as in Figure S1.

DZ twins**:**

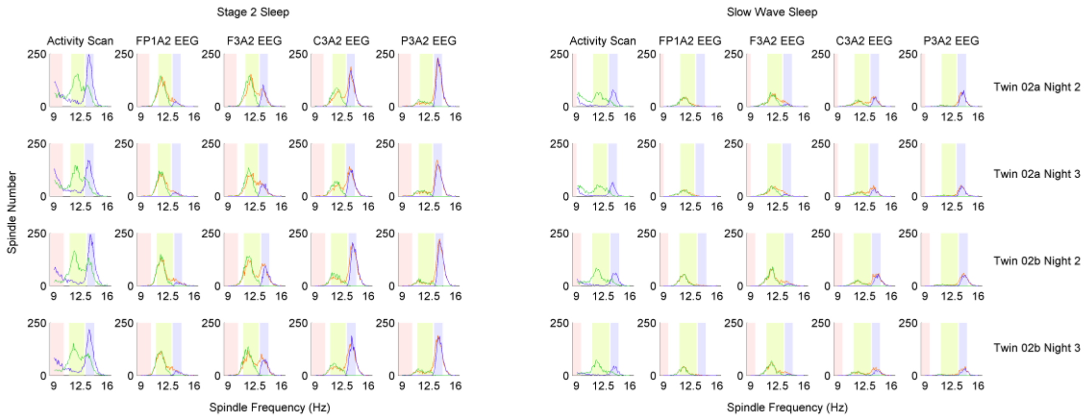

Supplementary Figure S33: Distribution of detected sleep spindles in 0.1 Hz frequency bins. Plots explanation as in Figure S1.

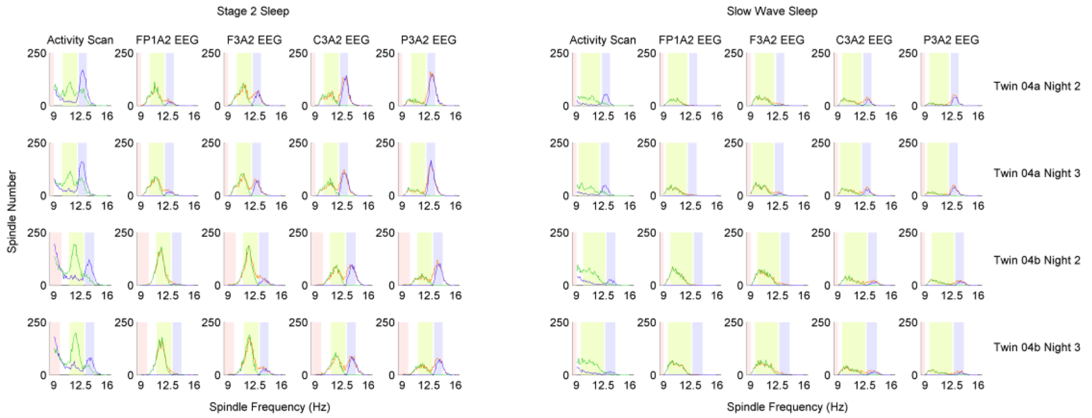

Supplementary Figure S34: Distribution of detected sleep spindles in 0.1 Hz frequency bins. Plots explanation as in Figure S1.

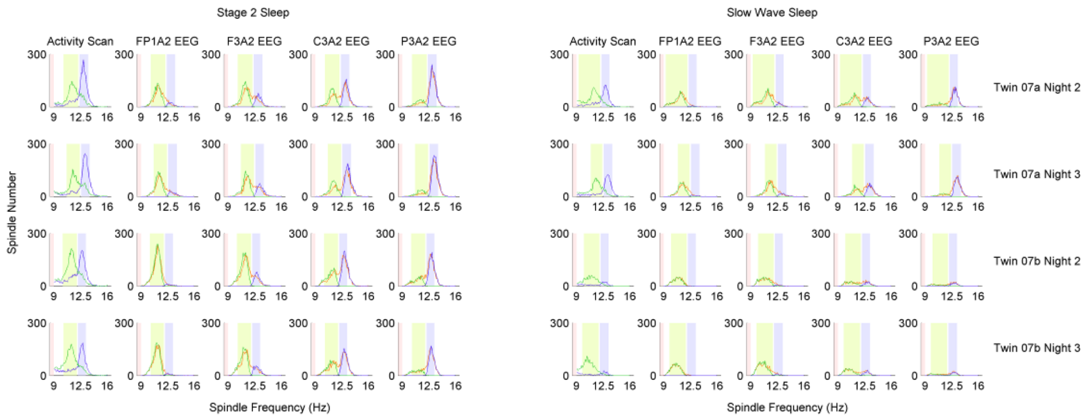

Supplementary Figure S35: Distribution of detected sleep spindles in 0.1 Hz frequency bins. Plots explanation as in Figure S1.

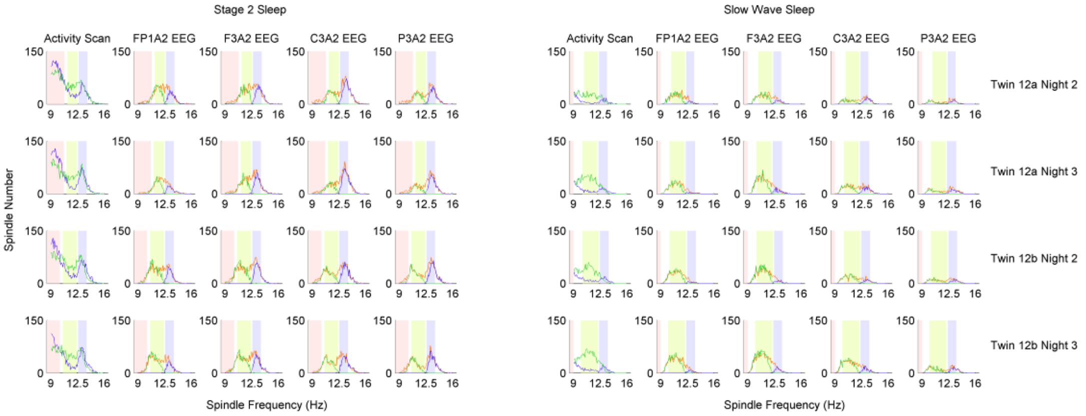

Supplementary Figure S36: Distribution of detected sleep spindles in 0.1 Hz frequency bins. Plots explanation as in Figure S1.

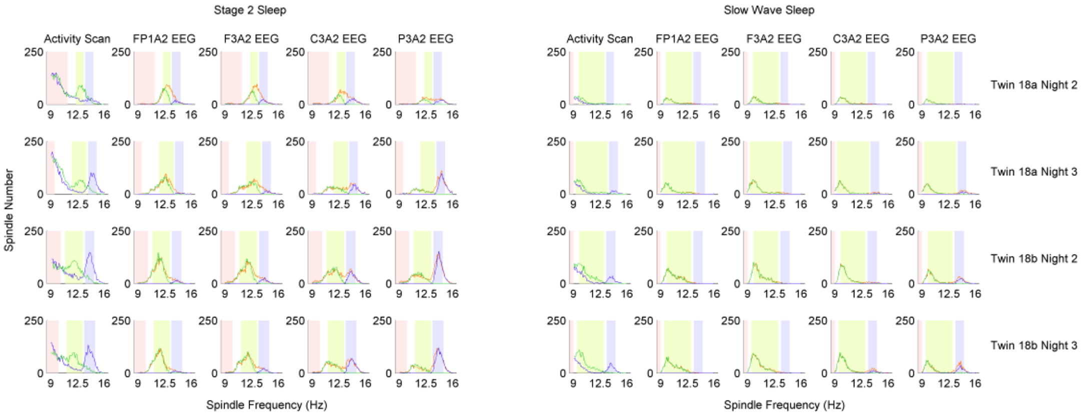

Supplementary Figure S37: Distribution of detected sleep spindles in 0.1 Hz frequency bins. Plots explanation as in Figure S1.

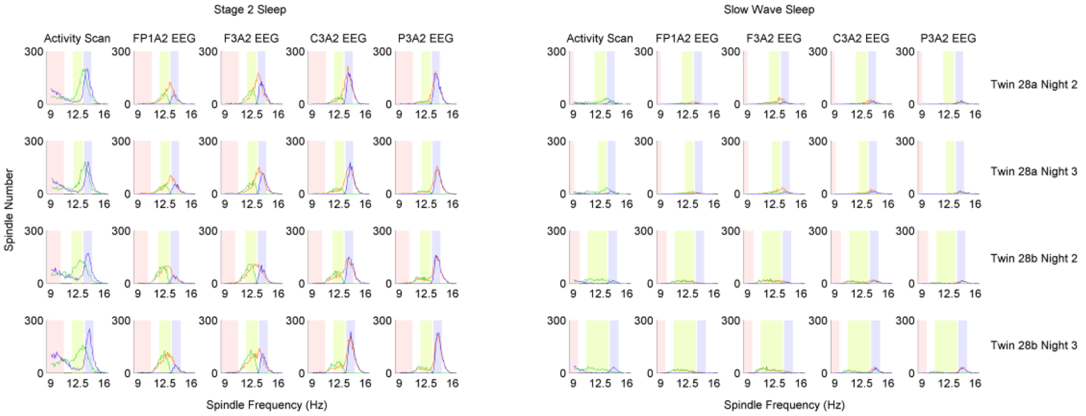

Supplementary Figure S38: Distribution of detected sleep spindles in 0.1 Hz frequency bins. Plots explanation as in Figure S1.

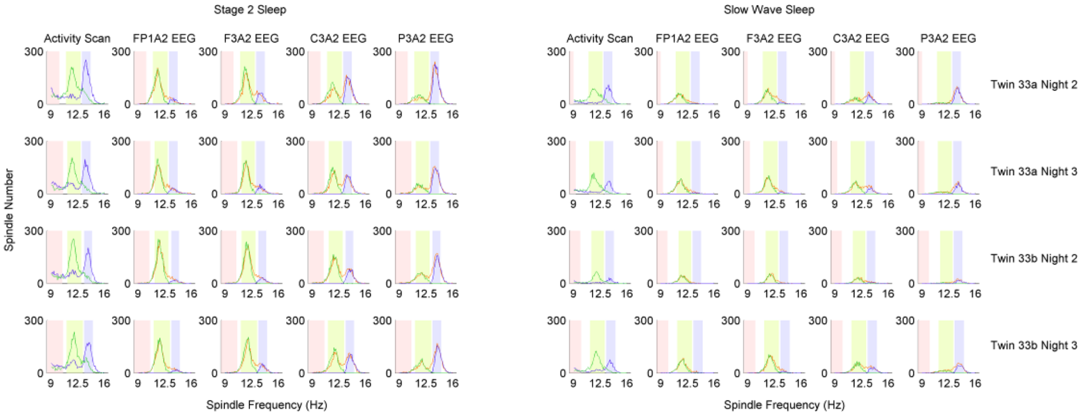

Supplementary Figure S39: Distribution of detected sleep spindles in 0.1 Hz frequency bins. Plots explanation as in Figure S1.

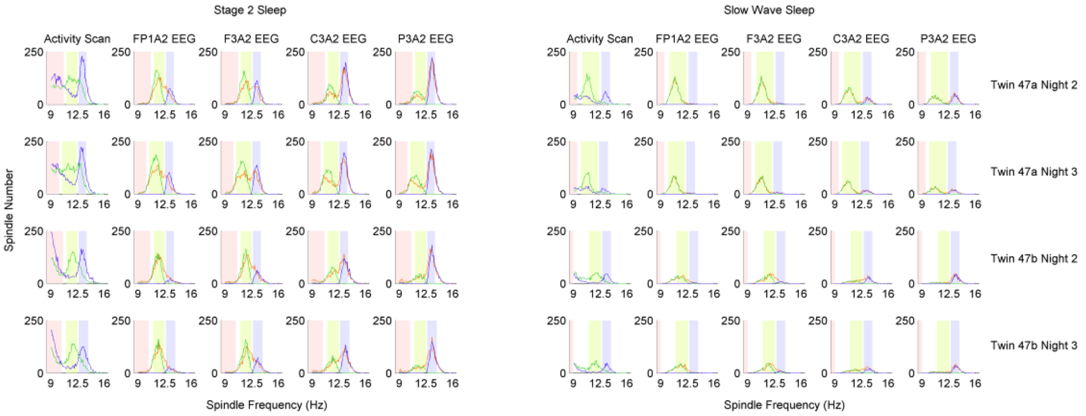

Supplementary Figure S40: Distribution of detected sleep spindles in 0.1 Hz frequency bins. Plots explanation as in Figure S1.

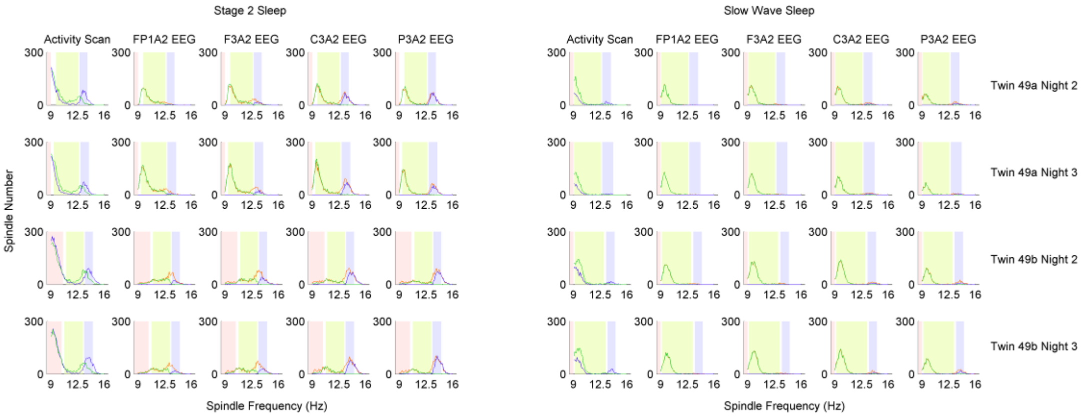

Supplementary Figure S41: Distribution of detected sleep spindles in 0.1 Hz frequency bins. Plots explanation as in Figure S1.

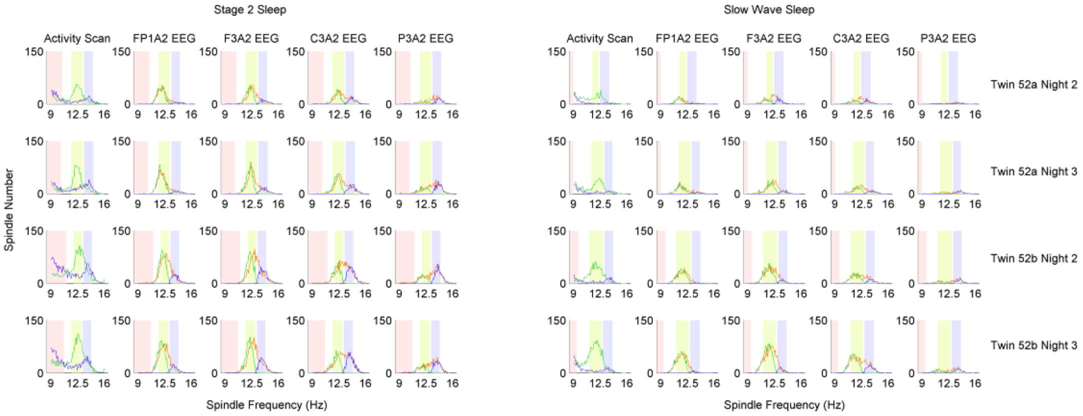

Supplementary Figure S42: Distribution of detected sleep spindles in 0.1 Hz frequency bins. Plots explanation as in Figure S1.

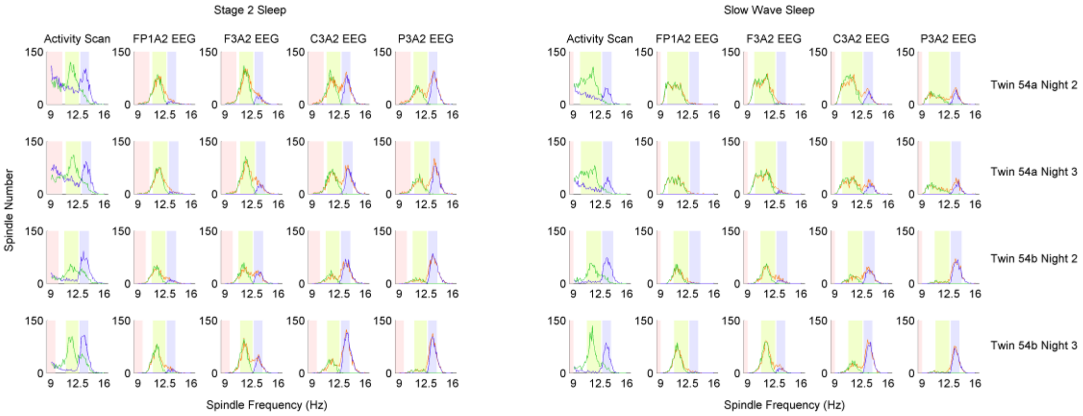

Supplementary Figure S43: Distribution of detected sleep spindles in 0.1 Hz frequency bins. Plots explanation as in Figure S1.

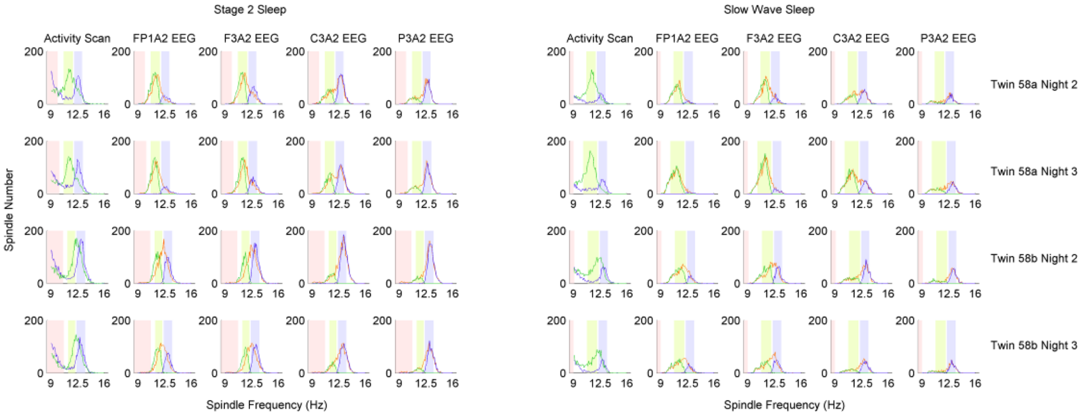

Supplementary Figure S44: Distribution of detected sleep spindles in 0.1 Hz frequency bins. Plots explanation as in Figure S1.

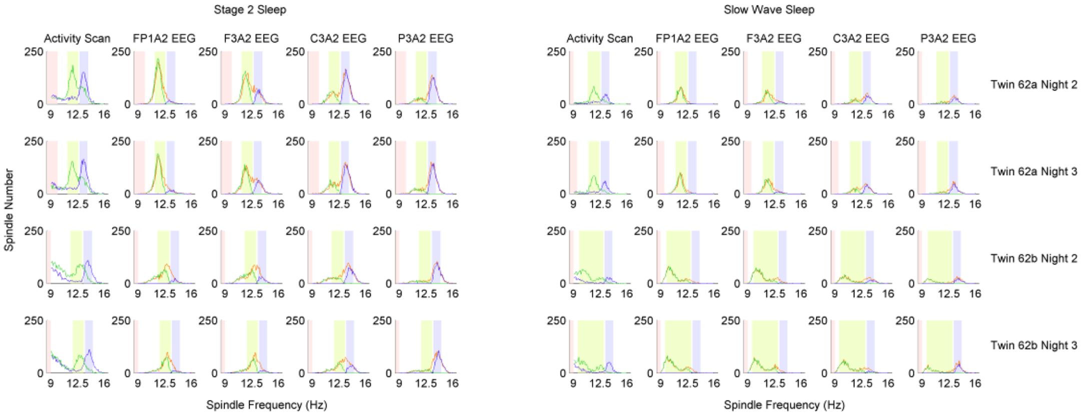

Supplementary Figure S45: Distribution of detected sleep spindles in 0.1 Hz frequency bins. Plots explanation as in Figure S1.

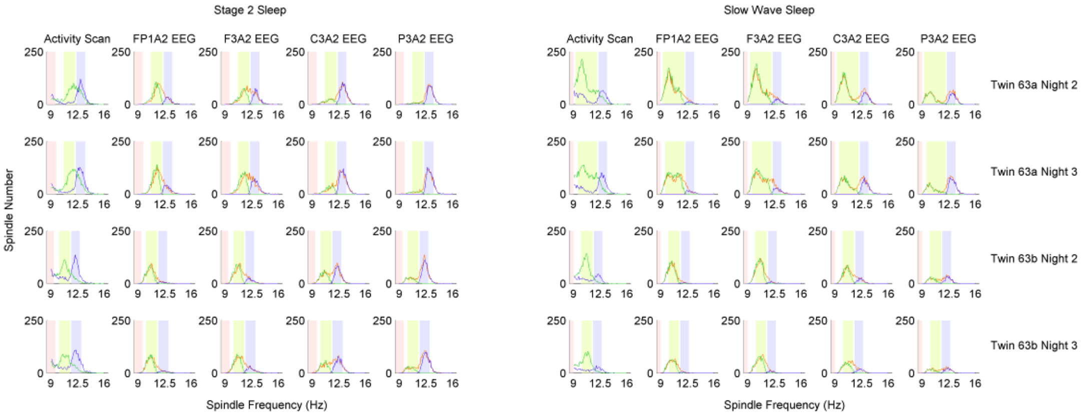

Supplementary Figure S46: Distribution of detected sleep spindles in 0.1 Hz frequency bins. Plots explanation as in Figure S1.
